# Insights into the trihelix transcription factor responses to salt and other stresses in *Osmanthus fragrans*

**DOI:** 10.1186/s12864-022-08569-7

**Published:** 2022-04-30

**Authors:** Meilin Zhu, Jing Bin, Huifen Ding, Duo Pan, Qingyin Tian, Xiulian Yang, Lianggui Wang, Yuanzheng Yue

**Affiliations:** 1 Key Laboratory of Landscape Architecture, Jiangsu Province, College of Landscape Architecture, Nanjing Forestry University, Nanjing, 210037 People’s Republic of China; 2 Co-Innovation Center for Sustainable Forestry in Southern China, Nanjing Forestry University, Nanjing, 210037 People’s Republic of China

## Abstract

**Background:**

*Osmanthus fragrans* is an evergreen plant with high ornamental and economic values. However, they are easily injured by salt stress, which severely limits their use in high salinity areas. The trihelix transcription factor (TF) family, as one of the earliest discovered TF families in plants, plays an essential part in responses to different abiotic stresses, and it has potential functions in improving the salt-tolerance capability of *O. fragrans*.

**Results:**

In this study, 56 trihelix genes (*OfGT*s) were first identified in *O. fragrans* and then divided into five subfamilies in accordance with a phylogenetic tree analysis. The *OfGT*s were found to be located randomly on the 20 *O. fragrans* chromosomes, and an analysis of gene replication events indicated that the *OfGT* gene family underwent strong purification selection during the evolutionary process. The analysis of conserved motifs and gene structures implied that the *OfGT* members in the same subfamily have similar conserved motifs and gene structures. A promoter *cis*-elements analysis showed that all the *OfGT* genes contained multiple abiotic and hormonal stress-related *cis*-elements. The RNA-seq data suggested that the *OfGT*s have specific expression patterns in different tissues, and some were induced by salt stress. The qRT-PCR analysis of 12 selected *OfGT*s confirmed that *OfGT1/3/21/33/42/45/46/52* were induced, with *OfGT3/42/46* being the most highly expressed. In addition, *OfGT42/OfGT46* had a co-expression pattern under salt-stress conditions. *OfGT3/42/46* were mainly localized in the nuclei and exhibited no transcriptional activities based on the analysis of the subcellular localization and transcriptional activity assay. Furthermore, the expression levels of most of the selected *OfGT*s were induced by multiple abiotic and hormonal stresses, and the expression patterns of some *OfGT*s were also highly correlated with gibberellic acid and methyl jasmonate levels. Remarkably, the transient transformation results showed lower MDA content and increased expression of ROS-related genes *NbAPX* in transgenic plants, which implying *OfGT3/42/46* may improve the salt tolerance of tobacco.

**Conclusions:**

The results implied that the *OfGT* genes were related to abiotic and hormonal stress responses in *O. fragrans*, and that the *OfGT3/42/46* genes in particular might play crucial roles in responses to salt stress. This study made a comprehensive summary of the *OfGT* gene family, including functions and co-expression patterns in response to salt and other stresses, as well as an evolutionary perspective. Consequently, it lays a foundation for further functional characterizations of these genes.

**Supplementary Information:**

The online version contains supplementary material available at 10.1186/s12864-022-08569-7.

## Background

Transcription factors (TFs) are proteins that can regulate transcription and expression of target genes by binding to specific DNA sequences [1]. Presently, more than 60 TFs have been discovered in plants [2]. A trihelix TF was first discovered to specifically bind to a light-responsive element, namely the GT component; consequently, it is also known as the GT factor family [3]. The common feature of trihelix TFs in plants is that the DNA-binding domain contains three helical structures (helix-loop-helix-loop-helix). The amino acid sequences of these functional domains are highly consistent and strongly conserved among the same subfamilies [4]. On the basis of the changes in the conserved domain, the trihelix TF is generally classified into five subfamilies: GT-1, SH4, GTγ, GT-2, and SIP1 [5]. Each subfamily contains an N-terminal conserved domain (apart from *At5g47660* in *Arabidopsis thaliana*), but the C-terminal domains are different. In addition, the subfamilies have only one DNA-binding domain, except the GT-2 subfamily, which contains two DNA-binding domains [6]. Although all the members contain at least one domain, there are subtle differences in this domain among different subfamilies. In the GT-1 and SH4 subfamilies, the trihelix domains each have a tryptophan residue in the internal hydrophobic region of the tandem repeat. In the GTγ and GT-2 subfamilies, the third conserved tryptophan is substituted by phenylalanine, whereas in the SIP1 subfamily, it is replaced by isoleucine [6, 7].

At present, the TF family of trihelix has been identified in *A. thaliana*, rice (*Oryza sativa*), *Fagopyrum tataricum*, *Phyllostachys edulis*, and *Populus trichocarpa*, and among them, *Arabidopsis* and rice have been studied in depth [2, 8–11]. The initial research on trihelix TFs showed that they help to regulate the light responses of plants [12, 13]. Additionally, the genes universally regulate multiple processes of plant growth and development, such as, embryo sac development [14], seed separation [15], and floral organ development [16]. Furthermore, studies have shown that trihelix TFs are also related to the response of plants to biotic and abiotic stresses, such as, salinity, drought and methyl jasmonate (MeJA) [17–19]. Interestingly, some trihelix genes participate in the responses to multiple stresses. In *Chrysanthemum morifolium*, the genes are affected by high salt, drought, low and high temperature, abscisic acid (ABA), and methyl jasmoate MeJA [20]. The salt tolerances of many plants have been improved by isolating and cloning trihelix TF genes related to salt tolerance. The GTγ subgroup members *OsGTγ-1, OsGTγ-2,* and *OsGTγ-3* in rice were induced by most of the abiotic stresses. Especially, overexpression of *OsGTγ-1* in rice enhanced salt tolerance at the seedling stage [21]. In soybean (*Glycine max*), *GmGT-2A* and *GmGT-2B* enhance the tolerance to salt stress [22]. In *A. thaliana*, the AtGT2L protein enhances plant tolerance to salt stress by up-regulating the expression levels of the marker genes *RD29A* and *ERD10* [17]. In addition, *AST1* which is a SIP1 subfamily TF member can combine with an AGAG-box or GT element to regulate downstream gene expression to enhance *Arabidopsis* salt-stress tolerance [23]. Interestingly, TFs also interact with other genes to enhance plant salt tolerance. For example, the *AtGT4* interacts with the *TEM2* gene to enhance the salt tolerance of *Arabidopsis* [24]. Some studies reported that MDA is closely related to cell membrane damage under abiotic stress [25]. Under cold stress, overexpressed *PubHLH1* and *NtbHLH123* have lower MDA content, which can reduce the oxidative damage of cell membrane by activating ROS-related genes [26, 27].

*Osmanthus fragrans* is an evergreen plant with high ornamental and economic values. Research on *O. fragrans* has focused on floral fragrance and flower color [28, 29], with research on abiotic stress tolerance being limited. High salinity and other abiotic stresses are critical adverse environmental factors that severely restrict plant growth and distribution [30]. However, the molecular regulatory mechanisms of *O. fragrans* involved in salt-tolerance and other abiotic stresses responses are still unclear. The publication of the whole-genome sequences of *O. fragrans* provides a resource for the screening of *O. fragrans* salt-tolerant genes and those involved in responding to other stresses [31].

In this research, 56 *OfGT* genes were screened from the *O. fragrans* genome data. They are located on 20 different chromosomes and were classified as five subfamilies. A thorough analysis of conserved motifs and gene structure was performed. In addition, the expression and co-expression patterns of 12 *OfGT* genes under three abiotic stress treatments (salt, waterlogging, and drought) and three hormonal stresses [MeJA, ABA, and gibberellic acid (GA_3_)] were examined. Furthermore, we analyzed the subcellular localizations and transcriptional activation activities of the potential salt tolerance genes *OfGT3/42/46*. Finally, the potential genes *OfGT3/42/46* was transferred into tobacco for functional verification by transient transformation. The study will provide a beneficial genetic resource for improving the salt tolerance of *O. fragrans.*

## Results

### Identification of *OfGT* gene family members in *O. fragrans*

We identified 56 trihelix genes from the database of *O. fragrans* genomes [31]. In accordance with their locations on the chromosomes, these genes were named *OfGT1–56*. The protein lengths encoded by the 56 *OfGT* genes range from 262 to 617 aa, with a mean length of 395 aa. The minimum isoelectric point value is 4.6, and the maximum is 9.58. The relative molecular masses range from 29.18 kDa to 69.75 kDa (Additional file 1: Table S1).

### Phylogenetic analysis and subcellular localization predictions for *OfGT* genes

**Fig. 1:**
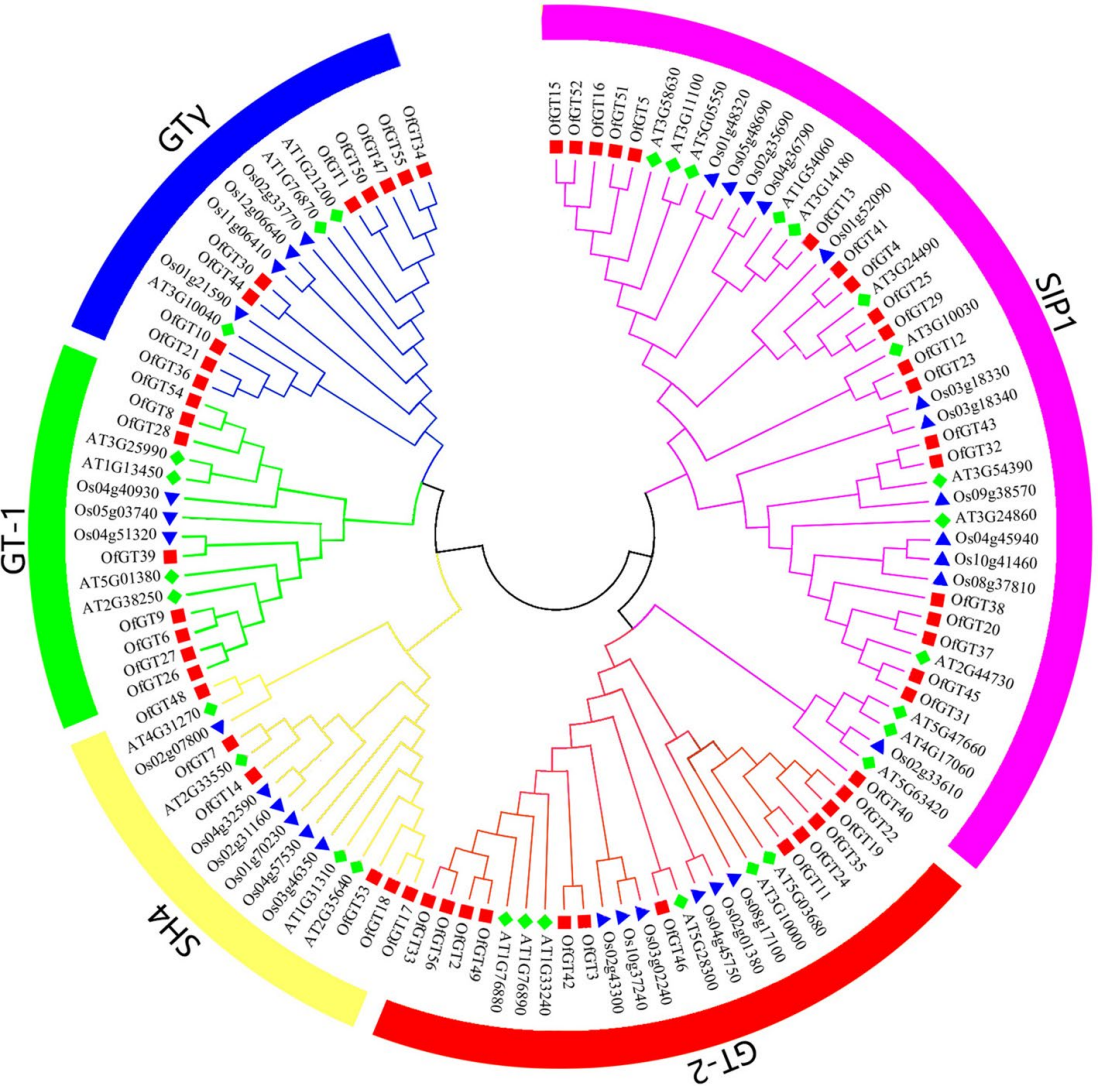
Phylogeny of trihelix proteins from *O. fragrans*, *A. thaliana* and *O. sativa*. The subfamilies of trihelix proteins are represented by different colors. The trihelix proteins of *O. fragrans*, *O. sativa*, and *A. thaliana* are marked with red squares, blue triangles, and green diamonds, respectively

To analyses the evolutional relationships between the 56 OfGT proteins, a phylogenetic tree was constructed with the trihelix genes of rice (*O. sativa*) and *Arabidopsis* (Fig. 1, Additional file 2: Table S2). In accordance with the classifications of trihelix TFs in rice and *A. thaliana*, the 56 trihelix genes in *O. fragrans* were classified into five subfamilies (GT-1, SH4, GTγ, GT-2, and SIP1). The largest subfamily is SIP1, which contains 20 members. The smallest subfamily is SH4, with only six members.

The subcellular localization predictions for the 56 OfGT protein sequences revealed that most (80.36%) of the proteins are localized in the nucleus, a small portion (14.29%) are localized in the chloroplasts, OfGT26 and OfGT27 (3.57%) are localized in the cytoplasm, and OfGT48 (1.79%) is localized in the mitochondria (Additional file 3: Table S3).

### The analysis of genes structures, motif compositions, and a promoter for the *OfGT* gene family

**Fig. 2:**
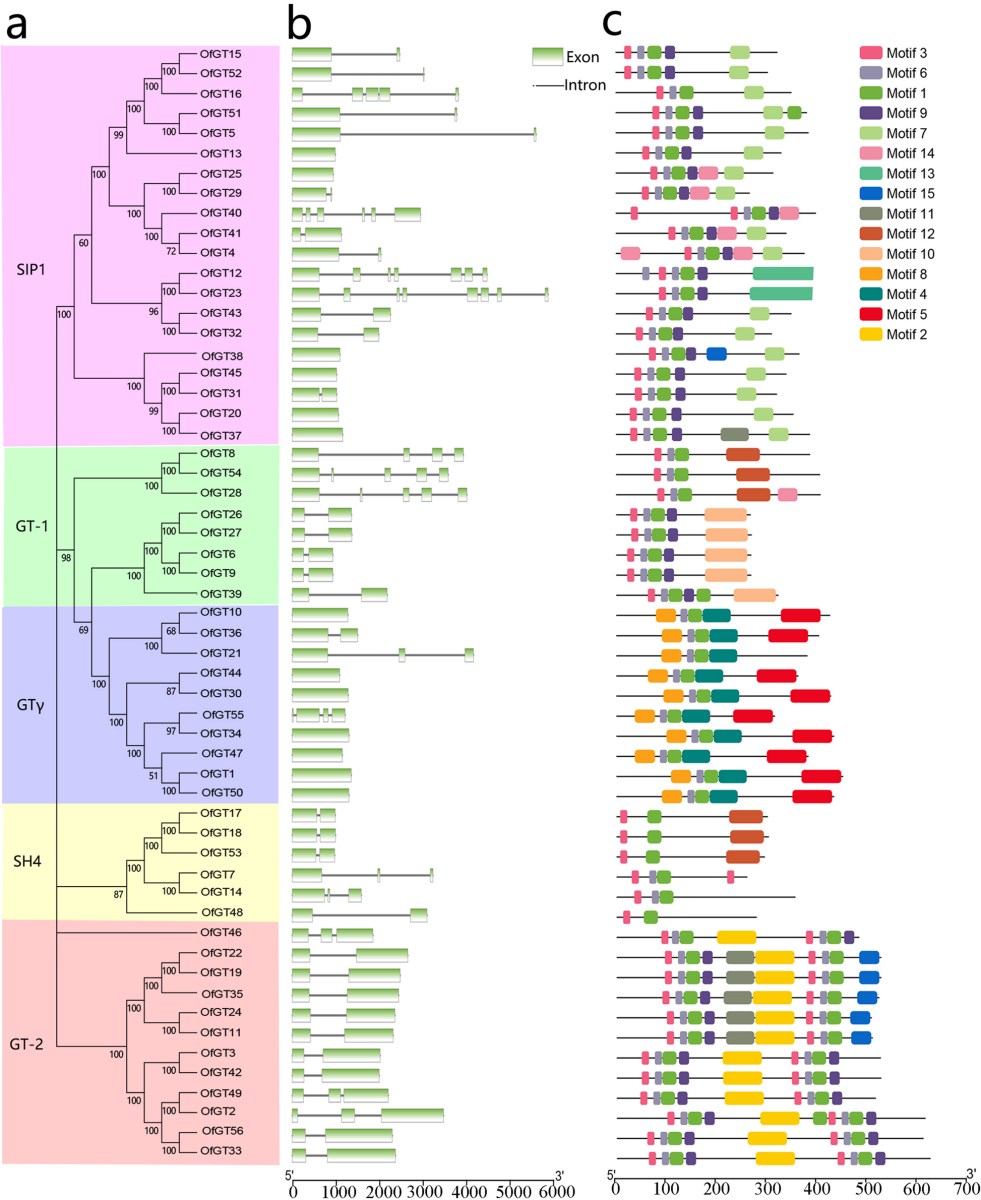
Phylogenetic relationships, gene structures, and motif compositions of *OfGT* genes. **a** Unrooted neighbor-joining phylogenetic tree. The specific subfamilies are marked with specific colors. **b** Exons are represented by green boxes and introns are marked with gray lines. **c** Each colored box indicates a motif, and non-conserved sequences are shown by black lines

According to the exon/intron structure analysis, 56 *OfGT* genes contain one to eight exons. In total, 23% of *OfGT* genes lack introns (Fig. 2a-b). The majority of genes clustered in the identical subfamily showed similar exon/intron structures. For example, in the GT-2 subfamily, nine members contained two exons, whereas *OfGT2/46/49* contained three exons. Using MEME, 15 motifs were identified among the 56 OfGT proteins (Fig. 2c). Specific amino acid sequences for each motif are provided (Additional file 4: Table S4). All the OfGT proteins contain motif 1, which is the most conserved motif among the subfamilies. The OfGT proteins, except for the GTγ branch members, contain motif 3. The GT-2 branch genes have two trihelix domains. In addition, the conserved motifs of the most closely related members of the phylogenetic tree showed similar arrangements and positions, indicating that the functions of the trihelix proteins in each defined subfamily may be similar.

**Fig. 3:**
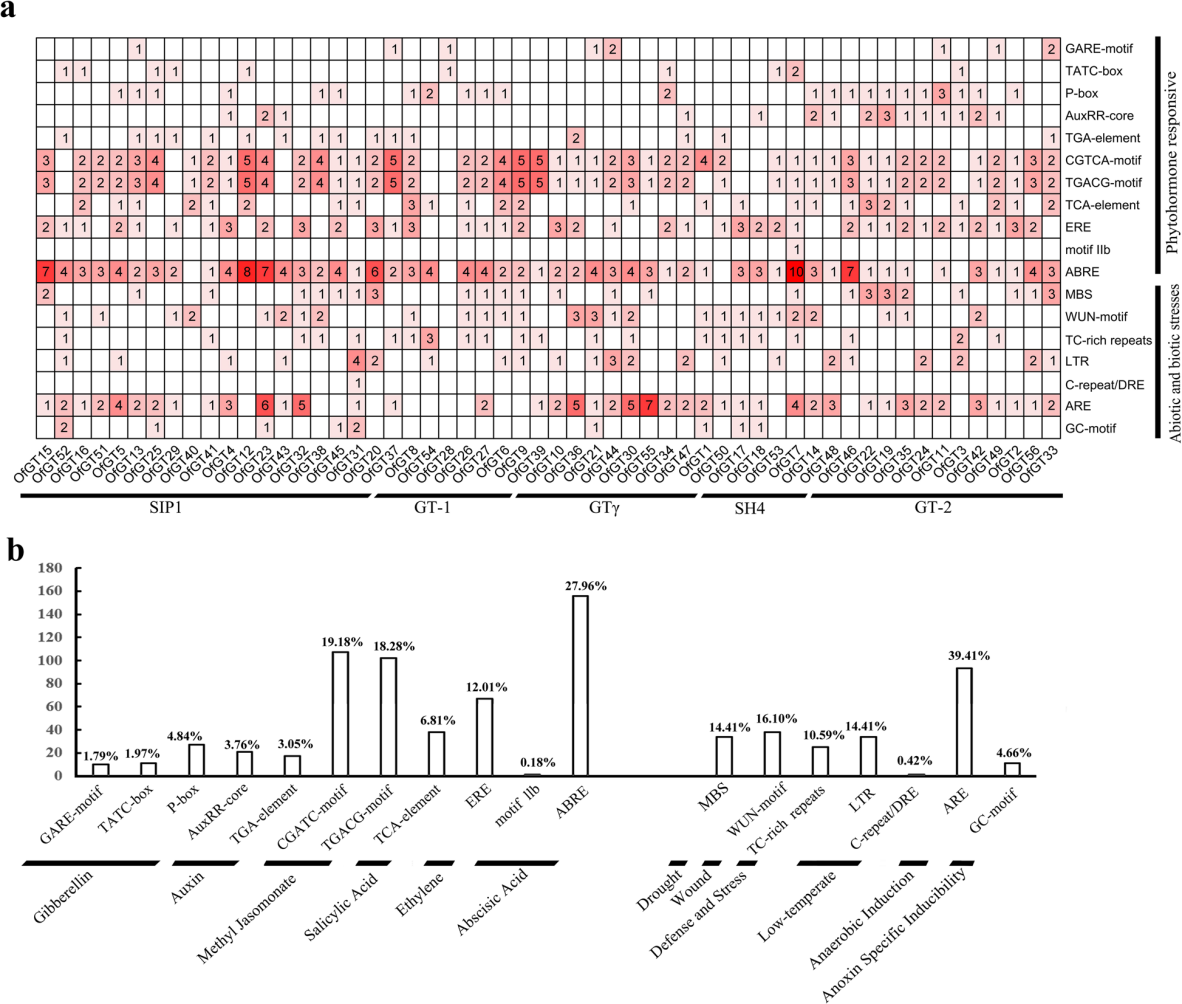
The analysis of *cis*-elements in the promoter regions of *OfGT* genes. **a** The total number of *cis*-elements for each *OfGT* gene is indicated by the numbers. **b** The categorization of *cis*-acting elements relevant to biotic and abiotic stresses and plant hormone responses. The percentages and quantities of factors in each category are presented

In the promoter analysis, four categories of *cis*-elements were discovered in the *OfGT* genes (Fig. 3a–b). The first category is plant hormone-response elements, including the gibberellin-response element GARE-motif (1.79%), TATC-box (1.97%), and P-box (4.84%); MeJA (37.46%) homeopathic element CGTCA motif (19.18%) and TGACG motifs (18.28%), ABA homeopathic elements motifIIb (0.18%) and ABRE (21.96%); *cis*-regulatory elements involved in salicylic acid-response TCA element (6.81%); and auxin *cis*-response regulatory elements AuxRR-core (3.76%) and TGA element (3.05%). The second category is abiotic and biotic stress-response elements, including drought (14.41%) and low temperature (0.42%) response elements, as well as defense and stress responses (TC-rich repeats, 10.59%), and anaerobic induction (ARE, 39.41%). The third category is plant growth and developmental factors, such as *cis-*elements involve in the specific activation of meristem (CCGTCC-box) and endosperm expression *cis*-regulating elements (Skin-1_motf and GCN4_motif). The last category consists of light-responsive elements, such as GT1 motif, G-box, and Sp1 (Additional file 5: Table S5). The above analysis of the hormonal and abiotic stress-response elements of the *OfGT* genes provides a basis for the subsequent hormonal and stress treatment of plants.

### The gene duplication events of *OfGT* genes and distribution on the chromosomes

**Fig. 4:**
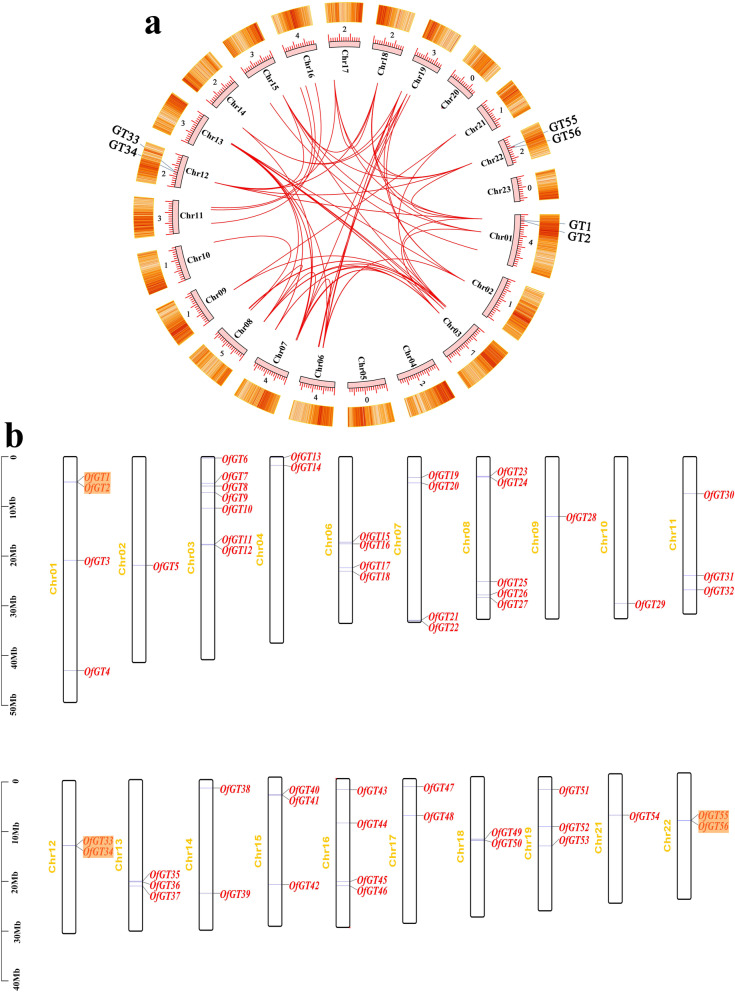
The analysis of the *OfGT* chromosomal distribution and duplication events. **a** The relationships highlighted by red lines represent the 44 segmental duplications of *OfGT* genes. The three pairs of genes marked on the outer ring of the circle are tandem replicated genes. The heatmaps in the outer orange rectangle represent the gene density levels on the chromosomes. The value on the upper part of each chromosome shows the total number of genes it contains. **b** The distribution of the 56 *OfGT* genes on chromosomal *O. fragrans*

The MCScanX was used to analyze gene replication events, including tandem repeats and fragment replication. Three pairs of tandem repeat genes (*OfGT1/OfGT2*, *OfGT33/OfGT34*, and *OfGT55/OfGT56*) were identified. The 44 duplicated genes were also identified among the 56 *OfGT* genes (Fig. 4a). Furthermore, the *OfGT* gene pairs resulting from gene duplication events, non-synonymous (Ka)/synonymous (Ks) substitution rate ratios were < 1, which indicated that the *OfGT* genes have undergone strong purification selection during the evolutionary process (Additional file 6: Table S6). In addition, the 56 *OfGT* genes are scattered across the 20 chromosomes (Fig. 4b). The *OfGT* genes numbers distributed on each chromosome ranges from one to seven. The most *OfGT* genes are located on Chr03, whereas Chr05, Chr20, and Chr23 do not contain any *OfGT* gene.

### Expression pattern of *OfGT* genes in different tissues

**Fig. 5:**
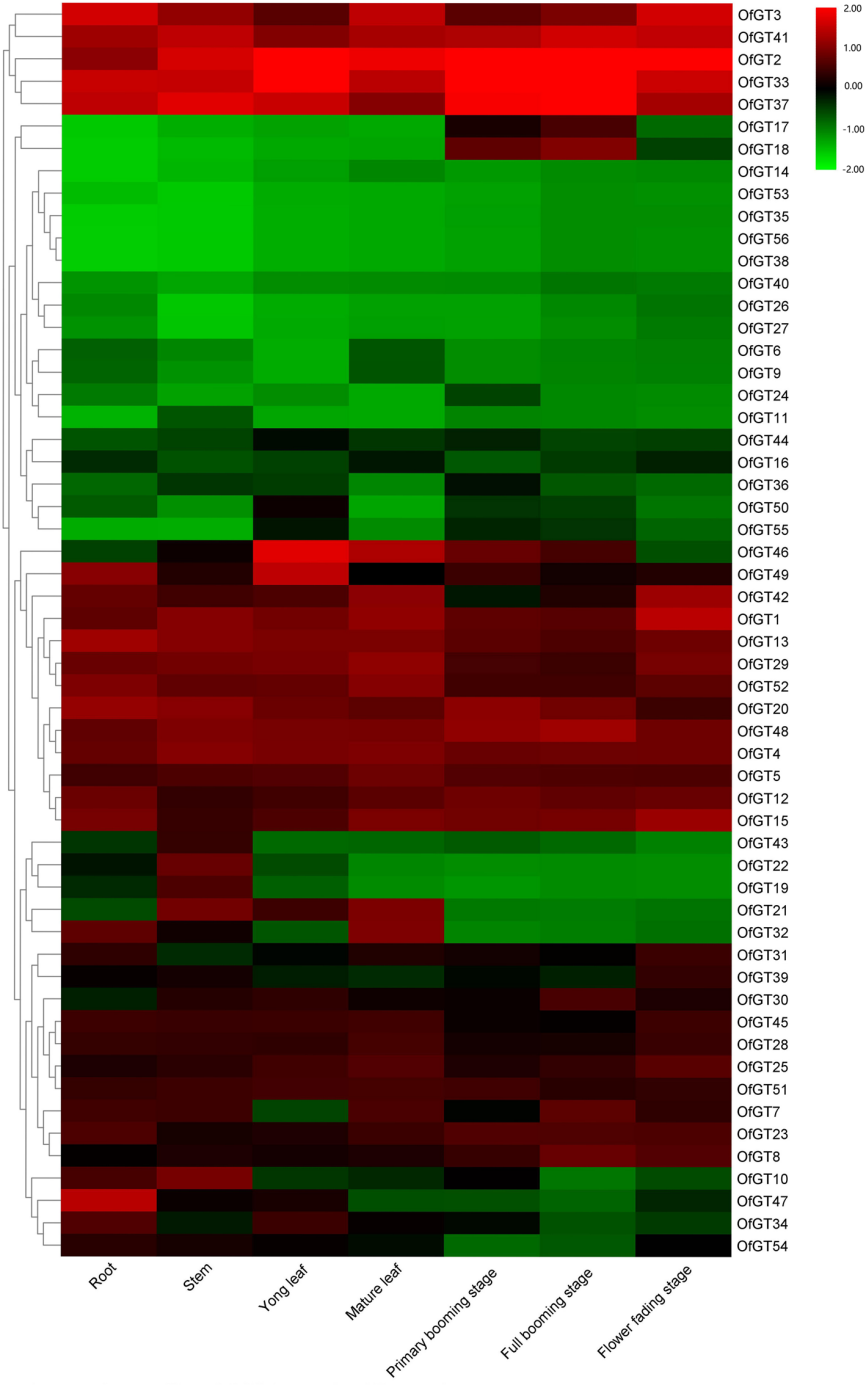
The expression pattern of the *OfGT* genes in seven tissues and developmental stages. The RPKM values were converted to log_2_(value)

The RPKM values of 56 *OfGT* genes were derived from the *O. fragrans* transcriptome databases of seven different tissue samples [31]. On the basis of the cluster analysis, the 56 *OfGT* genes were roughly divided into five different groups, and these genes in the same cluster having similar expression values. The overall expression revealed that 16 genes are expressed in all samples, 17 *OfGT* genes have different expression patterns in seven tissues, and 19 *OfGT* genes were not expressed in any tissue (Fig. 5). Some of the *OfGTs* genes showed tissue-specific expression. For example, *OfGT47* is only expressed in roots, and *OfGT19/22/43* are only expressed in stems. Most SIP1 subfamily members had higher expression levels in different tissues.

### Expression analysis of *OfGT* genes in response to salt

**Fig. 6:**
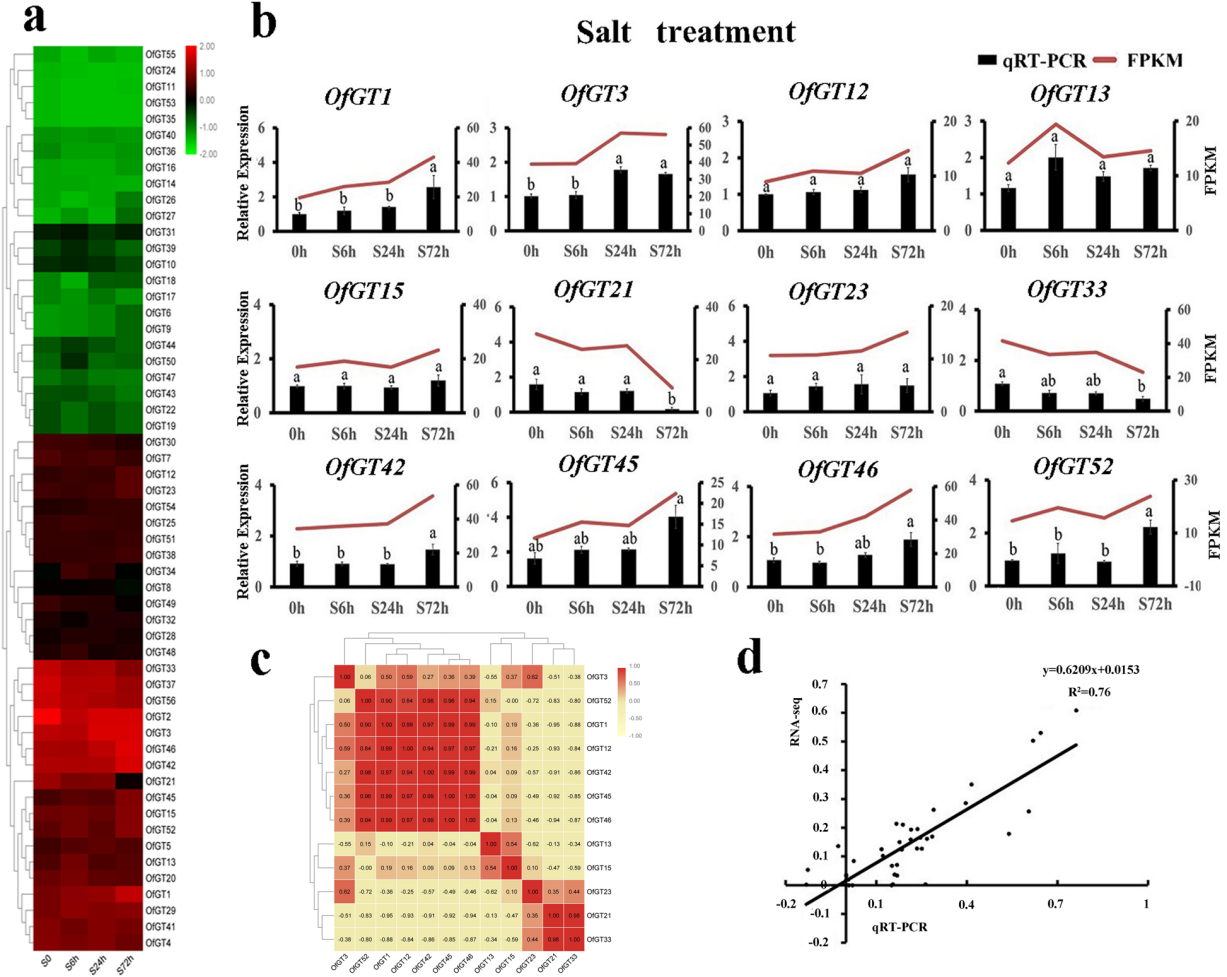
Expression levels of the 12 *OfGT* genes’ responses to salt stress conditions. **a** The following periods of stress were used: control at 0 h (S0) and salt treatments for 6 h (S6h), 24 h (S24h), and 72 h (S72h). The FPKM values were converted to log_2_(value). **b** Expression levels of the 12 *OfGT* genes related to the salt-stress treatment were examined by qRT-PCR. The black rectangles represent the relative expression levels, and the red lines represent the FPKM values. Significant differences were evaluated using Duncan’s test (*p* < 0.05). The SEs are presented as the values of the bars. **c** Correlation analysis of 12 *OfGT* genes under salt-stress conditions. The values represent the Pearson’s correlation coefficient. *r* > 0: positively correlated, *r* < 0: negatively correlated. The larger the absolute value of *r*, the stronger the correlation. **d** The correlation analysis chart of qRT-PCR and transcriptome data FPKM values

The FPKM values of the 56 *OfGT* genes were derived from the transcriptome data of *O. fragrans* leaves treated with salt stress (Additional file 7: Table S7). Here, the *OfGT* genes that may be involved in the salt-stress response were initially screened. In total, the expression levels of 12 genes (*OfGT1/3/12/13/15/21/23/33/42/45/46/52*) were higher (FPKM > 10) after the salt treatment and showed obvious changes (Fig. 6a). Then, qRT-PCR was used to determine whether these 12 genes were responded to salt stress. Under salt-stress conditions, the expression levels of *OfGT1/3/42/45/46/52* were obviously upregulated. In particular, the FPKM values of the *OfGT3/42/46* genes were highest after a 72 h salt treatment. In addition, the expression levels of *OfGT21/23* obviously decreased, whereas the expression levels of the *OfGT12/13/15/23* genes showed no changes under salt-stress conditions (Fig. 6b). The expression patterns of these 12 genes showed that *OfGT1/12/42/45/46/52* were strongly positively correlated, which indicates that these genes might enhance the tolerance to salt stress through cooperative effects (Fig. 6c). In general, the expression change trends of these 12 genes were basically the same as the change trends of their corresponding transcriptome FPKM values. A correlation analysis chart of the relative expression values and transcriptome data FPKM values showed a high correlation coefficient (*r*^2^ = 0.76) (Fig. 6d), indicating the reliability of the transcriptome data.

### Expression analysis of *OfGT* genes in response to other stresses

**Fig. 7:**
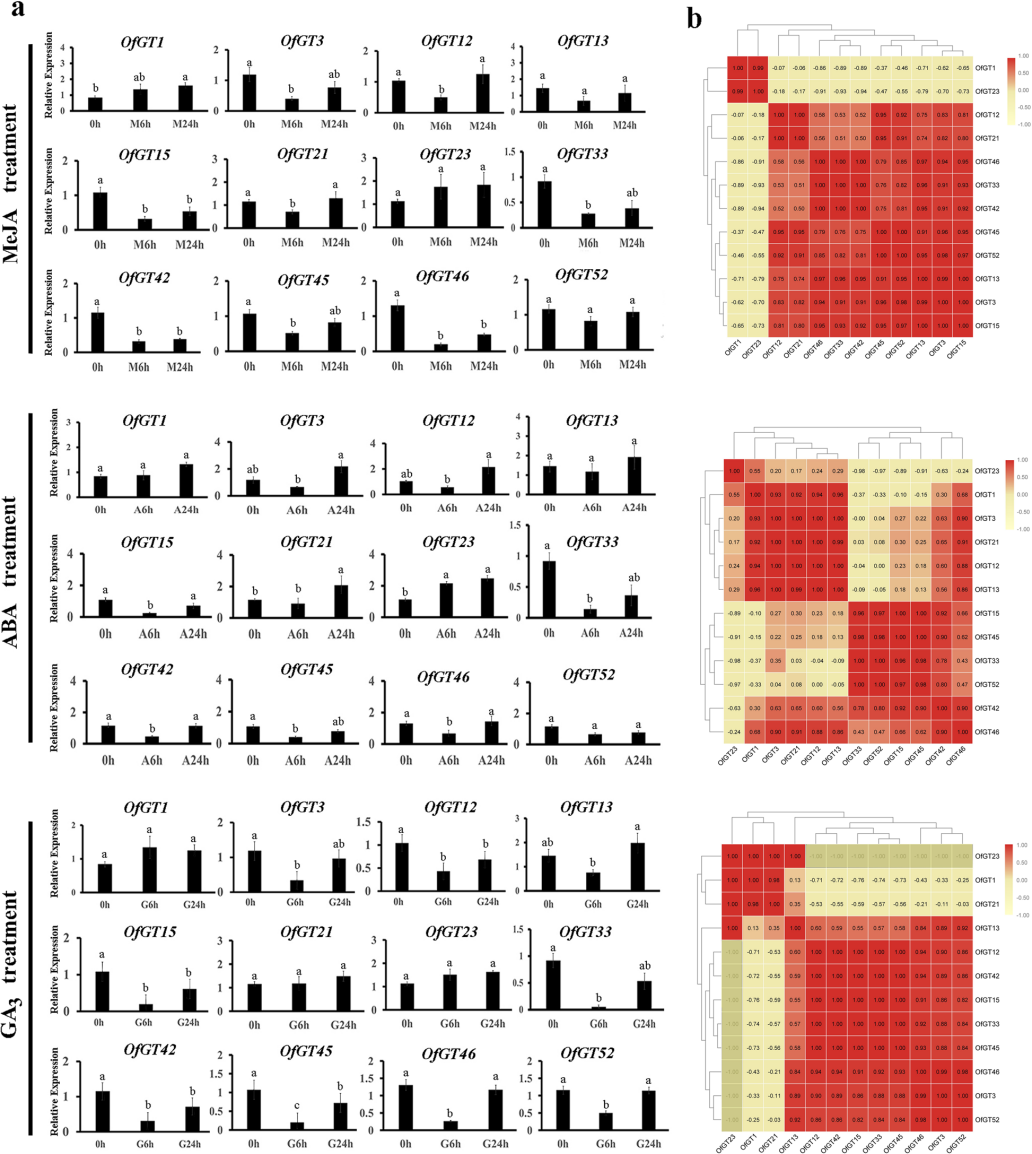
Expression patterns of the 12 *OfGT* genes in response to drought/waterlogging stress. **a** Expression levels of the 12 *OfGT* genes under the drought/waterlogging stress treatments were examined by qRT-PCR. The black rectangles represent the relative expression levels. Significant differences were evaluated using Duncan’s test (*p* < 0.05). The SEs are represented as the values of the bars. The specific stress times: control at 0 h (0 h) and drought/ waterlogging treatments for 6 h (6 h), 24 h (S24h/D24h), and 72 h (W72h/D24h). **b** Correlation analysis of 12 *OfGT* genes under drought/ waterlogging stress conditions

To further explore whether these 12 *OfGT* genes only respond to salt stress or respond to multiple stresses, the genes’ expression profiles under drought and waterlogging stresses were analyzed. For drought stress, after 6 h of a PEG-6000 treatment, the expression levels of *OfGT3/13/45/46/52* decreased and then gradually increased. *OfGT12/15/42* were down-regulated after the drought treatment. In contrast, the *OfGT1* gene showed up-regulated expression. The *OfGT21/3/33* gene expression levels remained unchanged (Fig. 7a). The expression profiles of 12 genes under drought-stress conditions revealed that the *OfGT3/13/46/52* gene group and the *OfGT12/15* and *OfGT33/45* gene pairs had strong positive correlations (Fig. 7b). For waterlogging treatment, the expression levels of *OfGT12/13/15/42/45/52* showed increasing to decreasing trends. The levels of *OfGT21/23* remained the same at the beginning and then declined after 24 h of treatment. The *OfGT1/3//33/46* genes’ expression levels showed no significant changes under waterlogging-stress conditions (Fig. 7a). The correlation analysis of 12 genes subjected to waterlogging stress revealed that *OfGT1/12/13/45* gene group, and the *OfGT3/21* and *OfGT23/52* gene pairs, had strong positive correlations (Fig. 7b).

**Fig. 8:**
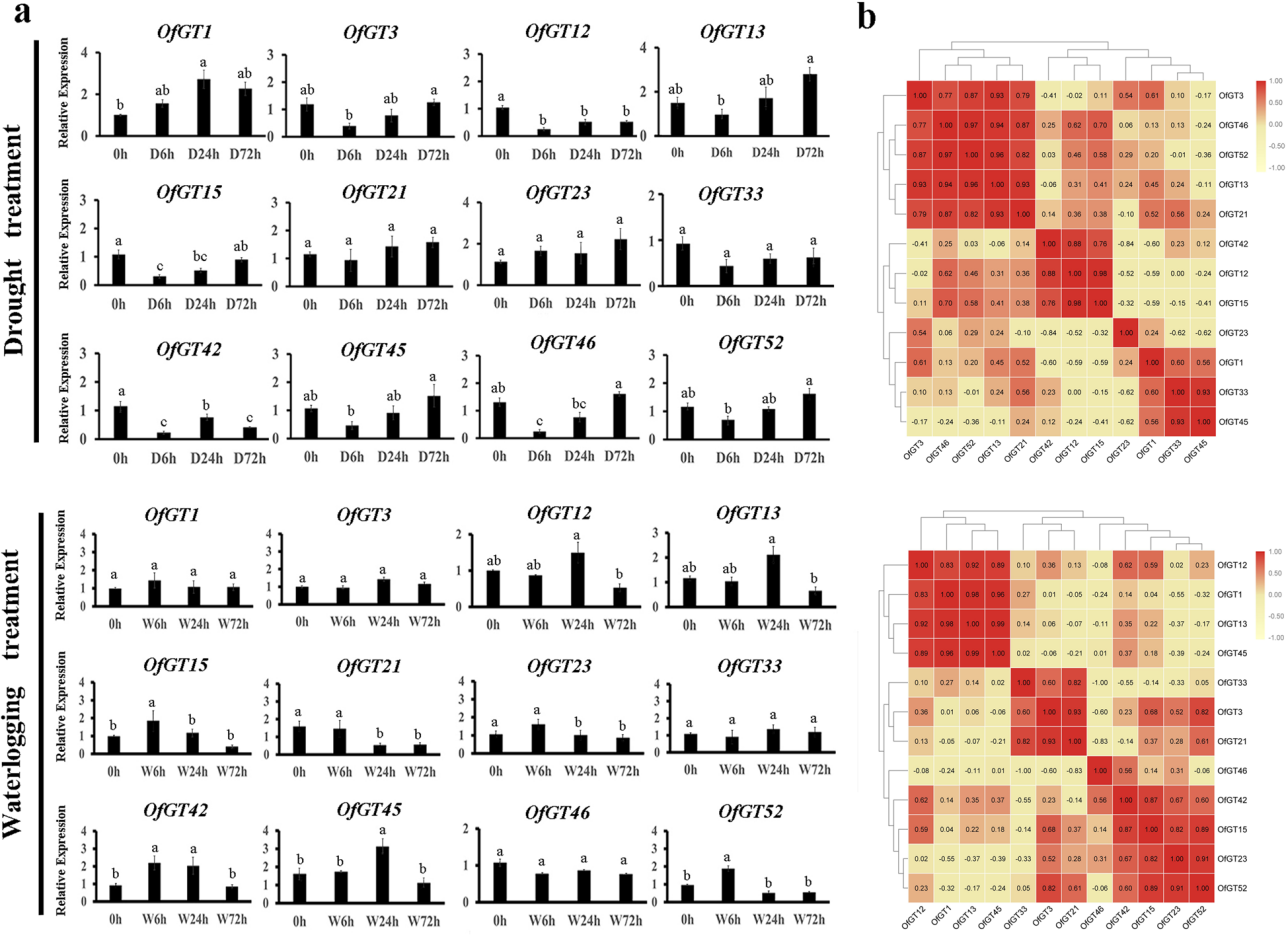
Expression profiles of the 12 *OfGT* genes in response to a MeJA/ABA/GA_3_ treatments. **a** Expression profiles of the 12 *OfGT* genes in response to a MeJA/ABA/GA_3_ treatments were examined by qRT-PCR. The black rectangles represent the relative expression levels. Significant differences were evaluated using Duncan’s test (*p* < 0.05). The SEs are represented as the values of the bars. The specific stress times: control at 0 h (0 h) and MeJA/ABA/GA_3_ treatments for 6 h (M6h/A6h/G6h), 24 h (M24h/A24h/G24h), and 72 h (M72h/A72h/G72h). **b** Correlation analysis of 12 *OfGT* genes after MeJA/ABA/GA_3_ treatments

To further investigate whether *OfGT* genes respond to hormonal stresses, we treated *O. fragrans* seedlings with MeJA, ABA, and GA_3_. The expression trends of the 12 *OfGT* genes, under different hormonal treatments were roughly the same, which indicated that they slowly dropped after 6 h of treatment and gradually rose after 12 h of treatment (Fig. 8a). The integration of expression data under multiple treatments have been used to perform the co-expression analysis (Additional file 8: Fig. S1). Under MeJA-stress conditions, the expression patterns of 12 genes showed that the *OfGT33/42/46* and *OfGT3/13/15/45/52* gene groups, as well as the *OfGT1/23* and *OfGT12/21* gene pairs have strong positive correlations (Fig. 8b). The expression relationship of 12 genes under ABA-stress conditions showed that the *OfGT1/3/12/13/21* and *OfGT15/33/42/45/52* gene groups, and *OfGT42/46* gene pair have strong positive correlations. In addition, *OfGT23* had a robust negative correlation with *OfGT15/33/45/52* after the ABA treatment (Fig. 8b). For GA_3_ treatment, the *OfGT3/12/15/33/42/45/46/52* gene group are strongly positively correlated (Fig. 8b). Thus, some of *OfGT* genes had co-expression patterns under different stresses. We speculated that these *OfGT* genes improve tolerance levels to a specific stress through cooperative relationships in *O. fragrans.* In particular, the positive correlations of 12 *OfGT* genes was more obvious after GA_3_ and MeJA treatments, which indicates that they may have closer cooperative relationships under these conditions. Furthermore, most of selected *OfGT*s were induced by multiple abiotic and hormonal stresses. For example, *OfGT3/42/46* were induced by salt, drought, MeJA, ABA, and GA_3_ stresses.

### Subcellular localizations and transcriptional activation activities of *OfGT3/42/46*

**Fig. 9:**
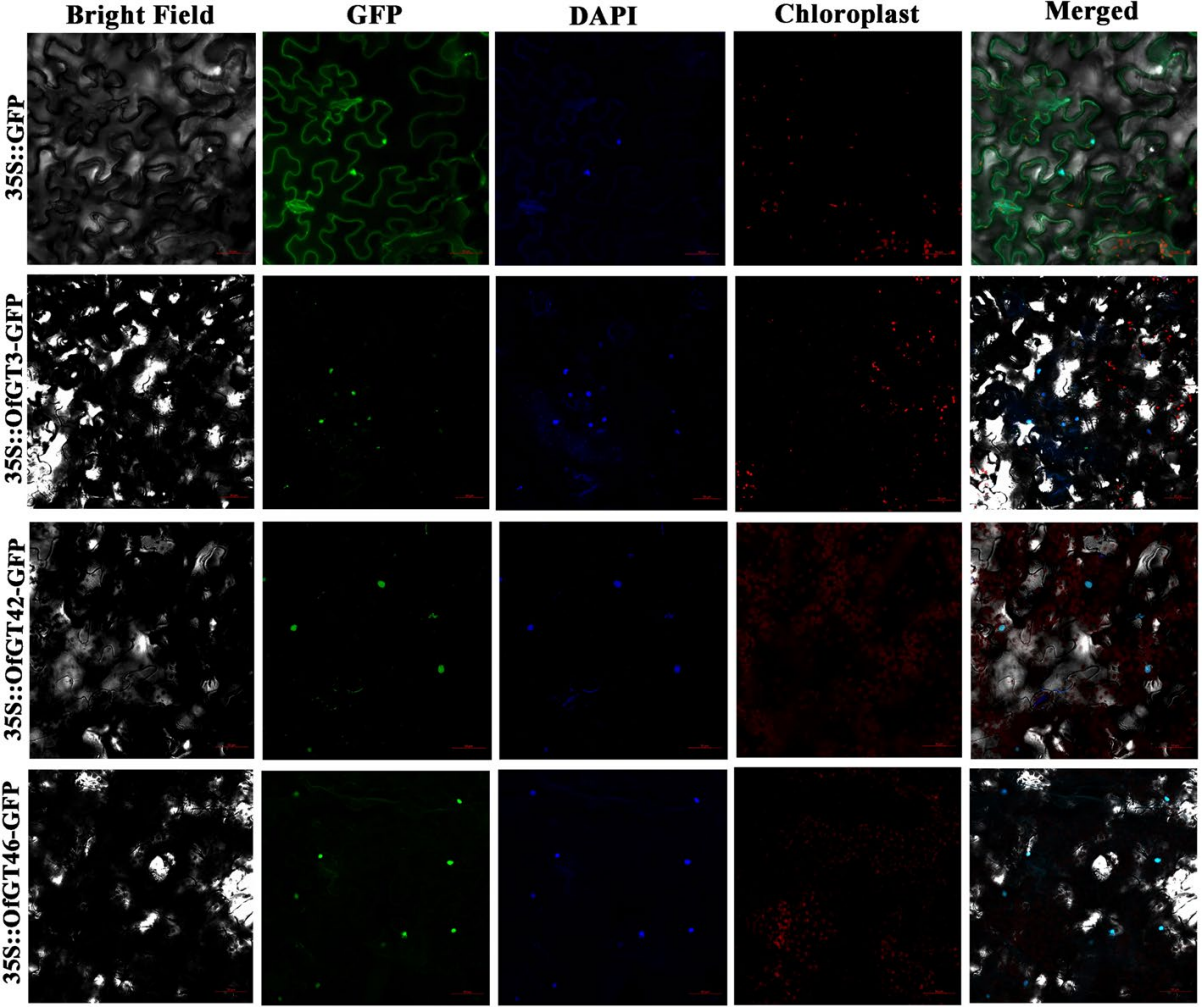
Subcellular localizations of *OfGT3/42/46*. The GFP signals in epidermal cells separated from *N.benthamiana* leaves were observed using an LSM710 microscope. The nuclei are marked by DAPI staining

On the basis of the FPKM values and expression level of *OfGT3/42/46* genes were relatively high, they also have high degrees of homology with the salt-tolerant members of the trihelix TF family reported in soybean [22]. Therefore, we selected the *OfGT3/42/46* genes for further study. The constructed GFP::pCAMBIA1300–3/42/46 fusion vectors and the 1300 empty vector were independently transiently transformed into tobacco (*Nicotiana benthamiana*) leaves. As shown in Fig. 9, the protein-coding nucleotide products of the three genes *OfGT3*, *OfGT42* and *OfGT46* in the trihelix TF family of *O. fragrans* were mainly expressed in the nuclei.

The transcriptional activation of *OfGT3/42/46* was determined by constructing pGBKT7 vectors that were then transformed into yeast strain AH109. The positive control grew well on SD/−Trp, SD/−Trp-Ade, and SD/−Trp-Ade + X-α-gal media and produced a blue color. In contrast, yeast strains transformed with the negative control pGBKT7 vector and the *OfGT3/42/46* pGBKT7 vectors were only able to grow well on the SD/−Trp culture medium. They did not grow on the SD/−Trp-Ade and SD/−Trp-Ade + X-α-gal media, and there were no blue color-producing reactions (Additional file 9: Fig. S2). The results indicated that *OfGT3/42/46* were not active in the yeast strain AH109.

### The analysis of malondialdehyde content and the transient expression of *OfGT3/42/46*

**Fig. 10:**
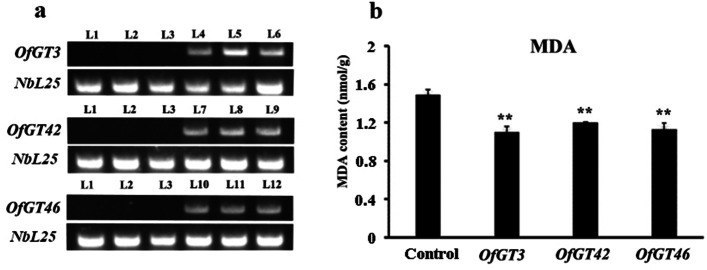
The content of MDA and the expression of *OfGT3/42/46* in tobacco. Significant differences were evaluated using Duncan’s test (**, *p* < 0.01). The SEs are presented as the values of the bars. **a** the *NbL25* gene was amplified as an internal control of tobacco. L1–3, L4–6, L7–9, and L10–12 represent the three lines of tobacco injected with the pCAMBIA1300 vector (control), pCAMBIA1300-*OfGT3*, pCAMBIA1300-*OfGT42*, and pCAMBIA1300-*OfGT46*, respectively **b** Under salt treatment, the MDA content in tobacco transformed with *OfGT3/42/46* genes

Malondialdehyde (MDA) is a membrane lipid peroxidation product [32]. By determining the levels of MDA, the degree of damage to the cell membrane can be evaluated [33]. Under salt stress, the MDA content of tobacco infected with pCAMBIA1300-OfGT3/42/46 was lower than that of the control (pCAMBIA1300), and the difference reached a significant level (Fig. 10b). The results indicate that the cell membrane of tobacco infected by pCAMBIA1300-OfGT3/42/46 is less damaged than that in the control plants, indicating that the transient transformation of *OfGT3/42/46* might enhance the salt tolerance of tobacco. In addition, the results of semi-quantitative RT-PCR strongly proved that the pCAMBIA1300-OfGT3/42/46 fusion protein was expressed in tobacco (Fig. 10a). The original uncropped gel (Fig. 10a) of qRT-PCR analysis was provided in the additional files (Additional file 10: Fig. S3).

**Fig. 11:**
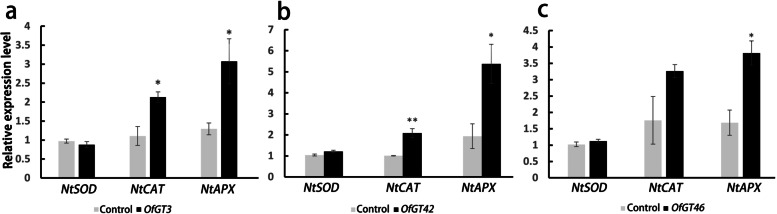
The relative expression level of *NbSOD/NbCAT/NbAPX* in tobacco. Significant differences were evaluated using Duncan’s test (*, *p* < 0.05; **, *p* < 0.01). The SEs are presented as the values of the bars

To further explore the functions of *OfGT* family, the qRT-PCR analysis of the ROS-related genes (*NbAPX*, *NbCAT*, and *NbSOD*) in the *OfGT*3/42/46 and empty vector transient expression tobaccos were conducted (Fig. 11). The expression of *NbSOD* in *OfGT3/42/46* overexpressing plants and empty vector (control) did not reach significant levels. Notably, the expression level of *NbAPX* was significantly up regulated in *OfGT3/42/46* overexpressing plants compared to control, in addition, the expression level of *NbCAT* was significantly higher in *OfGT3/42* overexpressing plants than the control.

## Discussion

Many researchers have systematically identified the trihelix TF gene family in various plants, such as *A. thaliana*, *P. edulis, Camellia sinensis*, and *P. trichocarpa* [10, 11, 34, 35]. However, no comprehensive research on the trihelix TF gene family of *O. fragrans* has been reported. In this study, 56 *OfGT* gene members were identified in *O. fragrans* (Additional file 2: Table S2). A phylogenetic analysis showed that the *O. fragrans* trihelix genes formed five clades (GT-1, SH4, GTγ, GT-2, and SIP1) (Fig. 1). According to previous studies, genes with few or no introns have high expression levels in plants [36], and 34% of trihelix genes in the woody plant *P. edulis* have no introns [10]. This proportion is greater in grasses (87% in *Brachypodium distachyon* and 90% in wheat) [34]. The level in *O. fragrans*, at 23%, was similar to that of other woody plants. Therefore, we speculated that the expression levels of trihelix genes may be higher in grasses than in woody plants. The specific sequence motifs present in each subfamily may confer specific functions for the trihelix protein members [37]. A MEME analysis showed that *OfGT* genes coming from the same subfamily usually have similar motif compositions (Fig. 2c); therefore, their functions may have similar. In addition, the similarities in the motif compositions and genetic structures of most *OfGT* genes in each subfamily indicated that the phylogenetic tree was reliable (Fig. 2).

The outspread of the gene family and the mechanisms of genome evolution mainly depend on gene duplication events, including tandem and fragment duplications [38]. Previous studies have shown that the *O. fragrans* has two whole-genome duplication (WGD) events [31], which could lead the expansion of the Trihelix family. In this study, most of the *OfGT* genes (78.6%) were fragment duplication genes (Fig. 4a), and there were more than in *P. trichocarpa* (50%) [11]. We suggest that the amplification of trihelix gene family members in *O. fragrans* mainly occurred through gene fragment replication events. Generally, positive selection pressure is conducive to gene amplification or functional differentiation, whereas purification selection pressure tends to increase gene conservation [39]. In addition, all the pairs of tandem repeats and fragment repeats in *O. fragrans* had Ka/ Ks < 1 (Additional file 6: Table S6), which indicates that most of the *OfGT* genes have undergone strong purification selection during the evolutionary process.

A gene expression profile is essential to analyze the functional roles of various trihelix genes [40]. In this study, the expression pattern of each *OfGT* gene in each different tissue varied (Fig. 5). The *OfGT* genes specifically expressed in tissues may have tissue-specific functions. For example, *AtGT2*, which plays a fundamental role in salt-stress responses, is highly expressed in rosette leaves [17]. In a comparison of three candidate genes (*OfGT3/42/46*), *OfGT3* was highly expressed in roots and mature leaves, whereas *OfGT42/46* were highly expressed in leaves. We speculate that the *OfGT3/42/46* genes may be involved in responses to salt stress. In addition, *OfGT47* is only expressed in roots, whereas three other genes (*OfGT19/22/43*) are only expressed in stems. These results implied that some *OfGT* genes have tissue-specific functions in *O. fragrans*, and the genes could play crucial roles in plant growth and development.

The RNA-seq data and qRT-PCR confirmed that *OfGT3/42/46* were induced by salt stress (Fig. 6a-b). In addition, *NP_001236630.1*and *NP_001236643.1* are salt tolerance genes reported in soybean, *OfGT3/42* and *OfGT46* have very high homology with *NP_001236630.1* and *NP_001236643.1*, respectively [22]. (Additional file 11: Fig. S4). We speculated that the *OfGT3/42/46* genes may play important roles in improving the salt-stress tolerance of *O. fragrans*. The subcellular localization results showed that *OfGT3/42/46* localize in the nuclei (Fig. 9), which implies that the three TFs regulate the transcriptional processes of target genes in the nuclei. Furthermore, after transient expression of *OfGT3/42/46* genes in tobacco, the MDA content was reduced compared with the control, which indicating that overexpression of *OfGT3/42/46* increased the tolerance of plants to salt stress (Fig. 10). It is worth noting that the overexpressing of *OfGT3/42/46* in the tobacco, the gene expression levels of *NbAPX* (ROS-related genes) were significantly up-regulated (Fig. 11), implying that *OfGT3/42/46* genes can reduce the oxidative damage of cell membrane by activating ROS-related genes. However, *OfGT3/42/46* showed no transcriptional activities, as assessed by a transcriptional self-activation analysis (Additional file 9: Fig. S2). In *A. thaliana*, the *AtGT4* TF interacts with the *TEM2* gene to regulate the expression of the salt-responsive gene *Cor15A* to enhance the plant’s salt tolerance [24]. Hence, we speculate that *OfGT3/42/46* might regulate downstream genes by forming complexes with other TFs.

Promoter *cis*-elements play essential roles in responses to biotic and abiotic stresses in plants [41]. In this study, many important *cis*-acting elements relevant to plant abiotic and hormonal stresses were discovered in the 56 *OfGT* genes, including CGTCA-motif, P-box, ARE and ABRE (Fig. 3). Here, some *OfGT* genes were found to play roles in responding to stresses. For example, the expression levels of *OfGT1/3/42/45/46/52* were up-regulated under salt-stress conditions (Fig. 6b), the expression levels of *OfGT12/13/15/42/45/52* changed under waterlogging stress (Fig. 7a), the expression levels of *OfGT3/13/45/46/52* were induced by drought (Fig. 7a), and the expression levels of *OfGT3/12/15/33/42/45/46* were induced by MeJA, ABA, and GA_3_ stresses (Fig. 8a). In addition, some *OfGT* genes responded to multiple abiotic and hormonal stresses in *O. fragrans*, such as, the *OfGT3/42/46* genes were up-regulated under salt-stress conditions, and they were also induced by drought, MeJA, ABA, and GA_3_ stresses (Figs. 6b, 8a). In *P. edulis*, *P. trichocarpa*, and other species, there are reports of trihelix TFs responding to salt, drought, and hormonal stresses [10, 35]. These analyses indicate that the trihelix TFs play important roles in plant adaptation to stressed environments, and some of the *OfGT* genes respond to multiple stresses in *O. fragrans*. Moreover, most *OfGT* genes’ expression patterns are similar in different stresses, and there are many instances of co-expression (Figs. 6c, 7b, and 8b). For example, *OfGT42/OfGT46* showed a co-expression pattern under salt-stress conditions (Fig. 6c), and this may play a role in improving the tolerance of *O. fragrans* to salt stress through cooperative relationships. In particular, the expression patterns of some *OfGT*s had high correlations after GA3 and MeJA treatments (Fig. 8b), which indicates that the *OfGT* genes might enhance the tolerance to GA3 and MeJA through cooperative effects. Remarkably, the integration of expression data under multiple treatments have been used to perform the co-expression analysis (Additional file 8: Fig. S1). We found that *OfGT13/OfGT46*, *OfGT3/OfGT13* and *OfGT42/OfGT15* gene pairs had higher expression coefficients in most of stress conditions, indicating these gene pairs could have synergistic effect in response to different environmental signals.

## Conclusion

In this study, 56 trihelix TF genes were identified in the *O. fragrans* genome, and they were divided into five subfamilies. The gene duplication events analysis showed that the fragment duplication events contributed to the expansion of the *OfGT* genes family in *O. fragrans*. Here, we determined that the *OfGT* genes have different expression patterns in specific tissues, and the *OfGTs* also contain a variety of *cis*-elements involved in responses to multiple abiotic and hormonal stresses. The qRT-PCR confirmed that these *OfGT* genes are induced by salt and other stresses. It is worth noting that the *OfGT* genes showed many co-expression patterns during different stress induction, which may mean that some *OfGT* gene members play cooperative roles in specific stresses. Furthermore, the decrease in MDA content and the expression levels of ROS-related genes up-regulated after the transient expression of nucleus located *OfGT3/42/46* genes in tobacco indicated that these genes could enhance the salt tolerance of tobacco. In short, our research results strengthen our understanding of the *OfGT* gene family and the salt-tolerance mechanisms of *O. fragrans.*

## Materials and methods

### Identification of the trihelix family in *O. fragrans*

The release of the genome-wide sequence data provided us an opportunity to research the members of the *OfGT* gene family [31]. The Hidden Markov Model (HMM) profiles of trihelix TFs (PF13837) were acquired from the Pfam database (http://pfam.xfam.org/) to identify putative *OfGT* gene members. Then, the online software SMART (http://smart.embl-heidelberg.de/), Search Pfam (http://pfam.xfam.org/search/), and CDD (https://www.ncbi.nlm.nih.gov/Structure/bwrpsb/bwrpsb.cgi/) were used to examine the *OfGT* domains that were conserved among these protein sequences. The isoelectric points and protein molecular weights of the *OfGT* genes were predicted using the online website ExPASy (https://web.expasy.org/compute_pi/).

### Phylogenetic analyses of the OfGT proteins

Phylogenetic trees from *Arabidopsis*, *O. sativa*, and *O. fragrans* were constructed using MEGA5.1 software to perform 1000 bootstrap replications with the NJ (neighbor-joining) method. The trihelix TF protein sequences in *Arabidopsis* and rice were downloaded from TAIR (https://www.arabidopsis.org/) and PlantTFDB (http://planttfdb.gao-lab.org/), respectively. The online WoLF PSORT site (https://wolfpsort.hgc.jp/) was customed to forecast the subcellular localizations of OfGT proteins.

### Gene structure, motif compositions, and promoter analysis of the *OfGTs* gene family

The *OfGT* gene structures were visualized using TBtool tools [42]. The online website MEME (https://meme-suite.org/meme/) was utilized to analyze the conserved motifs of OfGT proteins, the parameters related to motif repeats were set to ‘any’, the motif prediction number to 25, and the motif length to 6–200 aa [43]. The online tool PLACE [44] was used to identify the *cis*-acting elements of 2000 bp DNA sequences upstream of the *OfGT* genes.

### Chromosomal distribution and gene duplication events of *OfGT* genes

Chromosomal information for *OfGT* genes were isolated from the genomic database for *O. fragrans* [31]. TBtools tools was used to visualize the distribution of the *OfGT* genes on the chromosomes [42]. A Multiple Collinearity Scan toolkit (MCScanX) was used to analyze duplication events involving *OfGT* genes [45]. The online tool DNAsp V6 (http://www.ub.edu/dnasp/) was utilized to calculate Ks and Ka substitution rates to further analyze the evolutionary selection pattern of *OfGT* genes in *O. fragrans* [46].

### Expression profiles of *OfGT*s in salt-stressed leaves and different tissues of *O. fragrans*

We obtained the RPKM data for *OfGT* genes in the tissues roots, stems, leaves (young and mature), and flowers (initial, full-booming, and final fading flowering) from the *O. fragrans* transcriptome database [47]. The FPKM values for the *OfGT* genes in leaves were obtained from the *O. fragrans* transcriptome database under salt stress conditions (Additional file 7: Table S7). The TBtools software was used to construct heatmaps.

### Plant materials and treatments

The material for this experiment was 2-year-old cutting seedlings of *O. fragrans* ‘Rixianggui’ [48], which was planted in the experimental field of Nanjing Forestry University, Nanjing, China (32°5′N,118°48E′). First, seedlings with the same growth trend were transplanted into a pot (10 cm pot height, 10 cm inner diameter), and placed in a growth chamber for 3 weeks under the following conditions: light/dark: 16/ 8 h, day/ night temperatures: 23 °C/21 °C, light intensity: 260 μmol m^− 2^ s^− 1^, and relative humidity: 62% [49]. Then, the seedlings were exposed to abiotic stresses and hormones. Abiotic stress treatments included salt, waterlogging, and drought. The salt stress was achieved by treating with 250 mM NaCl solution containing 1/2 Hoagland’s nutrient solution. Waterlogging stress was achieved by soaking the seedlings in a container with 1/2 Hoagland’s solution. Drought stress was achieved by treating with 20% PEG6000. Hormone treatments involved spray plant leaves with MeJA (100 μM), GA_3_ (50 μM), and ABA (100 μM) [11, 34, 50]. Finally, samples were collected at 0, 6, 24, and 72 h after the abiotic stress treatment, and hormone treatment samples were collected after 0, 6, and 24 h. Three biological replicates were collected for each sample. Identification of the plant variety was made by Qibai Xiang based on reliable sources available in the literature. A voucher specimen of ‘Rixianggui’ has been deposited in the National Germplasm Bank of *Osmanthus fragrans* in Nanjing, China (31°35′N,119°09E′).

### RNA extraction and qPCR

The Plant RNA Extraction Kit V1.6 (Biofit, Chengdu, China) was utilized to extract total RNA from similar-sized leaves from the top of *O. fragrans*. The cDNA Synthesis SuperMix kit (Transgen, Beijing, China) was used to transcribe the RNA into cDNA [51]. We used the Primer 5.0 tool to design specific primers (Additional file 12: Table S8). The internal reference gene was *RAN* in *O. fragrans* [47, 49]. The selected gene expression levels were analyzed using the 2^−ΔΔCT^ method [52]. The comparative cycle threshold (Ct) values were adopted to calculate the relative expression levels of *OfGT* genes [53]. Each qRT-PCR assay provided three biological replicates and three technical replicates. SPSS and Origin2019 software were used to perform the statistical analyses.

### Subcellular localization and transcriptional activation

The GFP::pCAMBIA1300-OfGT3/42/46 fusion expression vectors were constructed, and the fusion vectors were transformed independently into *A. tumefaciens* GV3101. The fusion vectors were injected into 40-day-old growing tobacco leaves, and the fluorescence signals of green fluorescent protein were observed using an LSM710 microscope (Zeiss, Germany). BD::PGBKT7-OFGT3/42/46 vectors were constructed, and both the constructed vectors carrying the target genes and the empty vector were transformed into the AH109 yeast strain. By observing the growth of the transformed yeast strains on SD/−Trp, SD/−Trp-Ade, and SD/−Trp-Ade + X-α-gal media, the transcriptional activation activity of each target gene was determined.

### Transient transformation of *OfGT3/42/46* and measuring the malondialdehyde content

Agrobacterium infection of *N. benthamiana* leaves is the commonly used transient expression system in plants [54–56]. The fusion vector pCAMBIA1300-OfGT3/42/46 and pCAMBIA1300 (control) were introduced into *A. tumefaciens* GV3101 and then injected into 35-day-old tobacco leaves. The transiently transformed tobacco was placed in a growth chamber for cultivation (the conditions were the same as previously described). After 2 days, the plants were irrigated with 500 mM NaCl solution and then samples were collected after 12 h, with three biological replicates for each sample. RNA was extracted from the collected samples and reverse transcribed into cDNA for semi-quantitative RT-PCR analysis. Tobacco *NbL25* was used as an internal reference and the primers used in *OfGT3/42/46* were the same as those used in the qRT-PCR analysis. The determination of MDA content was modified with reference to Zhou and Leul [57]. After grinding the fresh leaves without main veins with liquid nitrogen, a 0.2 g sample was weighed and homogenized in 5 mL 5% TCA. We mixed the extract with 2 ml of 0.67% TBA. The mixture was heated at 100 °C for 30 min and then placed in an ice bath to cool. After centrifugation at 8500 r/min for 20 min, the supernatant absorbance was measured at 450, 532, and 600 nm. The qRT-PCR analysis of the ROS-related genes in the *OfGT*s and empty vector transient expression tobaccos were conducted.

## Supplementary Information

**Additional file 1.**

**Additional file 2.**

**Additional file 3.**

**Additional file 4.**

**Additional file 5.**

**Additional file 6.**

**Additional file 7.**

**Additional file 8.**

**Additional file 9.**

**Additional file 10.**

**Additional file 11.**

**Additional file 12.**

## Data Availability

For RNA-seq data, we used roots, stems, leaves (young and mature), and flowers (initial, full-booming, and final fading flowering) samples data of *Osmanthus fragrans* in NCBI Sequence Reads Archive (SRA) under the accession number SRP143423. The datasets supporting the conclusions of this article are included in the article and its additional files.

## Abbreviations

Aa: Amino acids
ABA: Abscisic acid
*At*: *Arabidopsis thaliana*
Bp: Base pair
Da: Dalton
FPKM: Fragments per kilobase of transcript per million fragments mapped
GA_3_: Gibberellic acid
GFP: Green fluorescent protein
HMM: Hidden Markov Model
Ka: Number of non-synonymous substitutions per non-synonymous site
Ka/ Ks: Nonsynonymous to synonymous substitution ratio
Ks: Number of synonymous substitutions per synonymous site
MeJA: Methyl jasmonate
MW: Molecular weight
Mya: Million years ago
*Of*: *Osmanthus fragrans*
*Os*: *Oryza sativa*
PEG: Polyethylene glycol
PI: Isoelectric point
qRT-PCR: Quantitative real-time polymerase chain reaction
RPKM: Reads per kilobase per million mapped reads
TBA: Thiobarbituric acid
TCA: Trichloroacetic acid
TFs: Transcription factors
