# Correction: Insights into the trihelix transcription factor responses to salt and other stresses in *Osmanthus fragrans*

**DOI:** 10.1186/s12864-022-08624-3

**Published:** 2022-05-19

**Authors:** Meilin Zhu, Jing Bin, Huifen Ding, Duo Pan, Qingyin Tian, Xiulian Yang, Lianggui Wang, Yuanzheng Yue

**Affiliations:** 1 Key Laboratory of Landscape Architecture, Jiangsu Province, College of Landscape Architecture, Nanjing Forestry University, Nanjing, 210037 People’s Republic of China; 2 Co-Innovation Center for Sustainable Forestry in Southern China, Nanjing Forestry University, Nanjing, 210037 People’s Republic of China

**Correction: BMC Genomics 23, 334 (2022)**

**https://doi.org/10.1186/s12864-022-08569-7**

Following publication of the original article [1], it was reported that Fig. 7 and Fig. 8 were published in reversed order. The original article [1] has been updated
