# Supplementary material for: Insights into the trihelix transcription factor responses to salt and other stresses in Osmanthus fragrans
Source: BMC Genomics. 2022 Apr 30;23:334. doi: 10.1186/s12864-022-08569-7 (PMC9055724; doi:10.1186/s12864-022-08569-7)
Supplement: Supplementary file 1 — Additional file 1. [file 12864_2022_8569_MOESM1_ESM.docx]

**Additional file 1:** **Table S1** Characteristic features of trihelix genes in *Osmanthus fragrans*

| Gene ID | ID | Chromosome | Position | Amino acid length | Isoelectric point | Relative molecular weight（kDa) |
| --- | --- | --- | --- | --- | --- | --- |
| evm.model.Contig109.151 | *Of*GT1 | Chr01 | 5077489-5078850 | 453 | 5.91 | 52.46 |
| evm.model.Contig109.150 | *Of*GT2 | Chr01 | 5091046-5094537 | 617 | 5.8 | 69.22 |
| evm.model.Contig66.152 | *Of*GT3 | Chr01 | 20808005-20810033 | 528 | 5.61 | 60.06 |
| evm.model.Contig11.362 | *Of*GT4 | Chr01 | 42982812-42984857 | 376 | 4.85 | 43.47 |
| evm.model.Contig135.55 | *Of*GT5 | Chr02 | 21836767-21842392 | 384 | 9.22 | 43.51 |
| evm.model.Contig229.88 | *Of*GT6 | Chr03 | 259244-260179 | 270 | 8.32 | 32.04 |
| evm.model.Contig342.79 | *Of*GT7 | Chr03 | 5354626-5357872 | 262 | 8.2 | 29.18 |
| evm.model.Contig342.15 | *Of*GT8 | Chr03 | 5890230-5894177 | 387 | 6.05 | 43.68 |
| evm.model.Contig186.41 | *Of*GT9 | Chr03 | 7164276-7165211 | 270 | 8.32 | 32.04 |
| evm.model.Contig300.9 | *Of*GT10 | Chr03 | 10340820-10342103 | 427 | 5.73 | 48.51 |
| evm.model.Contig350.35 | *Of*GT11 | Chr03 | 17588795-17591122 | 512 | 6.4 | 58.74 |
| evm.model.Contig350.48 | *Of*GT12 | Chr03 | 17724716-17729210 | 478 | 6.8 | 51.86 |
| evm.model.Contig53.326 | *Of*GT13 | Chr04 | 215841-216833 | 330 | 8.84 | 37.08 |
| evm.model.Contig53.102 | *Of*GT14 | Chr04 | 1746184-1747777 | 358 | 5.37 | 41.03 |
| evm.model.Contig388.40 | *Of*GT15 | Chr06 | 17172082-17174561 | 322 | 5.96 | 36.59 |
| evm.model.Contig381.1 | *Of*GT16 | Chr06 | 17521537-17525372 | 350 | 9.25 | 41.31 |
| evm.model.Contig474.21 | *Of*GT17 | Chr06 | 22339282-22340273 | 303 | 8.59 | 33.97 |
| evm.model.Contig459.7 | *Of*GT18 | Chr06 | 23032538-23033535 | 305 | 7.72 | 34.14 |
| evm.model.Contig334.8 | *Of*GT19 | Chr07 | 4197787-4200275 | 529 | 6.38 | 60.03 |
| evm.model.Contig275.28 | *Of*GT20 | Chr07 | 5253330-5254394 | 354 | 9.36 | 39.82 |
| evm.model.Contig446.37 | *Of*GT21 | Chr07 | 32861334-32865515 | 382 | 8.77 | 46.49 |
| evm.model.Contig446.28 | *Of*GT22 | Chr07 | 32973903-32976568 | 529 | 6.38 | 60.03 |
| evm.model.Contig204.66 | *Of*GT23 | Chr08 | 3915849-3921753 | 494 | 6.57 | 53.64 |
| evm.model.Contig204.78 | *Of*GT24 | Chr08 | 4082078-4084447 | 510 | 6.15 | 58.31 |
| evm.model.Contig401.5 | *Of*GT25 | Chr08 | 25117967-25118911 | 314 | 4.6 | 36.96 |
| evm.model.Contig285.8 | *Of*GT26 | Chr08 | 27121534-27121869 | 269 | 8.84 | 32.1 |
| evm.model.Contig467.25 | *Of*GT27 | Chr08 | 28319927-28321301 | 271 | 8.99 | 32.4 |
| evm.model.Contig59.200 | *Of*GT28 | Chr09 | 11982265-11986295 | 408 | 5.88 | 46.38 |
| evm.model.Contig145.77 | *Of*GT29 | Chr10 | 29498017-29498927 | 267 | 4.8 | 31.46 |
| evm.model.Contig136.99 | *Of*GT30 | Chr11 | 7456469-7457758 | 429 | 6.52 | 49.4 |
| evm.model.Contig125.92 | *Of*GT31 | Chr11 | 23917847-23918873 | 321 | 9.58 | 35.8 |
| evm.model.Contig450.25 | *Of*GT32 | Chr11 | 26771551-26773547 | 311 | 9.34 | 35.06 |
| evm.model.Contig38.195 | *Of*GT33 | Chr12 | 13092742-13095123 | 627 | 6.14 | 69.75 |
| evm.model.Contig38.194 | *Of*GT34 | Chr12 | 13108701-13110008 | 435 | 6.61 | 50.58 |
| evm.model.Contig10.407 | *Of*GT35 | Chr13 | 20419361-20421816 | 525 | 5.65 | 59.72 |
| evm.model.Contig10.397 | *Of*GT36 | Chr13 | 20553269-20554777 | 405 | 9.4 | 46.49 |
| evm.model.Contig10.319 | *Of*GT37 | Chr13 | 21384922-21386085 | 387 | 9.15 | 43.77 |
| evm.model.Contig110.29 | *Of*GT38 | Chr14 | 1687242-1688342 | 366 | 8.92 | 41.33 |
| evm.model.Contig108.22 | *Of*GT39 | Chr14 | 22839926-22842115 | 324 | 6.58 | 37.76 |
| evm.model.Contig19.261 | *Of*GT40 | Chr15 | 3553645-3556605 | 401 | 8.01 | 44.86 |
| evm.model.Contig19.266 | *Of*GT41 | Chr15 | 3571021-3572153 | 340 | 4.93 | 38.78 |
| evm.model.Contig176.19 | *Of*GT42 | Chr15 | 21590869-21592875 | 529 | 5.58 | 60.23 |
| evm.model.Contig23.45 | *Of*GT43 | Chr16 | 2218924-2221189 | 350 | 9.3 | 39.07 |
| evm.model.Contig97.59 | *Of*GT44 | Chr16 | 8901834-8902928 | 364 | 8.86 | 42.67 |
| evm.model.Contig200.86 | *Of*GT45 | Chr16 | 20720334-20721356 | 340 | 9.51 | 37.87 |
| evm.model.Contig254.65 | *Of*GT46 | Chr16 | 21536126-21537987 | 485 | 7.16 | 56.6 |
| evm.model.Contig465.23 | *Of*GT47 | Chr17 | 1594834-1595988 | 384 | 5.5 | 44.83 |
| evm.model.Contig457.20 | *Of*GT48 | Chr17 | 7395929-7399038 | 281 | 4.98 | 31.53 |
| evm.model.Contig77.121 | *Of*GT49 | Chr18 | 12693875-12696090 | 518 | 7.58 | 58.08 |
| evm.model.Contig77.118 | *Of*GT50 | Chr18 | 12721344-12722651 | 435 | 6.01 | 50.17 |
| evm.model.Contig126.84 | *Of*GT51 | Chr19 | 2549679-2553478 | 381 | 9.25 | 42.8 |
| evm.model.Contig197.60 | *Of*GT52 | Chr19 | 9972492-9975533 | 303 | 6.09 | 34.4 |
| evm.model.Contig610.12 | *Of*GT53 | Chr19 | 13908186-13909165 | 297 | 6.21 | 33.13 |
| evm.model.Contig476.43 | *Of*GT54 | Chr21 | 8295242-8298839 | 407 | 5.68 | 45.91 |
| evm.model.Contig159.114 | *Of*GT55 | Chr22 | 9517107-9518324 | 317 | 5.79 | 37.13 |
| evm.model.Contig159.113 | *Of*GT56 | Chr22 | 9529812-9532119 | 613 | 5.65 | 68.25 |
