# Supplementary material for: Insights into the trihelix transcription factor responses to salt and other stresses in Osmanthus fragrans
Source: BMC Genomics. 2022 Apr 30;23:334. doi: 10.1186/s12864-022-08569-7 (PMC9055724; doi:10.1186/s12864-022-08569-7)
Supplement: Supplementary file 2 — Additional file 2. [file 12864_2022_8569_MOESM2_ESM.docx]

**Additional file 2: Table S2.** The protein sequence of trihelix genes.
>OfGT9

MYMSEKPQQQAIDFYNKQETNQNSMIVQVPPSHHLITNGGDTAAAASSGGGEENAINDKPANPSFSYDPKSTAPKKRAETWVQEETRSLISLRREIDSSFNTSKSNKHLWDQISMKMRERGFDRSPTMCTDKWRNLLKEFKKAKQNQDSNGNGSAKMSYYKDIEELLRDRSKIEANYKSPTTPNAAAGGSKVESFLQFAEKANGRSTVSLERHSDHDGHPLAITAADAVGASGVSPWNWRETSGNGEQSHSYDGRVITVKLGDYTKRIGIDGTADAIKEAIKSAFRLRTKRAFWLEDEDNVVRTLDRDMPLGNYTLHVDEGLTIKLCLYEESDRLPVHAEDKTFYSEDDFRDFLSRRGWTCLREYNGYRNIDSMDELCPGSVYQGVN*

>OfGT54

MYMSEKPQQQAIDFYNKQETDRNSMIIQVHPSHHLINNGGDTAAASSGGGEENAINENTNNQSFSYDPKSTAPKKRAETWVLEETRALISLRREIDSSFNTSKSNKHLWDQISMKMRERGFDRSPTMCTDKWRNLLKEFKKAKQNQDSNQDSNGNGSAKMSYYKDIEEVLRERSMNEAIYRSTTPPNAAAAAAAGGSKVESFLQFPDKGLDDPGITFGPVEANGRSKVNLERRLDHDGHPLAITAADAVGASGVSPWNWRETSGNGEQGHSYDGRVITVKLGDYTKRIGIDGTADAIKEAIKSAFRLRTKRAFWLEDEDNVVRTLDRDMPLGNYTLHVDEGLTIKLCLYEESDHLPVHTEDKTFYSEDDFRDFLSRRGWTCLREFNGYRNIDSLDELCPGSVYRGIN*

>OfGT28

MYMSEKPSYSQESIEFYKQETLEDNNMIIEVTPPSHLFIDGGDTTSSGGDDTTPTPTATVTATNSNSTNPPSFYDPKMGPKKRAETWALEETRTLISLRREIDTFFNKSKSNKHLWGQISLRMREKGFDRSFSMCTDKWRNLLKEFKKSKQNHDKNGNGSAKMSYNKEIEEILRERNKNGASSYRSPTLPNNASVDSKIDTFMHFADKAGLDDTGIPFEPVEANGRSTLNLERRLDHDGLPLAITAADAVAAGSVSPWNWRETPGNAERSNSYDGRVITVKLGDYTKKIGIDGSGDAIKETIKSAFRLRTKRAFWLEDEECVVRTLDRDMPLGIYTLHVDKGLTIKVCLFEELDHLPVRTEDKTFYTEDDFRDFLSRHGWMCLREYNGYRSVDCMDELCAGVIYRGVK*

>OfGT25

MEGYPQYRHHYTVNTDTASASATATAGDSFPQWSIQETRNFLMIRGELDQNFMETKRNKLLWELISMKMKEKGYNKSSEQCKCKWKNLVTRYKGYETMEAEDMRLQFPFYNELHAIFVARMQRMVRFEAEGGSNSKKKALQDISSDEENDSNGAKKKRKIKGNANPSGFSSNTGNLIITNMKEILEKFMKQQIQMENQWIKAYEEREEERRIKEMEWRQTMEDLGNERIMMDSRWREIEEQRRIREETRAKKMDFLITTILNKLTREDV*

>OfGT27

MERYPQYRHHYTVNTDTASASATATATAGDSFPQWSIQETRNFLMIRGELDQNFMETKRNKLLWELISMKMKEKGYNKSSEQCKCKWKNLVTRYKGYETMEAEDMRLQFPFYNELHAIFVARMQRMVRFEAEGGSNSKKKALQDISSDEENDSNGAKKKRKIKGNANPSGFSSNTGNLIITNMKEILEKFMKQQIQMENQWIKAYEEREEERRIKEMEWRQTMEDLGNERIMMDSRWREIEEQRRIREETRTKKMDFLITTILNKLTREDV*

>OfGT7

MEGQHHPYTSVNIDTGGGGGADRFPQWSIQETRDFLIVRAELDPTFMETKRNKLLWEVISTRMKEKGYNRSAEQCKCKWKNLVTRFKGCETMESESMRQQFPFYNELQAIFGARMQRMLWIEAEGGGASSSKKKAAAMAQLSSDEEDDNDDSDVDQKGGSKKKRKTRSNTNPGNSSNLITSIREIMEEFMKQQMQMEMQWLKAYEAREEERRIKEMEWRKTMENLENERMMMDRRWREREEQRRIREEARAEKRDTLITALLNKLRREDM*

>OfGT6

MEGQHHPYTSVNIDTGGGGGADRFPQWSIQETRDFLIVRAELDPTFMETKRNKLLWEVISTRMKEKGYNRSAEQCKCKWKNLVTRFKGCETMESESMRQQFPFYNELQAIFGARMQRMLWIEAEGGGASSSKKKAAAMAQLSSDEEDDNDDSDVDQKGGSKKKRKTRSNTNPGNSSNLITSIREIMEEFMKQQMQMEMQWLKAYEAREEERRIKEMEWRKTMENLENERMMMDRRWREREEQRRIREEARAEKRDTLITALLNKLRREDM*

>OfGT38

MIFDIPKNVSNPSNSPRNPPPSALDHQHHPFMIPPPPSLPPFSASPSGGPGCGDDDEFPRRDERCLQWSNQETRDFIEIRGQLEGEFTSTKRNKNLWEMVVSRMREKGYRRTPYQCKCKWKNLVNRYKGQETYVVGSSQLCPFFDELHAVLSASADDTHQTQFDYEVALTRGKKRLKKVDGYQSGEKEEEDDESDADEVVKAPKRGIGREKRQRAGASEKLSKESSTNASNTDSILSNLNEMLKSFFQQQQIIDMQWRESMEKRAKERDLCEQEWRQTMEKLERDRLMMELAWREKDEKRRTREESLAEKRDDLLARLVNKLVP*

>OfGT46

MFDGMQSGDQFHQFIASPRTSFPIPLSFPLNGANPSVIPSFDPFTSHQLHLQLESSINNKVEQDYEEQTSLISTNLVLERERSMPETTMTTDLGWSNNEVLALLRIRSNIENWFSDFTWEQVSRKLGELGFKRTADKCKEKFEEETRNFNSISYNKNYRIFSGDDEFYPDDQDQELHISAEKNHAKSRRRRRRYKGEEYCRRRIRKNSIVNKIMVQQEELHNKLIEDMLKRDEQTIAREEAWRNRHTEMIKKEIEIRAEEQATARERQATIIEFLKKFTSDSCEEDQEFVTKIQDLLKVNMTCTIHSHDQTTTTQEKVEAATSSSMAFIHQKPSSKPCSSSVLLQNPNPAKSQENNQLELTPSSRKRPSKNLHCESGDSGNRWARDEVLALINLKCKLNNNDEIKDGEKGPLWERISQGMLELGYGRNAKRCKEKWENINKYFRKTKDSSKKRSLDSRTCPYFQQLSSLYSQGKLVAPDNEPENQ*

>OfGT2

MLDSSVFSENSGVDGADGSGTSGGAAAHGVAVEHRNEGGSGDGGEYFDRNSPGNRWPREETLALLKIRADMDHAFRDSILKAPLWDEVSRKLGGLGYNRSAKKCKEKFENIYKYHKRTKEGRSSRQNGKNYRFFELLEVFDNQLSVPSTPLNQVQTYLMKTTAAAPLRVNPINASQDFRVPCSNQDPDTEFMSTSTSSSEERSEGSVKRKRKLAEYFEGLMKDVLKKQEDLQNMFLESIEKYEEDWKAREEAWKVQEMARIKREQDFLAQERAVSAAKDAAVLAFLQKISQYSTTLQTPAIPFPIFEKHSDPHDNLLEKCIDKQQNGVGETSNHTDKQENSVGENATLMSSSRWPKAEVEALIMLRTDLDLKYNDNGLKGPLWEEISSAMKKLGYDRSAKRCKEKWENINKYYKRVKDSNKRRPQDSKTCPYFNMLESIYAKKSKTEHALENSSYNMQPERILFEMMGKQQHQPPPLPPSQQQHHSGTEDGESENQNQEENAEDEEDDDNGDGYQIVANIPFSLPTMG*

>OfGT42

MLASSVFLENSGVDGADGGGTSGGAASHGVAMEHRNEGGSGDGGEDIDRNSPGNRWPREETLALLKIRSDMDCAFRDAILKAPLWDEVSRKLGELGYNRSAKKCKEKFENIYKYHKRTKEGRSSRQNGKNYRFFELLEVFDNQLSVPSTPLNQVQKYLTETTATAPLRVNPLNASQDFRVVPCSNQDPDTDFMPTSTSSSEERSEGSVEKKRKLAEYYERLMKDVLKKQEDLQNKFLEAIEKYEKDRIARDEAWKVQEMARIKRERDFLAQERAISAAKDAAVLAFLQKISQHSTNLQIPEIPFSIFEKHLETNDNVLEKRIDKQENGVGETSNHTDKQENSVGENTTLMSSSRWPKAEVEALIMLRTDLDLKYNDNGPKGPLWEEISSAMKKLGYDRSAKRCKEKWENINKYYKRVKDNKKRRPQDSKTCPYFNLLESIYAKKSKTEHTSENSSYNMQPERILLEMMGQQQHQPPPPQPTQQQHQSGADDGESENQNQGDNAEDEEDDDNGDGYQIVTNIPFPLSTMG*

>OfGT50

MLGDSGGGAAAVKGNESGDEVGLGSGGFGEEDKLGIDEGERSCGVIGGGGNRWPRQETLALLKIRSDMDAAFRDSSLKGPLWEEISRKMAELGYQRNPKKCKEKFENVYKYHKRTKEGRSSKPDGKSYRFFDQLQALEHNPPPLLAPPPQVAVVPPPLVTINPPVAAHVSPLSTAPQAHTFQASRSKITTSHLSVSTSSSTSSDEDIQRRHRRKRKWMEYFERLMKDVTEKQEEMQKKFLETLEKGERDRMAREEAWRVQEMTKMNKERELLVQERSIAAARDSAVIAFLQKITGQQNLQIPTIQVPQNPSPPVAPPQQSPVPPAPPPVPLTAEATPVRIDTPKTDNGGENFILASPSRWPKAEVEALIKLRTDLDLKYQENGPKWPLWEEISSVMAKIGYNRSAKRCKEKWENINKYFKKVKECNKKRPEDSKTCPYFEQLNAIYKEKAKNESSSINPGYNISKPENPMVPLMAVPEQQWPLPLQAELQQESALDEDGDGDEDEGGGYEIVTNKQPS*

>OfGT55

MMLGVSGIISEGGGAAPEGHESGGASGGSSEVGGGATSIPGGFSEEDKAAGGARMEESERSVGGNRWPRQETMALIKIRSDMAVAFRDSSLKSPLWEEVSRKMAELGFERTAKKCKEKFENLYKYHKRTKEGRAAKQDGKTYRFFDQLEALESNTPQPVLAQPQPLSMVAPPPIPNPVNVSQNAAAAAVQGANNSTSSLFQQLQPPPLHPALPPPTMNTTSTTITYPPQSHHFQPSNMVFSNSSSSTSSDEDIQRRHGRKRKWKDFFERLMKDVIDKQEELQKKFLETLEKRERDRMAKEEAWRVQEMTRLNREHDLLVAERSIAAAKDAAVIQFLQKITEQQNLQIPISNANPPQMPMSMPMPMQVQAPENTPPPVPPPQPAAPPQQTPALAPAPAPTLAQVVVTPTKVLDTPRTDNNGGENFMPASSSRWPKAEVQALIDLRTSLDLKYQENGPKGPLWEEISAAMARLGYNRNAKRCKEKWENINKYFKKVKESDKRRPEDSKTCPYFHQLDALYRKRAKTDSSSNPGFLMKPDQNPMMPIMARPEQQWPLPQGQHQDSALDDRGTENVDENDDSEDEDDDEDEDEGANFEIVTNKQQSSTPNTNTNTIE*

>OfGT34

MMLGVSGIMNSSGEGGGAATEGNESGGASGGSSEVGGGATSIPGGFSEEDKAATAARMEESERSVSGNRWPRQETMALIKIRSDMAVAFRDSSLKGPLWEEVSRKMAELGFERTSKKCKEKFENLYKYHKRTKEGRAAKPDGKTYRFFDQLEALESNTPQPVLTRPQPLSMVAPPPMSGNTNPPMLAVSMSSPKPVNVAQNPVSAAAQCSNNSTLFQPLQPPPPQPALPPPTMNTTTITTYPPQSHIFQPSNVVFSNSSSSSTSSDEDIQRRRGRKRKWKDFIERLMKDVIDKQEERQKKFLETLEKRERDRMAKEEAWRVQEMTRLNREHDLLVQERSIAAAKDAAVIQFLQKITEQQNLQIPISNANAPQIPMPMPMPMQVQVPENPPPPQQTTAPSPSPAPPATRSPAPTLAPVVVTPTKILDTPTTDNNGGENCIPASSSRWPKAEVQALIDLRTSLDLKYQENGPKGPLWEEISAAMAKLGYNRNPKRCKEKWENINKYFKKVKESNKKRPEDSKTCPYFHQLDALYRERAKTDASSNSGFIMKPDQNPMMPIMARPEQQWPLPQGQYLHQHQDSAPDYHGSENVDENDDDEDEDEDEGGNYEIVTNKQQSSTTNTNTNTIE*

>OfGT37

MDDETGMADLRQYMNRRPFFPPISPGTGLSSDHHPGLTQPQQYDVFMIPHGLQPAFRSDSTTSNTGGGSSSSAGGFGGFEMEAGGLNGFGDGGTGRWPRQETLTLLEIRSRLDPKFKEGNQKGPLWDEVSRIMAEEHGYQRSGKKCREKFENLYKYYKKTKEGKAGRQDGKHYRFFRQLEALYGEISNTVPVSETHLAGSTFRNNISSNNILTPNQQEVYQAPKLSDTSLSLSNSSDFDTTSSDGGDVNGRINDDSSGNRQKKRGNRRWKGKIKDFIDAQMRKLVDKQESWMEKMMRTIEDKEQERMIREDEWRKQDADRIEKEHKFWTSERAYIEARYAALMEAIRNVAGKELKASPSDELMAVKTRGLSENENDDEMGDTIWPECEITRLIQLRTNLESRFQQVGFSEEVLWEGIATKMACFGYDRNALVCKEKWDSVNNYVIKCNKKRKESPKPYCYYCQTNVSICNNHGGAFFDNTSGLGPEASPSNSNTGNAMNDSCFRYFMEDDHNTLENFGLKLNKLG*

>OfGT21

MVMDDETGMADLRQYINRRPFFPPIPPATDLLSGHHPGLTQPQQQYDMFMIPPGLQPAFRSDSTTSNTTAGGGSSSSAGGFGGFELEGGGLNGCGDGGTGRWPRQETLTLLEIRSRLDPKFKEANQKGPLWDEVSRIMSEEHGYQRSGKKCREKFENLYKYYKKTKEGKAGRQDGKHYRFFRQLEALYGEISNTVPVSETHLAGSTFRYNMSTNNALTPNQQEAYQAPKLSDTSLSLSNSSDFDTTSSDGGDVNGGANDDSSGNRQKKRGKRRWKAKIKDFIDAQMRKLMDKQEAWMEKMMRTIEDKEQERIIREEEWRKQDADRIEKEHKFWTNERGWIEARYAALMEAIRNVTGKELKASPSDELMAAETRSLSENQNNDEKGDIWPECEITRLIQLRTNLDSRFQQGGISEEVLWEGIATKMACFGYDRSALVCKEKWDSVNNYVIKCNKKRKENPKPCLYYCQNNESISNNHGGAFCDNTSGIGHETSPSNSNTGNAMNDSCFRYFIGDDHNMWENYGLKVNKLG*

>OfGT20

MVMDDETGMADLRQYINRRPFFPPIPPATDLLSGHHPGLTQPQQQYDMFMIPPGLQPAFRSDSTTSNTTAGGGSSSSAGGFGGFELEGGGLNGCGDGGTGRWPRQETLTLLEIRSRLDPKFKEANQKGPLWDEVSRIMSEEHGYQRSGKKCREKFENLYKYYKKTKEGKAGRQDGKHYRFFRQLEALYGEISNTVPVSETHLAGSTFRYNMSTNNALTPNQQEAYQAPKLSDTSLSLSNSSDFDTTSSDGGDVNGGANDDSSGNRQKKRGKRRWKAKIKDFIDAQMRKLMDKQEAWMEKMMRTIEDKEQERIIREEEWRKQDADRIEKEHKFWTNERGWIEARYAALMEAIRNVTGKELKASPSDELMAAETRSLSENQNNDEKGDIWPECEITRLIQLRTNLDSRFQQGGISEEVLWEGIATKMACFGYDRSALVCKEKWDSVNNYVIKCNKKRKENPKPCLYYCQNNESISNNHGGAFCDNTSGIGHETSPSNSNTGNAMNDSCFRYFIGDDHNMWENYGLKVNKLG*

>OfGT24

MEDQYEMGDLRQCINGRALFPPISQPPDLLSSHSGFTPAQHYEMLMVPRGLRQEFLSDSTTSASFNLSKSASTGGGSSIGAGVGGFDMEAVGLNGGSGGDMGTGRWPRQETLTLLEIRSRLDPKFKEANQKGPLWDEVSRIMSEEHGYQRSGKKCREKFENLYKYYKKTKEGKAGRQDGKHYRFFRQLEALYGETNNGALASETHVVGSSFGYKTPNAVPNHDTYPAPKISDYSLSLSNSSDFDTTSSDDSDLHEGIDDNSTNKRKKSRGKRCWKAKIRDFIDAQMRKMMDKQEAWMEKMMIAIEHKEQERILREKEWRKQDAERIEREQKFWANERAWIEGRDASLMEALQKLNGKELMAAAEIQSLSANPNNDTVKADNIWPDREITRLFQLRASMEERFQQGGVSEDVIWEEIATKMACFGHDRSGLTCKEKWVCVNNYLLECNNKRRENSKGCSYYQNNNESISNERDADHTSRSNDNGMNDFCFRYFMGDTDNTWENYALKLNKG*

>OfGT11

MEDQYLMADLRQCINGRALFPPISHPPDLLSGHSGFTPAQHYEMMMVPRGLHQEFLSDSTTSASFNISKSASTGGASSIGAGVGGFDMEAVGLNGGRGGDMGTGRWPRQETLTLLEIRSRLDPKFKEANQKGPLWDEVSRIMSEENGYQRSGKKCREKFENLHKYYKKTKEGKAGRQDGKHYRFFRQLEALYGETNNGASASETHVHRSSFGYKTPNAVPNQETYPAPKISDYSLSLSNSSDFDTTSSDDIDLHEGIDDSSTNKRKKSRGKRCWKAKIRDFIDTQMRKLMDKQEVWMEKMMETIEHKEQERILREQEWRKQDAERVEREQKFWANERALIEGRDAALMEALQKLNGKELMAAAGKDHNYDRSETITNTVKGDNIWPDREITRLFQLRTSMEDRLLQGGVSEEVIWEEIATKMACFGHDRSGLTCKEKWVSVNNYLLECNNKRRENPKGCSYYQNNIESICNEREADHTSRSNDNGMNDFCFRYFMGDADNIWENYALKLNKG*

>OfGT3

MEKGRNFLALTPLSSLVRMEDGPRERAGSLDVWVGCGGTMTVIGKKRGLGLGLELLFMMLGDSDGGAAPVKGNESGDVGLGSGGFGEEDKLEIEEGDRSGGGGGNRWPRQETLALLKIRSDMDVAFRDSNLKGPLWEEVSRKLAELGYQRNPKKCKEKFENVYKYHKRTKEGRSSKPDGKTYKFFDQLQALEHNPPPLLAPPPLGTTNPPIAAYVSPISTVPQGTNNPTNFSFPPLQPPTMHTTNPPQTHTFPPSRPNISTSHLSISTSSSTSSDEDIQRRQKRKRKWMDYFERLMKDVIQKQEELQKKFLETLEKRERDRIAREEAWRLEEMAKMNKEHELLVQERSIAAAKDSAVIAFLQKITGQQNLQIPTVQVPHNPSPPVAPPQPPPAPPPVPVTAEATPERIDTPKTDYGGENLPLASSSRWPKAEVQALIKLRTDLDLKYQDNGPKGPLWEEVSAAMAKIGYNRNAKRCKEKWENINKYYKKVKESNKKRPEDSKTCPYFEQLDAIYKEKAKNESASINSGYTLPKPENPIVPLMAVPEQQWPLPLRPEHQQESALDHEHENIDRNDRDNDQDDDDDDGDYRDDEDEDGGGGYEIVTNKPQSSVTSTAFE*

>OfGT48

MQDHDQTFSMADYDRRERRKSASDEDEPSFTEENADGSCDPSRGKKASPWHRVKWTDAMVRLLITSVSYISEEAAAEYGGGGRRKYASLQKKGKWKSVSKVMAERGHFVSPQQCEDKFNDLNKRYKRLNEILGRGTSCEVVENPPLLDVMDHISEKAKEEVRKILSSKHLHYEEMCSYHNGNRLHLPPDPELQRSLHLALGSRDDPDDSDVKRQSKDYNEEVDEEDELDDHDESEDNLVKSGDHMAYGMPGSSTKRIKQYQNHEDFSFGNSLNLLDRNRTSNFQPVNVDADVDTDAKQGSPEGMKTNRLQKQWINHQALQLEEQRLHLQEQMLDLEKDRFKWQRFCQKKDREMEMARMELERMKLENERMALELRRKEIGIDNS*

>OfGT56

MMQGNGSYLEECKIATRLFDYSKCQSVSDKDEQTCTEENDPSKGKKASPWQRVKWTDAMVRLLITSVSYISEEAASEYGGGVRRKCANLQIKGKWKLVSKVMAQMGHFVSPQQCEDKFNDLNNSYKRLNEILGRGTSCEVVENPMLLDMMDHISAKAKEEVRKILSSKHLHCEEMCSYHHGNRHTQDEDEVDEEIELDGRDESDENPILFGDHRAYKTSQENADADADINQVPPESMIDNTLQKQWMNHRTLQLEEKRLHIQARMLELEKERFKWQRFCRKKDRKLEMKRLEIKKMKLENERMGFELRQEEIGFDNS*

>OfGT44

MEANLSLSGDMVQVNHQQAPYTWQVPDAFPFQVGSMLKFDQPMPLLEGNIDERLNNIQGNSRWMNILGTEKWVPVWQRIKWTDEMVMLLITAVSYVDEDALSTANNSERRKQFIPLVKGKWRAISNAMVERGYIVSPQQCQDKFNVLNKKYKRLNEMLGRNNVCEVVANPVVMERMNISDEMKEKAKKILTCKQLFYREMYSYHYRNRIFLLHDEALQNSLRFALKVMYESKDVSQASAKRQKLQKEHGVVNIDGSKNYVDCMGSDPQSPKDIVNANFVLPKGVEGEQEQDQRLISRSLRLKKKMLQIQGEMLELEKKKFDWLKLSQEADRELIKMKQENELMRLENRRLAFELTCLEMDADRK*

>OfGT4

MEGNLSSGNKLQGNGSYGGFNLQGPVRVHHHHQQQQQQHPLALHQQHSSHPQQGSLMARPTIHENFSLTIGNAQDSEQTISFEDYSKGERVKPASDEDEPSFTEDATDGRNDPTGGKRASRWQRVKWTDSMVRLLITAVSYISEEAATEYGCGGGRRKYANLHKKGKWKSVSKVMAERGHFVSPQQCEDKFNDLNKRYKRLNEILGRGTSCEVVENPTLLDMMDHISEKAKEEVRKILSSKHLHYEEMCSYHNGNRLHLPPDPELQRSLRLALGSGDDHDENEVKRHLQDDNDEVDQEAELDGHDEYEDNHVFCGDRRVQEIPGNSTKRLKQCQNHEEFSFGSSFNSLDCNKTFNFQPEIIEADVSQVLPESMKANMSQKQWLNHRSLQLEEQRLHIEAQMLELEKERFKWQRFCKKKDRELEMTKMEIERMKLENEHMSLELRRREMGIDSS*

>OfGT8

MESNDMPGGMYSSLNPGMLGLEMSLQHRLPPPPHSTVGCGGDYHSETQQSGKQGFSHAAKGRNPILTFSDDDERGFTAENIADDGKKKMSPWQRMKWTDNMVRLLIMAVYYIGDEVGSEGNDIKKGGTVLQKKGKWKSVSRAMVEKGFYVSPQQCEDKFNDLNKRYKRVNDIVGKGSACRVVENQSLLDTMDNLSMKMKEEVKKLLNSKHLFFREMCAYHNSGGHGTHSGGGGGGGGAQDSLAEPSQSQQQMCLHSSAKNKGSKMEKGISIDEEYDEEDGDEDDDDDEEDEYEDDDDPDEQSSRKRRKKGLFHSPMIQQFSTELTNMLQDGTRSPLEKRQWMERRSMQLEEQRVGFQSQAFEIEKQRLKWLKFSAKKEREMEREKLSKERLKLENARMILLIRQKEIELLDLQSSTNKRSDPSSITG*

>OfGT31

MDRKSSASRNLKPRKSYGFLDLQAPMQVNRQQNRCQGFMATSSAQVQDVFPIPNRNMRRFDAPMPLVDCHGKPEVTNNSCDEDNSYLVGQHAVEHNDGVREEKELPWQRVKWTDQMVKLLITAVSYIGEDPVSDAGDAGGRRLSPLLPKKGKWKAISKVMAEKGYHVSPQQCEDKFNDLNKRYKKLNDILGRGTSCKVVENSTLLDLMDLSDKAKSAVKKILGSKQLFYQEMCSYHNRNRMYIPHDQAIQRSLQLALRSSDDQESHETRAYITDSTNKEEVNETSDGVNDDTRDKHDLSKDLTLRVPAKRKKPGGEHEDMDSSYIMNFHTAVKRSCPHYQPSANPVFGEGSDGESLHNQWFMSRTLQLEEQKLQIQAEMLELEKQHIRWLRSSEVEDRELEKMRLENEYLKLENEQLAFEIKCREMGAI*

>OfGT36

MESNAMPGGIYSSVSPGTLGLEMSLNHNIPPQNPNHQQQQQRRHNPMAANGHREGEYRPQTQQSVKQGHPYVGKAKNQTLTLSDDVEHGPENTSDDGNLRMSPWHRVKWNDNMVRLLIMIVFYIGDEVGSEANDPANKKGGVILQRKGKWKSVSRAMMEKGFYVSPQQCEDKFNDLNKRYKRVNDILGKGTACQVVENQSLLDSMDHISPKMKEEVKKLLNSKHLFFREMCAYHNSCGHGPHGSGSNGSGAVANQHSPAEVAADPSHTQYQSQQQRSSMKRQRKNVFDSPFIQQLNMELMNILQDGTKSPSEKRQWVKERLMHLEDQRVGIECQAFELEKQRLKWLKFSTKKKREMEREKLANERLRLQNERMALLIRQKELELLGHHHYQLQSSNKMGDPSITR*

>OfGT33

MEGNFSSGSMMQGNGSYVDFDLQGPMRVHHGQRHAIALHQQHHQPHAGQGSLTVLPTIHANFPLTIGRMQDREKTVSQADYSERERGKSASDEDEQTCMEENDLSKGKKASPWHRVKWTDTMVRLLITSVSYISEEAATEYGGGVRRKCANLQKKGKWKLVSKVMAQRGHFVSPQQCEDKFNDLNKRYKRLNEILGRGTSCEVVENPMLLDMMDHISAKAKEEVLKILSSKHLHYEEMCSYHNGNRLHLPPDPELQRSLRLALRSRDNEDEDVKRHTQDDDEEIELDGRDESEENPVLFGDHRAYKTSGSSAKKIKQCHNHEEFSFGNSLDCNRTSNIQQENTGADINQVQPDGMKENMLQKQWINHRTLQLEEKRLHIQAQMLELEKERFKWQRFCQKKDRELEMTRLEIKKMKLENERMAFELKQKEIGFDNR*

>OfGT22

MESNVMPGGMYSSVNPGILGLEMSLNHKIPPQNPDQMQQQHQHNSMATYGHREGEYHPQTQQSVKPWRPFSGKAKNQSLTLSDDDEPGPENVSDDGNMRMSPWQRVKWNDNMVRLLIMIVFYIGDEVGSEGNDPAKKKGGGVLQKKGKWKSVSRAMMEKGFYVSPQQCEDKFNDLNKRYKRVNDILGKGTACRVVENQSLLDSMDHLSPKMKEEVKKLLNSKHLFFREMCAYHNSCGHGSGASGSGVGGNQHSPAEVATELSHTQFQSQQQRFTIPELDLFEQIISVFISVKIIVICFSNITSCIPTPSLHSVMENRVEGEAEAKTAFRLIGWRLGVGVGVYSSRIHWLRASGAVVFRQPDFVRRQSPLHHDFNLGKFGFFL*

>OfGT49

MVEGNLSSGNMLQGNSSCGGFNLQGPVRVHHQQQQHNSHPRQGSSMVRPKSHENFSLTIGNTQDCEPTIFFTDYSKEESVNPASEEDNPGFTEDPSGGKRASQWQRVKWADSMVRLLITAVSYISEEAAAEYGCSGGRRKYANLHKKRKWKSVSKVMAERGHFVSPQQCEDKFNDLNKRYKRLNEILGRGTSCDVVENPTLLEMMDHISEKAKEEVQKILSSKHLHYEEMCSYHNGNRLHLPPDPDLQHSLRLALRSGDDHDENDVNMHLQDDNVEVDLEAESDGIDEYEENHGFHGGLRVRGIPGNSTKRLKQCQGGHEEFSFRSSLNSLDYKKINFQPEIPDADVTQAPLEGMKANTSQRQLLNHRALQLEEQRLHIEAQMLELEKERFKWQRFCQKKDRELEMPKMEIERMKLENEQMRLELRRREMGIDRS*

>OfGT53

MANEPREYRKGNWTVAETMVLIEAKKMDDERRLMIRYGGESSSSGKPGELRWKRVEDYCWKNGCFRSQNQCNDKWDNLMRDFKKVREYQRRVAGGDGGGEKSYWKMDKNERKEHSLPSNMLPQIFDALVEVVERNGPAEMVGGGCVGGVIVPAAEAQTMLLPLQPPAQQPTRPFAEPSFPTVDSDTSEHSESPAKRRRSRERGGDSGAGAVGGSSLQEVCSAISQSGSIVAEAIQSREEREERRHREVLSLHERSLQIEESNAEINREGINGIVDAINKLANSILALASQKNQPESK*

>OfGT18

MANEPREYRKGNWTVEETMVLIEAKKMDDERRLIIRHGGESSSAHERGKLGELRWKRVEDYCWKNGCFRSQNQCNDKWDNLMRDFKKVREYQRRVEAERGGGEEKSYWKMEKSERKERSLPSNMLPQIFDALVEVVERKVVVGGVGGGIGTSNVYPIQAAAAQTMPLPLPPPAQPPTRPFAEPSLPTADSDTSGHSDSPAKRRRRRREGGGEGGAAASGSSLQEVCSAISQSGSTIAEAIHSYEEREERRHTEVLSLHERRLQIEESNAQINRQGINGLVDAINKLANSILALASHKNQPDSK*

>OfGT17

MANEPREYRKGNWTVEETMVLIEAKKMDDERRLIIRHGGESSSAHERGKLGELRWKRVEDYCWKNGCFRSQNQCNDKWDNLMRDFKKVREYQRRVEAERGGGEEKSYWKMEKSERKEHSLPSNMLPQIFDALVEVVERKVVVGGVGGGIGTSNVYPIQAAAAQTMPLPLPPPAQPPTRPFAEPSLPTADSDTSGHSDSPAKRRRRRREGGGEGEGGAAASGSSLQEVCSAISQSGSTIAEAIHSYEEREERRHTEVLSLHERRLQIEESNAQINRQGINGLVDAINKLANSILALASHKNQPDSK*

>OfGT47

MEEQIGGSRRTRSQAAPDWTVQESAILINEINAVESEWGGTLPSFQKWQQIVENCNALEVNRTLNQCKRKWDALLNEYKRLKNEGDGGGANGSLDIEVFRAIDWCVKAKGKKSDSHGTAAETEVETVAELQSVMDTDPDSDPEAQGPPTTFVLETGIKKQGQRMKRQKRKIQRLNPWGYVTSGKTMLEQSSRNEKMYSSADEESNVETKEETLAQILRENAMLANAILEGNLADDVDYRLADLKNTDAVKIDFARHQGDKLIDCFGKISDTLNQLCDLIRQ*

>OfGT10

MSLEQLSLAPGPADGDTDGRQQSADDASKVARLPRWTRQEILVLIQGKRVAENRVRRGRASGLAFGSAQVEPKWASVSSYCKRHGVNRGPVQCRKRWSNLAGDFKKIKEWELKIREETESFWVMRNDLRRERKLPGFFDREVYDILDGGEEEGASGLALAPSVVPAGDEGGKEVEAEALFDSGRSAAADDGLFSDFEQSGQEEVGEIPGRVKETPGERYTRSDSHFSCRETASVISSRTSCSSLWPTLRLIRCSRNSKCRRI*

>OfGT13

MASEPNGVHENGTSSTCVENGQPLSIERTDDKNKIVRHPRWTRQETLFLIEGKKVAEDRGLKGRRSSSIFGSDQYEPKWDSVSSYCRQRGVNRGPVQCRKRWSNLVCDFRKIKMWESQVKGVNESFWTMRSDLRRDRRLPGFFDQEVYDVLDGKTFTDDAYRLALVPVSPDEKNGYGIDGVEAEDEDEMVEDEEAEVVFNGNYHSIPEDGLFPEFEQAAQDEIGESRGNEKATKDDRTKPIPSPVPISEKKYQPYHEAYSNRGTERGKRPQPNFETGSTMFQEGVKRRRFSSDGCQILDETDRLIRVLQSNASLLNAQLEAQNMNRQVDREQRNDQHNSLVIALNKITDALAKIADKL*

>OfGT12

MASCDDDFSLLDDHQNPDSTQPSATIQTPPFSVNPYDQDSDPFDSEPDPSSRSGKRAAERDEISNGVSKRTRLHSASSSAGDYRKDREEWSDTAIACLLEAYTDKFVQLNRGNLRGRDWEEVAATVSERCDKQSKSVEQCKNKVDNLKKRYKLERHRLSNGGLSASHWPWFQQMEQIVGNSIAAKASADEEKNSVSGGSSICTRQPKRHATATATATATASPGGQIMAMKLKPSANLRWRRVVFKISGAALAGNASNNIDSKVALLVAREISIACRYGVQVAVVVGGRNFFCGDTWVTSTGLDRCTAYQIGMMATVMNSILLQSALEKLGVHTRVQSAFSMPEVAEPYSRQRAIRHLEKGRVVIFGGIGAGTGNPLFSTDTAAALRASEIHADAVMKGTNMDGVFVCDPRNNNIAAEHISFRDLASRGASPMDMMAMTFCEENGIPVVIFNLHEPGNISRALCGEQVGTLIDQTGPIS*

>OfGT23

MASCDDDFSLLDDHRNPDPTQPSTNTQTPPFTVNPYDQDSDPFDSEPDPSSRSGKRATELDDINNGVSKRTKLPSASSSAGEYRKDREEWSETAISCLLEAYTDKFMQLNRGNLRGKDWEEVAVTVGERCDKQSKSVEQCKNKVDNLKKRYKLERHRVSNGGVSVSHWPWFQQMELIVGNSMATKAAADEEKNSVSGGSSIATRQPKRHATATASPGSQIVNVKSKPSTNLRWRRVVFKISGAALAGTAPNSIDPKVAMLVAREISIACHYGVEVAIVVGGRNFFCGDTWVTSTGLDRCTAYQIGMMATVMNSILLQSALEKLGVQTRMQSAFSMPEVAEPYSRQRAIRHLEKGRVVIFGGIGAGTGNPLFSTDTAAALRASEIHADAVLKGANMDGVFVCDPRSNNIAAEHISFRDLASRGALPMDMMAMTYCEENGIPVIIFNLHELGNISRALCGEQVGTLIDQTGPIAVVPDTLLWKHGTGLAGRGGVNL*

>OfGT43

MFQVLSSSWQLNVISFVVARGEMDREIKNGNSSSFLSINNNNEESPRKPQGGGGNSDRLKRDEWSEGAVSSLLEAYEAKWTLRNRAKLKGQDWEDVAKHVSSRANSTKSLKTQTQCKNKIESMKKRYRSESAAADASSWPLYPRLDLLLRGNAPPPLSSSPQTCHVPTLMFLESSHPQELPQPTPTVPPPPLPPAPTMVLQPGNLVQNSHGSNGLDRLAKEDGVADKLSDHESDKNRMETDSSTPGPYSGQEKLKSRNLKMKMGRRTKRRGEERDIAESIRWLAEVVMRSEQSRMETMRELERIRAEADAKRGEMDLKMTEIIANTQLEIAKLFAGIGRGVDSSLNIGRS*

>OfGT14

MEDDDEIQSHPSPASNGRITVTVAAAPPVTPQNTLTLALPIQQQKIGGTGGGREDCWSEGATAVLIDAWGERYIELSRGNLKQKHWKDVADIVSSREDYMKTPKTDVQCKNRIDTVKKKYKTEKAKIAAGGGPSKWRFYEKMDELIGPTAKMNSGNANVQSGSQRVPMGIPVGTRSGSVSQFRRQPKAQFRKRPPVDSDEESEAEQEPSADSSDGLPPESYERARVERETMMVNASASRNRKMNEETAVVNNNETNWGNSVRELTRAILKFGESYEQAEKSKMQQLVEMEKQRMKFMKEMELQRMQLIMKTQMELSQLKNKRNGNTDSSN*

>OfGT39

MASPPSSPPLLASSPQAPLPLPSPPNDTHSEDSPGPSSSASPKRVPLSLPGPESVLALPSPPKKTPPLPWNHQETVNLIRAYQEKWYSLKKGQLKASQWEEVAVTVAARCGYDEPSKTSTQCRHKIEKLRKRYRAERLKPFPNSWQYFELMDQMERGPLPLNARPVAVVKSPDTTNNNSSNGNNHINNYSSVYSGAADCDYNNSDDSGEEWNASYAVEAKKNKLKSINNLVRGEVGVKSGVRNGSKLGVERSFNEGSDRVLRALRNPMNGKRTRYNYEDSEDDDEEEERMQVKEEREERGNGGRELAGEIRGFVEKFMKMENKKIEMMRDTQRCRMEMEKKRMDMILEAQMKIIDTIGRAFGAHKK*

>OfGT26

MRNSIPHSYSTQKGQNEIEFDDNAFDEDEENPYQNVAENGQLYNYEESKRHPKKRKLESFVSNYEFVPQKGIWNEEKSFVLLEVWGERYMELGRRSLRTEDWVEVTEKVFEMNGVERTEMECRSQLDVLKNKYKKERVKVENGHASKWAFFKKMDVLLNMRTRGHCGLGCGIDSGEYVFMNPRVYLDRSNLLDEMRDSPGQSDEEDGGEEGLGDKEESAESAKLLTESIQRFGEIYEKIEDDKRKQMMELEKMRRDFQRELELQKKQIVEHAQAEIAKVRDMDDDDDDDDDDDKDNDEDDDNHNTDVSGDNLRG*

>OfGT51

MSRSSTASPPLSTTTSPNRSPSLHHPTDSMTDDENDGVPPSPSPPVTTKTESSPEAPKPECPTSARTPAFPVREDCWSEEATRTLIEAWGSQYLELNRGNLRQKHWQEVADAVNALHAHTKKLHRTDVQCKNRVDTVKKKYKIEKTKVVQSNGQFASTWPHFDSLDSLIGDTFSKANSIPSNGHKKKLRRRVTLSPEVSPSPPPTKTTMTQFRKSSPDSISPLPKVPWSVPVGPRSKRPGNGNLTERNFSVMAAAAAAVEAKEEDEDEDEDETLLRQPPVPGRKRRRPVEEGDGLVGEGYRRLAKAITRLGEIYEKVEAAKQQQMVELEKQRMQFAKDLEIQRMKIFMDSQVQVEKLKRLKNNSRNCTVWQAPTDHFGNYH*

>OfGT5

MSRSSTASPTLSTTTSPNHSPSLHHPDASMSDDENDGAPLSHPLPVTTKTESLSETPKSEGHTSTRTPTFPVREDCWSEEATRTLIEAWGSHYLGLNRGNLRQKHWQEVADAVNALHAHTKKLHRTDVQCKNRVDTVKKKYKIEKTKVVQSNGRYASTWPHFDSLDSLIGDTFSKANSVPSNGHKKNLRRRVSLSPEVSNSPPPAKTKMTHCRKRSPESITHLPKVPWSVPVGPRSKRPGNGNLTERNFSVMAAAAAAVEAKEGEEEEDEENEDETWLRQLPVVERKKRRLIEEGDGSVGKDYRRLANAILRLGDIYEKVESAKQRQLVELEKERMQFAKDLEIQRMKLFMDSQVQVEKLKRMKNHSRNCTVWQAPTDHFGNYQ*

>OfGT15

MCRAVRSTTYYRYDVDRDEEETSESTTLHQPPAAPNQIVAPMETRRQHILPAPCHRCREHLRAHTQLQPRHRRRDLRRTVPRGLWTEEATSTLVDAWGRRYLELNRGNLRQKDWQDVADAVNARHGHTKKTHRTDVQCKNRIDTLKKKYKIEKPRLLTAEIYIIAYPVGVSIDRRRYLCRRLRWLFRYRFVSCHLRCFLRLFCLRSVPFPSPSPPPPIVDESYFRRNYSAMAAAAAAAEDAPDAEDEGDSGAREVEEGDEGMWRLAKAIERFGEIYERVESMKQRQMMELEKQRMQFSKELEVQRMQLFMDTQVQLEKIKQAKQSGSSDGMGLSFCMLDFWEVQHGLFTW*

>OfGT41

MDDTREDDRYPPNSYGINDQEFPVQNATYAGNVGHEYVEDDDNDVDDEDMEYEDDDEDDGGGSSRHPKKRKLKSLLSSYEFAPRVPAPSSATPAVPKPSYGGRNPHTDWTESETVILLDAWGDRFLKHGRKSLRSEEWQEVSKKVSQESKIERTDTQCRNRLDTLKKKYKKEKMKLGESWGSTSKWVYFKKMDMLLSSGPQQAGLSCGVDSGEYTFMNPKVYLNSSNGMDEMRDSPGNSESTKSEEGDPEGFPPKKTRNEMGNGASLKLLADSIQKFSDIYEKIENSKRQQMLELEKMRMDFHRDLELQKRQILERAQAEIVKIRQGDEDNDVSAENVSG*

>OfGT29

MEFEDSAFDDDEETTYQNIAENGQLYNHEESKRHPKKRKLESFVSNYEFVPQRGMWSEEENFVLLEVWGERYMELGRRSLRTEDWVEVAEKVLEMSGVERTEMECRSQLDVLKNKYKKERVKVESGQASKWVFFKKMDVLLNLRTRGHCGLGCGIDSGEYVFMNPRVYLDRSNVLDEMRDSPGQSDEEDDEEEGLGDEEEGAESAKLLAESIQRFGQIYEKIEDSKRKQMMELEKMRRDFQRELELQKKQIVEHAQAEIAKVTLTLP*

>OfGT32

MDREAKNENSSSFLSINNNKEESPRKPQGGIGGGNSDRLKRDEWSEGAVSILLEAYESKWTLRNRAKLKGQDWEDVAKHVSSRVNSSKSLKTQTQCKNKIESMKKRYRSESATADSSTWPLYPRLDHLLRGKPPPLLSSPQNCHGSTLMFLESSQPQELPQPPLAAPPLPPAPTSVLQPENPVQNSHGSNGHDRLAKEDGVANKLSDHDSDKNLMETDSSTHLSYRRKEKRKSKNLKMKRRGEETDIAESIRWLAEVVLRSEQARMETMRELERIRAEADAKRGEIDLKRTEIIANTQLEIAKIFAGIGRS*

>OfGT45

MASTLSPSSSSPYNDHIPTATLALPSIASNSASRRLPPPCWSHDETVALIDAYRDKWYSLRRGNLRASHWQEVADEIADRCSSSIPKTAVQCRHKMEKLRKRYRSEIQRAAPHGGPRRISSSWVHFQSMHSMEKGPNTSPSYSDDEPQGEEDHKNSITRINDAHNNNYSHQKGNFSNQGLMGNGTTSGFRIKIPGKASAGYSAPKVYGKFNEMGGQNPKNPHPKINSSTSGYGVSGKFSRDGFMGRDEIGKGIGDIAKKRKGDNGMDEVVAAIETLGEGFVRMEKVKMDMAREVEQMRMQMELKRTEMILESQQRIVEAFANAISDRKPKKAKKMSMPDC*

>OfGT30

MATIFSLASPSANDDHIPTATLAVPSIAISASASRRLPPPCWSADETVALIDAYKDKWYSLRRGNLRAFHWQEVANDVAYRCPSSPAKTSVQCRHKMEKLRKRYRAEIQRAAAFGGSSRFSSSWVHFQRMHSMEKGTNSTPPSSDDGLPEEDHKNSIKRINEVYNNNGYSNQKASFGNQGPRSNGTRTGCRIRIPGQGNLGPSAPKFSMLMVMGVLLRFCEDGFMGSCELEKRGDENGSRRKGDNGVGQVAAAIRTLGEGFVRMEKVKMDMASRIEEMRMEMELKRTEMILGSQQRIVEAFADAISERHTKKAKKMTMPEL*

>OfGT16

MGDLTESLTPARQVPFREDCWTEEATSTLVDAWGRRYLELNRGNLRQKDWQDVADAVNARHGHTKKTHRTDVQCKNRIDTLKKKYKIEKAKIAESKGTLTSSWPFFSRLNVLIGSNFNKQQQKFTSSPIPLVSQSTPSLSLPSPPMAVPLPFRKLPSAMFPTAILPQKRPFSSPSPPPPIVDESYFRRNYSAMAAAAAAAEDAPDAEDEGDSGGDEIEVSDDRGAEVEEGDEGMRRLAKAIERFGEIYERVESMKQRQMMELEKQRMQFSKELEVQRMQLFMDTQVQLEKIKQAKQSGSSDGMGLSFYMLDFWEVQHGLFTW*

>OfGT52

MGDLTESSTPTRQVPFREDCWTEEATSTLVDSWGRRYMELNRGNLRQKDWQEVADAVNARHGHTKKTHRTDVQCKNRIDTLKKKLKIEKAKIAESNGTLTSSWPFFSRLDVLIGSNFNKQQQKFMPLPIPLVSQSTSSLSLPSPPMAVPLPFRKFPSAMLPTAVLPQKRPYSSPSPPPPAVDESYFRRNYSAMAAAAAAEDAPDAEDEGDSGGEEIEVSDERGAEEEGDEGMRRLAKAIERFGEIYERVESMKQRQMVELEKQRMQFAKDLEVQRMQLFMDTQVQLEKIKRAKRSGSSDDMYS*

>OfGT19

MSSTTSVTSLVAAAVATDAVEKPPRRFPPPCWTQEETLALIDAYRERWYALRRGYLRTADWDAVAAIINSRFSDASIPKTSAQCRHKMEKLRQRYRAEKQRSLSYPYPAGYFISSWFFFDYMDAMENGTNPPSAETGSGGSPDNTALLKSFLDQSILKLKLKSKNSINSSPIFEAKNSEHTSYLDMGDNKAEQNHTSFGAKFRSEKTMGGFSMPPVKCKAKNSNISGGFLMKPYGNEDMIPPGLKIKKLDRRMNPGFNNGDEEYCSLRGSVKAMKRGRNPVEEMVESIKSLGEGLVKMEKAKMEMAREMETARMEMELKRNEMILDSQKQIVDAFVKGLFHVKKPKIATVSSDT*

>OfGT35

MSSTATATTLVAADVLATETVEKPPRRFPPPCWTQEETLALIDAYRERWYALRRGYLRTADWDAVAAIINSRFSDAPIPKTSAQCRHKMEKLRQRYRAEKQRSLSYPYPAGRFISSWFFFDYMDAMENGTDPPSADTGSGEKPDNTTLLKSFLDQSILKLKLKSKNNVNSSPILEAKNSEYTSYLDMGDNKEEQDLEQEEHTSFGTKFRSEKTMGGFSVPPLKLKAKNSSKHFGESNANFNISGGFPMKSSGNLDMTPPGLRIKKLGKPDRRMNPGFHYGDEEYWGLRESLNPGKAMNGNGIGIKRGRNPVEEMVESIKLLGEGLVKMEKAKMEMAREMETARMEMELKRNEMILESQRQIVDAFVKGLFELKKNKKSKIATVPSDS*

>OfGT1

MDDTQEDARYPPNPFGISNQEFLVHNATYTRNVGNEYVEDDDNDVNDEDLEYEDNDEDDGGGSSVQRIGKDDYVDDDGDEDEDEDDDDDDDYGNLQRHPKKRKLKSLMSSYEFAPRVPPPSSVTPAVPKPSYGGRNPHTDWTESETVILLDAWGDRFLKHGRKSLRSDEWQEVSKKVSQESKFERTDTQCRNRLDTLKKKYKKEKMKFGESEASTSKWVYFKKMDMLLSSGPQQAGLSCGVDSGEYTFMNPKVYLNSSNGMDEMRDSPGNSESTKVEEGESEGLPPKKTRNEMGNGASLKLLADSIQKFTDIYEKIENSKRQQMLELEKMRMDFRRDLELQKRQILERAQAEIVKIRRDLFSWKVKYSCPFFRCLK*

>OfGT40

MTYAQVAVYGFSDKVGLLSFPQREDAVGMTKPCSSKTAVIIDIEVREWVAKAYDRTLQLIEEHKEHVAEIAEWLLKKEAGFVEDKETKDTPTGKPVQDEGSPPLGPDIGQILPNLKSRTPANQLKTPALDDYITKCTLLIEVWNEVVSRMMMNIHECVKVYVRPPTSGSVSIVEIRKMDTSQSPSSWNYGVCIRTGPLRTSFVKFAPRVPAPSSATPAVPKPSYGGRNPHTDWTESETVILLDAWGDRFFKHGRKSLRSEEWQEVSKKVSQESKIERTDTQCRNRLDTLKKKYKKEKMKLGESWGSTSKWVYFKKMDMLLSSGPQQAGLSCGVDSGEYTFMNPKVYLNSSNGMDEMRDSPGNSESTKGEEGDPEGFPPNKTRNEMGNGASLKLLADSIQKC*

>AT1G54060

MEDDDEIQSIPSPGDSSLSPQAPPSPPILPTNDVTVAVVKKPQPGLSSQSPSMNALALVVHTPSVTGGGGSGNRNGRGGGGGSGGGGGGRDDCWSEEATKVLIEAWGDRFSEPGKGTLKQQHWKEVAEIVNKSRQCKYPKTDIQCKNRIDTVKKKYKQEKAKIASGDGPSKWVFFKKLESLIGGTTTFIASSKASEKAPMGGALGNSRSSMFKRQTKGNQIVQQQQEKRGSDSMRWHFRKRSASETESESDPEPEASPEESAESLPPLQPIQPLSFHMPKRLKVDKSGGGGSGVGDVARAILGFTEAYEKAETAKLKLMAELEKERMKFAKEMELQRMQFLKTQLEITQNNQEEEERSRQRGERRIVDDDDDRNGKNNGNVSS

>AT3G14180

MEDDEDIRSQGSDSPDPSSSPPAGRITVTVASAGPPSYSLTPPGNSSQKDPDALALALLPIQASGGGNNSSGRPTGGGGREDCWSEAATAVLIDAWGERYLELSRGNLKQKHWKEVAEIVSSREDYGKIPKTDIQCKNRIDTVKKKYKQEKVRIANGGGRSRWVFFDKLDRLIGSTAKIPTATSGVSGPVGGLHKIPMGIPMGSRSNLYHQQAKAATPPFNNLDRLIGATARVSAASFGGSGGGGGGGSVNVPMGIPMSSRSAPFGQQGRTLPQQGRTLPQQQQQGMMVKRCSESKRWRFRKRNASDSDSESEAAMSDDSGDSLPPPPLSKRMKTEEKKKQDGDGVGNKWRELTRAIMRFGEAYEQTENAKLQQVVEMEKERMKFLKELELQRMQFFVKTQLEISQLKQQHGRRMGNTSNDHHHSRKNNINAIVNNNNDLGNN

>AT3G58630

MDTVNDSFSPGSSRPSPATLSREDCWSEEATFTLIQAWGNRYVDLSRGNLRQKHWQEVANAVNDRHYNTGRNVSAAKSQPYRTDVQCKNRIDTLKKKYKVEKARVSESNPGAYISPWPFFSALDDLLRESFPTSSNPDSTDNIPHQRLSLPMSINPVPVAPRSAIPRRPATSPAIIPHAGDDLLGFRGNLNAFAAAAAAAACPASEDDSEGSRSRSSGRSGSNKKRERKIEKKQGYKEVADAIERLGQIYERVEEKKRKEMVELEKQRMRFAKELECHRMQLFTEMQVRLHKLRRTSGSKGPTSSASAALDYGMMDFPSYF

>AT3G11100

METTPETQSKTHRLPAGREDWWSEDATATLIEAWGDRYVNLNRGNLRQNDWKEVADAVNSSHGNGRPKTDVQCKNRIDTLKKKYKTEKAKPLSNWCFFDRLDFLIGPVMKKSSGAVVKSALMNPNLNPTGSKSTGSSLDDDDDDDDDDEEDDDDAGDWGFVVRKHRKVEDVDSSEGSAFRELARSILKLGEAFERIEGKKQQMMIELEKQRMEVAKELELQRMNMLMEMQLELEKSKLGKRRAASGKKL

>AT5G05550

METTTPQSKSSVSHRPPLGREDWWSEEATATLVEAWGNRYVKLNHGNLRQNDWKDVADAVNSRHGDNSRKKTDLQCKNRVDTLKKKYKTEKAKLSPSTWRFYNRLDVLIGPVVKKSAGGVVKSAPFKNHLNPTGSNSTGSSLEDDDEDDDEVGDWEFVARKHPRVEEVDLSEGSTCRELATAILKFGEVYERIEGKKQQMMIELEKQRMEVTKEVELKRMNMLMEMQLEIEKSKHRKRASASGKKNSH

>AT3G10030

MASCDDDFSLLGDDQSNPNQHHHHHQVLHHTPYAPRRFNPKPSNQILLPHHQRNGDEDDENDVVVEASSAFHGHGVNPFTADENSNPYDDNNAAVDEDEDEDLGANRSRIGGVRVEKRQSQEELSDGGTTNGGEITPYGSFKRPRTSSSSAGEYRKDREEWSDAAIACLLDAYSDKFTQLNRGNLRGRDWEEVASSVSERCEKLSKSVEQCKNKIDNLKKRYKLERHRMSSGGTAASHWPWFKKMEDIVGNSLALKGASDEDRSGSSMGNTVKPARRYPLVTYNPGVQINNVKSKATSNPRWRRVVLKISGAALACTGPNNIDPKVINLIAREVAMACRLGVEVAIVVGSRNFFCGSTWVTATGLDRTTAYHISMMASVMNSALLQSSLEKIGVQARLQTAISVQGVGEPYNRQRATRHLDKGRVVIFGGIGATLGNPLLSSDASAALRAIDINAEAVVKGTNVDGVYDCHSQDSNVTFEHISFQDLASRGLTSMDTMALNFCEENSIPVVVFNFLEAGNITKALCGEQVGTLIDRSGRGVS

>AT3G24490

MGDSEDETGYPKKFYSLNRQNHPMYSRPIPKRHAYYNEEEDEDEVEGEEEDPQGGYIRGNERFQKRQKPNKVVSGFEFAGPSDAKVAYDWREQEAFVLLEVWGDRFLQLGRRSLRNEDWNEVAEKVSEELRMEKSETQCRRMIDDLKRKYRKEKIKVEKSGLGSSKWSFFNKLDMLLCVSPKSDLGLACGVDSGEFVFMNTKVYLDKSNGFDEMMDSPGDSEEEEDEDDEVEYERKKVNDAASYKMLADSVERFGKVYEKMEKSKKEQMKELEKMRADFQRDLELQKKQIVDRAQSEIARLREEEENHHGGGDDDESEDEEMENDSDVNLSDE

>AT3G24860

MATPSPTSSPPSDSNPNSAATPPHQKQPPSPPQPTNPSSPPPHTTVVALAASTSAVARKTQPVLWTQDETLLLIESYKEKWFAIGRGPLKSTHWEEIAVAASSRSGVERTSTQCRHKIEKMRKRFRSERQSMGPISIWPFYNQMEELDSSNPAPISARPLTRLPPNSNNRYVDDEEEDEEEDNNNYEEEEEEDERQSKSRSINYILRRPGTVNRFAGVGGGLLSWGQKERSSKRKRNDGDGGERRRKGMRAVAAEIRAFAERVMVMEKKKIEFAKETVRLRKEMEIRRINLIQSSQTQLLQFINNAFDSF

>AT2G44730

MSDPDSPMNHDPIPDPSPLAPLPPPLSSSAHDDASTEPASNTDLKSASIPTASKNSRRLPPPCWSLEETIALIDAYRDKWYALNRGNLKANHWEEVAEAVGANCPDVILKKTAVQCRHKMEKLRKRYRTEIQRARSVPVARFISSWVHFKRMEAMENRPEIKQGNESGDDDDHDDGNYTARYQFSGGGGGARTTPRFFNRNGTAGSGGGGGSSSSGGIRIRIPTGVSIAQPGPRFPGKIDQKYTASPSAGVSSNPRAGRGIGAGGSSYGARVVRIPEGGGKRGREMMMKTEEDDNDPMVEIASAIKLLGDTLVRTEQTRMEMTREIEAMRMDTEMKRTKMILESQQRIVEAFAKSLSDYTTTEEQDKKKKAR

>AT3G54390

MENGESNQENLKSLNHDESLKKPSASSVVVDRLKRDEWSEGAVSTLLEAYESKWVLRNRAKLKGQDWEDVAKHVSSRATHTKSPKTQTQCKNKIESMKKRYRSESATADGSSWPLYPRLDHLLRGTQPQPQPQAVLPLNCSVPLLLLEPPLPAVAHPPQISYGSNGVGKIPKEDGFKPENKPEKDAEMDTDSSTPVVKTKVRGKKVKRRYKEEKEEIAGSIRWLAEVVMRSERARMETMKEIERMRAEAEAKRGELDLKRTEIMANTQLEIARIFAAAASSGQNKGVDSSLRIGRN

>AT4G17060

MGFAPVTPAAVETYDPDVDHDDESNGLDGFRVRSKRSGKFSGGYSDSPREVGDGYGVRSRARSNMKMYGGFKSEFDSDHDSGSGFGLKRKYNGNPKVSADFDADSDDEIVLVPKATRLRTHGKPSSGDFSHGSGGGFPLKSFGDRNFASHGFKPKNFSKPEPNFSQDLDYDDEFDDDRAEREGFNPRIQSSRSSSRVNGYSRKDGSYPRNTGASNGYGSSSRFKHEQMNAAAEVESDPIDEVVSSVKMLTEMFVRVENSKMEMMREMEKSRMEMELKHCQMMLESQQQIIGAFAEALSEKKSTNARRPVS

>AT5G63420

MMKPASLQGFSSHASSSIYSDVRRPATTPSKMAAFSALSLCPYTFTFRQSSRIKSTVSCSVTSAPASGTSSSSKTPRRRSGRLEGVGKSMEDSVKRKMEQFYEGTDGPPLRILPIGGLGEIGMNCMLVGNYDRYILIDAGIMFPDYDEPGIQKIMPDTGFIRRWKHKIEAVVITHGHEDHIGALPWVIPALDPNTPIFASSFTMELIKKRLKEHGIFVQSRLKTFSTRRRFMAGPFEIEPITVTHSIPDCSGLFLRCADGNILHTGDWKIDEAPLDGKVFDREALEELSKEGVTLMMSDSTNVLSPGRTISEKVVADALVRNVMAAKGRVITTQFASNIHRLGSIKAAADITGRKLVFVGMSLRTYLEAAWRDGKAPIDPSSLIKVEDIEAYAPKDLLIVTTGSQAEPRAALNLASYGSSHAFKLTKEDIILYSAKVIPGNESRVMKMMNRIADIGPNIIMGKNEMLHTSGHAYRGELEEVLKIVKPQHFLPIHGELLFLKEHELLGKSTGIRHTTVIKNGEMLGVSHLRNRRVLSNGFSSLGRENLQLMYSDGDKAFGTSSELCIDERLRISSDGIIVLSMEIMRPGVSENTLKGKIRITTRCMWLDKGRLLDALHKAAHAALSSCPVTCPLSHMERTVSEVLRKIVRKYSGKRPEVIAIATENPMAVRADEVSARLSGDPSVGSGVAALRKVVEGNDKRSRAKKAPSQEASPKEVDRTLEDDIIDSARLLAEEETAASTYTEEVDTPVGSSSEESDDFWKSFINPSSSPSPSETENMNKVADTEPKAEGKENSRDDDELADASDSETKSSPKRVRKNKWKPEEIKKVIRMRGELHSRFQVVKGRMALWEEISSNLSAEGINRSPGQCKSLWASLIQKYEESKADERSKTSWPHFEDMNNILSELGTPAS

>AT5G01380

MDRRNPFQHHHHHHQLHHHLIQQQQLPPPPLSTTATMDPGGGGGGGERIPQWSIEETKELLAIREELDQTFMETKRNKLLWEVVAAKMADKGFVRSAEQCKSKWKNLVTRYKACETTEPDAIRQQFPFYNEIQSIFEARMQRMLWSEATEPSTSSKRKHHQFSSDDEEEEVDEPNQDINEELLSLVETQKRETEVITTSTSTNPRKRAKKGKGVASGTKAETAGNTLKDILEEFMRQTVKMEKEWRDAWEMKEIEREKREKEWRRRMAELEEERAATERRWMEREEERRLREEARAQKRDSLIDALLNRLNRDHNDDHHNQGF

>AT1G13450

MFISDKSRPTDFYKDDHHNSSTTSTTRDMMIDVLTTTNESVDLQSHHHHNHHNHHLHQSQPQQQILLGESSGEDHEVKAPKKRAETWVQDETRSLIMFRRGMDGLFNTSKSNKHLWEQISSKMREKGFDRSPTMCTDKWRNLLKEFKKAKHHDRGNGSAKMSYYKEIEDILRERSKKVTPPQYNKSPNTPPTSAKVDSFMQFTDKGFDDTSISFGSVEANGRPALNLERRLDHDGHPLAITTAVDAVAANGVTPWNWRETPGNGDDSHGQPFGGRVITVKFGDYTRRIGVDGSAEAIKEVIRSAFGLRTRRAFWLEDEDQIIRCLDRDMPLGNYLLRLDDGLAIRVCHYDESNQLPVHSEEKIFYTEEDYREFLARQGWSSLQVDGFRNIENMDDLQPGAVYRGVR

>AT3G25990

MFVSDNNNPSRDINMMIGDVTSNGDLQPHQIILGESSGGEDHEIIKAPKKRAETWAQDETRTLISLRREMDNLFNTSKSNKHLWEQISKKMREKGFDRSPSMCTDKWRNILKEFKKAKQHEDKATSGGSTKMSYYNEIEDIFRERKKKVAFYKSPATTTPSSAKVDSFMQFTDKGFEDTGISFTSVEANGRPTLNLETELDHDGLPLPIAADPITANGVPPWNWRDTPGNGVDGQPFAGRIITVKFGDYTRRVGIDGTAEAIKEAIRSAFRLRTRRAFWLEDEEQVIRSLDRDMPLGNYILRIDEGIAVRVCHYDESDPLPVHQEEKIFYTEEDYRDFLARRGWTCLREFDAFQNIDNMDELQSGRLYRGMR

>AT2G38250

MDGHQHHHLHQLQYLNKHHLHTQSQTPEIASPVAVGDRFPQWSVEETKELIGIRGELDQTFMETKRNKLLWEVISNKMRDKSFPRSPEQCKCKWKNLVTRFKGCETMEAETARQQFPFYDDMQNIFTTRMQRMLWAESEGGGGGTSGAARKREYSSDEEEENVNEELVDVSNDPKILNPKKNIAKKRKGGSNSSNSNNGVREVLEEFMRHQVRMESEWREGWEAREKERAEKEEEWRRKMEELEKERLAMERMWRDREEQRRSREEMRAEKRDSLINALLAKLTRDGSL

>AT5G28300

MFDGGVPEQIHRFIASPPPPPPLPPHQPAAERSLPFPVSFSSFNTNHQPQHMLSLDSRKIIHHHHHHHHHDIKDGGATTGEWIGQTDHDDSDNHHQHHHHHPWCSDEVLALLRFRSTVENWFPEFTWEHTSRKLAEVGFKRSPQECKEKFEEEERRYFNSNNNNNNNTNDHQHIGNYNNKGNNYRIFSEVEEFYHHGHDNEHVSSEVGDNQNKRTNLVEGKGNVGETVQDLMAEDKLRDQDQGQVEEASMENQRNSIEVGKVGNVEDDAKSSSSSSLMMIMKEKKRKKRKKEKERFGVLKGFCEGLVRNMIAQQEEMHKKLLEDMVKKEEEKIAREEAWKKQEIERVNKEVEIRAQEQAMASDRNTNIIKFISKFTDHDLDVVQNPTSPSQDSSSLALRKTQGRRKFQTSSSLLPQTLTPHNLLTIDKSLEPFSTKTLKPKNQNPKPPKSDDKSDLGKRWPKDEVLALINIRRSISNMNDDDHKDENSLSTSSKAVPLWERISKKMLEIGYKRSAKRCKEKWENINKYFRKTKDVNKKRPLDSRTCPYFHQLTALYSQPPTGTTATTATTATSARDLDTRPEENRVGSQDPDISVPMHVDGDGAGDKSNVQFSGFDLEF

>AT5G47660

MELLAGDCRKRVGDDFEEDINPFDGSDGGCGWMYGTRQMGSNGNDDALATLADLASPPQKLKPIRCGVKLPSSSEDRHPLDILAGTLDRLPEMGFGCFEAPLGSKIADVEESGQLTRGFSKEEDDSLPPLQMEFQARNRISWDGLSLSSSVDSSDSDSSPDVRKTVTGKRKRETRVKLEHFLEKLVGSMMKRQEKMHNQLINVMEKMEVERIRREEAWRQQETERMTQNEEARKQEMARNLSLISFIRSVTGDEIEIPKQCEFPQPLQQILPEQCKDEKCESAQREREIKFRYSSGSGSSGRRWPQEEVQALISSRSDVEEKTGINKGAIWDEISARMKERGYERSAKKCKEKWENMNKYYRRVTEGGQKQPEHSKTRSYFEKLGNFYKTISSGEREK

>AT1G33240

MEQGGGGGGNEVVEEASPISSRPPANNLEELMRFSAAADDGGLGGGGGGGGGGSASSSSGNRWPREETLALLRIRSDMDSTFRDATLKAPLWEHVSRKLLELGYKRSSKKCKEKFENVQKYYKRTKETRGGRHDGKAYKFFSQLEALNTTPPSSSLDVTPLSVANPILMPSSSSSPFPVFSQPQPQTQTQPPQTHNVSFTPTPPPLPLPSMGPIFTGVTFSSHSSSTASGMGSDDDDDDMDVDQANIAGSSSRKRKRGNRGGGGKMMELFEGLVRQVMQKQAAMQRSFLEALEKREQERLDREEAWKRQEMARLAREHEVMSQERAASASRDAAIISLIQKITGHTIQLPPSLSSQPPPPYQPPPAVTKRVAEPPLSTAQSQSQQPIMAIPQQQILPPPPPSHPHAHQPEQKQQQQPQQEMVMSSEQSSLPSSSRWPKAEILALINLRSGMEPRYQDNVPKGLLWEEISTSMKRMGYNRNAKRCKEKWENINKYYKKVKESNKKRPQDAKTCPYFHRLDLLYRNKVLGSGGGSSTSGLPQDQKQSPVTAMKPPQEGLVNVQQTHGSASTEEEEPIEESPQGTEKPEDLVMRELIQQQQQLQQQESMIGEYEKIEESHNYNNMEEEEDQEMDEEELDEDEKSAAFEIAFQSPANRGGNGHTEPPFLTMVQ

>AT1G76890

MSGNSEGLLESSGGGVGGSVEEEKDMKMEETGEGAGSGGNRWPRPETLALLRIRSEMDKAFRDSTLKAPLWEEISRKMMELGYKRSSKKCKEKFENVYKYHKRTKEGRTGKSEGKTYRFFEELEAFETLSSYQPEPESQPAKSSAVITNAPATSSLIPWISSSNPSTEKSSSPLKHHHQVSVQPITTNPTFLAKQPSSTTPFPFYSSNNTTTVSQPPISNDLMNNVSSLNLFSSSTSSSTASDEEEDHHQVKSSRKKRKYWKGLFTKLTKELMEKQEKMQKRFLETLEYREKERISREEAWRVQEIGRINREHETLIHERSNAAAKDAAIISFLHKISGGQPQQPQQHNHKPSQRKQYQSDHSITFESKEPRAVLLDTTIKMGNYDNNHSVSPSSSRWPKTEVEALIRIRKNLEANYQENGTKGPLWEEISAGMRRLGYNRSAKRCKEKWENINKYFKKVKESNKKRPLDSKTCPYFHQLEALYNERNKSGAMPLPLPLMVTPQRQLLLSQETQTEFETDQREKVGDKEDEEEGESEEDEYDEEEEGEGDNETSEFEIVLNKTSSPMDINNNLFT

>AT1G76880

MMQLGGGTPTTTAAATTVTTATAPPPQSNNNDSAATEAAAAAVGAFEVSEEMHDRGFGGNRWPRQETLALLKIRSDMGIAFRDASVKGPLWEEVSRKMAEHGYIRNAKKCKEKFENVYKYHKRTKEGRTGKSEGKTYRFFDQLEALESQSTTSLHHHQQQTPLRPQQNNNNNNNNNNNSSIFSTPPPVTTVMPTLPSSSIPPYTQQINVPSFPNISGDFLSDNSTSSSSSYSTSSDMEMGGGTATTRKKRKRKWKVFFERLMKQVVDKQEELQRKFLEAVEKREHERLVREESWRVQEIARINREHEILAQERSMSAAKDAAVMAFLQKLSEKQPNQPQPQPQPQQVRPSMQLNNNNQQQPPQRSPPPQPPAPLPQPIQAVVSTLDTTKTDNGGDQNMTPAASASSSRWPKVEIEALIKLRTNLDSKYQENGPKGPLWEEISAGMRRLGFNRNSKRCKEKWENINKYFKKVKESNKKRPEDSKTCPYFHQLDALYRERNKFHSNNNIAASSSSSGLVKPDNSVPLMVQPEQQWPPAVTTATTTPAAAQPDQQSQPSEQNFDDEEGTDEEYDDEDEEEENEEEEGGEFELVPSNNNNNKTTNNL

>AT5G03680

MDQDQHPQYGIPELRQLMKGGGRTTTTTPSTSSHFPSDFFGFNLAPVQPPPHRLHQFTTDQDMGFLPRGIHGLGGGSSTAGNNSNLNASTSGGGVGFSGFLDGGGFGSGVGGDGGGTGRWPRQETLTLLEIRSRLDHKFKEANQKGPLWDEVSRIMSEEHGYQRSGKKCREKFENLYKYYRKTKEGKAGRQDGKHYRFFRQLEALYGDSNNLVSCPNHNTQFMSSALHGFHTQNPMNVTTTTSNIHNVDSVHGFHQSLSLSNNYNSSELELMTSSSEGNDSSSRRKKRSWKAKIKEFIDTNMKRLIERQDVWLEKLTKVIEDKEEQRMMKEEEWRKIEAARIDKEHLFWAKERARMEARDVAVIEALQYLTGKPLIKPLCSSPEERTNGNNEIRNNSETQNENGSDQTMTNNVCVKGSSSCWGEQEILKLMEIRTSMDSTFQEILGGCSDEFLWEEIAAKLIQLGFDQRSALLCKEKWEWISNGMRKEKKQINKKRKDNSSSCGVYYPRNEENPIYNNRESGYNDNDPHQINEQGNVGSSTSNANANANVTTGNPSGAMAASTNCFPFFMGDGDQNLWESYGLRLSKEENQ

>AT3G10000

MEDHQNHPQYGIEQPSSQFSSDLFGFNLVSAPDQHHRLHFTDHEISLLPRGIQGLTVAGNNSNTITTIQSGGCVGGFSGFTDGGGTGRWPRQETLMLLEVRSRLDHKFKEANQKGPLWDEVSRIMSEEHGYTRSGKKCREKFENLYKYYKKTKEGKSGRRQDGKNYRFFRQLEAIYGESKDSVSCYNNTQFIMTNALHSNFRASNIHNIVPHHQNPLMTNTNTQSQSLSISNNFNSSSDLDLTSSSEGNETTKREGMHWKEKIKEFIGVHMERLIEKQDFWLEKLMKIVEDKEHQRMLREEEWRRIEAERIDKERSFWTKERERIEARDVAVINALQYLTGRALIRPDSSSPTERINGNGSDKMMADNEFADEGNKGKMDKKQMNKKRKEKWSSHGGNHPRTKENMMIYNNQETKINDFCRDDDQCHHEGYSPSNSKNAGTPSCSNAMAASTKCFPLLEGEGDQNLWEGYGLKQRKENNHQ

>AT1G21200

MDGNFPQGGVVRSGASSYGGFDLQGSMRVHHQDSMNQQHRHNPNSRPLHEGLPFTMVTGQTCDHHQNQNMSMSEQQKAEREKNSVSDDDEPSFTEEGGDGVHNEANRSTKGSPWQRVKWTDKMVKLLITAVSYIGDDSSIDSSSRRKFAVLQKKGKWKSVSKVMAERGYHVSPQQCEDKFNDLNKRYKKLNDMLGRGTSCQVVENPALLDSIGYLNDKEKDDVRKIMSSKHLFYEEMCSYHNGNRLHLPHDLALQRSLQLALRSRDDHDNDDSRKHQMEDLDDEDHDGDGDEHDEYEEQHYAYGDCRVNHYGGGGGPLKKIRPSLSHEDGDHPSHVNSLECNKVSLPQIPFSQADVNQGGAESGRAGSVQKQWMESRTLQLEEQKLQIQVELLELEKQRFRWQRFSKKRDQELERMRMENERMKLENDRMGLELKQRELGVEL

>AT1G76870

MEGNCSQGRFDSQVSSMRDLRPNAINQNQKQHHPNSRQDSGFNNTMDTRHNNVDRGKKSMSEDDELCLLSSDGQNKSKENSPWQRVKWMDKMVKLMITALSYIGEDSGSDKKFAVLQKKGKWRSVSKVMDERGYHVSPQQCEDKFNDLNKRYKKLNEMLGRGTSCEVVENPSLLDKIDYLNEKEKDEVRRIMSSKHLFYEEMCSYHNGNRLHLPHDPAVQRSLHLITLGSRDDHDNDEHGKHQNEDLDDDDDYEEDHDGALSDRPLKRLRQSQSHEDVGHPNKGYDVPCLPRSQADVNRGISLDSRKAAGLQRQQIESKSLELEGRKLQIQAEMMELERQQFKWEVFSKRREQKLAKMRMENERMKLENERMSLELKRIELGAKL

>AT3G10040

MESNVMFSGFSPRMLSLEMPQNPPNPQNSIQFQHPHPYTTSGDQQTQPPIKSLYPYASKPKQMSPISGGGCDDEDRGSGSGSGCNPEDSAGTDGKRKLSQWHRMKWTDTMVRLLIMAVFYIGDEAGLNDPVDAKKKTGGGGGGGGGGGMLQKKGKWKSVSRAMVEKGFSVSPQQCEDKFNDLNKRYKRVNDILGKGIACRVVENQGLLESMDHLTPKLKDEVKKLLNSKHLFFREMCAYHNSCGHLGGHDQQPPQQNPISIPIPSQQQNCFHAAEAGKMARIAERVEVEEEVESDMAEDSESEMEESEEEETRKKRRISTAVKRLREEAASVVEDVGKSVWEKKEWIRRKMLEIEEKKIGYEWEGVEMEKQRVKWMRYRSKKEREMEKAKLDNQRRRLETERMILMLRRSEIELNELQSSGTRVDPSSAKG

>AT1G31310

MADQSGGLVMMREYRKGNWTLNETMVLIEAKRMDDERRMRRSIGLPPPEQQQDIRSNKPAELRWKWIEDYCWRKGCMRSQNQCNDKWDNLMRDYKKVREYERRRVESSITAGESSSSSAPAGETASYWKMEKSERKERSLPSNMLPQTYQALFEVVESKTLPSSTAVTAVTAAVAAAAAAISSGNGSGGGQIQKVIQQGLGFVVPKVHQIIQQQPVLLPLQPPPPPPPSQPLPRPLLLPPPPPPSFHAQPILPTVG

>AT2G35640

MADADPSSGEQIVMRECRKGNWTVSETLVLIEAKKMDDQRRVRRSEKQPEGRNKPAELRWKWIEEYCWRRGCYRNQNQCNDKWDNLMRDYKKIREYERSRVESSFNTVTSSSYWKMDKTERKEKNLPSNMLPQIYDVLSELVDRKTLPSSSSAAAAVGNGNGGQILRVCQQSLGFVAPMMAQPMHQIPTTIVLSLPPPPPQSLSLSLPSPPQPPPSSSFHAEPIPPTVGTSSTKRRRTTPGETTAGGEREVEEDAVGVALSRCTSVITQVIRENEEGQERRHKEVVRLQERRLKIEESKTEINRQGMNGLVDAINQLASSILALASSSCHNNRNHQGGPP

>AT2G33550

MALEQLGLGVSAVDGGENSSAPSNDGGDDGVKTARLPRWTRQEILVLIQGKRVAENRVRRGRAAGMALGSGQMEPKWASVSSYCKRHGVNRGPVQCRKRWSNLAGDYKKIKEWESQIKEETESYWVMRNDVRREKKLPGFFDKEVYDIVDGGVIPPAVPVLSLGLAPASDEGLLSDLDRRESPEKLNSTPVAKSVTDVIDKEKQEACVADQGRVKEKQPEAANVEGGSTSQEERKRKRTSFGEKEEEEEEGETKKMQNQLIEILERNGQLLAAQLEVQNLNLKLDREQRKDHGDSLVAVLNKLADAVAKIADKM

>AT4G31270

MEEGTSGSRRTRSQVAPEWAVKDCLVLVNEIAAVEADCSNALSSFQKWTMITENCNALDVSRNLNQCRRKWDSLMSDYNQIKKWESQYRGTGRSYWSLSSDKRKLLNLPGDIDIELFEAINAVVMIQDEKAGTESDSDPEAQDVVDLSAELAFVGSKRSRQRTMVMKETKKEEPRTSRVQVNTREKPITTKATHQNKTMGEKKPVEDMSTDEEEDETMNIEEDVEVMEAKLSYKIDLIHAIVGRNLAKDNETKDGVSMDDKLKSVRQQGDELIGCLSEIVSTLNRLHEVPQEIE

>Os01g21590

MGPRGVVGSAAMLGLEMHLAHPQMHAAAYQQPDPHGGGGGGFQQQVAAVRQQQQQSYSPYSAGASSRVIKAPGHDDGMGNGAGKGGVVQQQQQPGSVGCPWTRMKWTDGMVRLLINVVYSVGDDGDGVAAGGAAGGKASAGAAGHGKAGGSGSHGAHGQAAAQQKKGKWKSVSRAMMESGHMVSPQQCEDKFNDLNKRYKRVVDLLGRGKACKVVENHALLDAMDELTHKAKDEARKLLSSKHLFFREMCAYHNSGAAAAAAAHGPHGAGAAGVEATACFHHPPPASMAAASSAARQAAAAAPSLGMKDSSAGPEDDEDDSEDVPSSNEVDDEDDDDDDDDDEVGPGMKSRRIYGGHRVHHHHHHHNGHHKRRRGDDVSSAGAGDDDDEDGVKRARGAASAAGGGDDEGPSAVQQLQSELAAAVAGGGDPQQVRQWVRRRTVEVEEQQVAHEVRAYHLERQRLKWERFRANKERDMERARLRNDRLRIDGRRMLLLLRQKDLDFDIAEANSSSVDHLTSSAPPPLAALQQQQQPLGSSPS

TAGGHPN*

>Os01g48320

MDGKPPPPNPNLPYREDCWSDGETAALVDAWGRRYVDLNRGSLRQPQWREVAEAVNARPGASARRRPPRTDIQCKNRVDTLKKKYKAERARGTPSSWYFYRDLDMLVGPTLSAAAAAGGGGSAKKPSPPRGLPMMRRRLESPSRSPSPPSPTPAVALPLPNYRQASNLPSAGLLFNKMAAAAAAESDSEDGYNNPNNNYEDDEDDGSQQSASRSVSSRSGGVAAAGAGGGGVSSSKRKRGGGGGGGFGELARAVETFAEMYERMEFAKQRHAEEMERQRIKFLKDLELKRMQAFVDVQLQLAKAKHGKHPDGATEMLMSLAALPFLSTPAYL*

>Os01g52090

MDDGAAPAAAAAAELPSPSPSSSGTSPSPRSKRRRTDRYAQGFEFAPRPAPATATATAPAPTPARGTPEWSEGSTFALLDAWGDRFVRAGRRSLRADEWLEVSRLAAAAASRPPGYYSEQQCRNRIDTLRKKYRKEKERMRLAARRPDRPDRPSPSKWIYFDKMQSLMCPPPLPLQPPVVTRRRDTQPVPRQSWGLDAAEYVLGGCENAGTRDSGSGAELGEEQPNEAGAGKGEDFELLVESIRKLGDVYERVESSKRQHMAEVEWLRRDLQRDLEVRRREILEKAQAEIARLTEEDGEEGDLKEGEGDDNKRFGDDGGGEE*

>Os01g70230

MSGAANATSPAAGAGTPRSRLPRWTRHETLVLLQARRAMEHRGRRSPQPVRLKWAAVSTYCRRHGVERGPMQCRKRWGNLSWDLKKIVAWEKNLAAVVSGAGDNAVAAGEGEGEAPPPPRLESFWDMRGEQRRARQLPSSFDREVYDALVGGHGAAPPSDFGEDLADGDGVDADELPPPPLMVMPISATVSAKRGGAASDKNSTSQHDGGGGGGLKDSEATYGAGVGGEEGTTTATATATTTSIGRQVIEALERGNRMLGDQLEAQRAAWDAEREQRVALLAAVDKLAGAVCRIADKL*

>Os02g01380

MLAPPMADLPDAAAANPPHLNTSETSAGEDAHAVVQPQPQTQQPSGADAARRKRKRRRQEQLSASASFFERLVQRLMEHQESLHRQFLDTMERRERERAARDEAWRRQEADKFAREAAARAQDRASAAARESAIIAYLEKISGETITLPPPAANPAPGADEQDGVGKEVVAYDGEGSLQLSSSRWPKHEVEALIRVRTGLEDRFQEPGLKGPLWEEVSARMAAAGYRRNAKRCKEKWENINKYFRKAKESGKKRPAHAKTCPYFDELDRLYSRSGSGGGGGSSSAGGNGGEEAKGSSELLDAVVKYPDVRCAPPGFPFDGEQNEEGRTKDDGDEAHHDGDGDGDEEDVGVGVGRATDDHDDQVDESHDGH*

>Os02g07800

MAMDPDAAEWPCIIQALPALPPSPSSTGVPRLPTMVQALPAATDPPPAAARLRRGAEPPSPRRTRSGGAPEWTPAETLALVAEVAAVDDGWSRSVSAFQKWAIVAENLAASRGRAARGRGRGRAASECRRRWEALAAEYGVVRRWEVRGAGGYWRMSAAARRKAGLPADFDAEVYGAMEALTLVEEALLADATAGAGGAEIGEKSAAAAEVGEGDEVEAGEEDGNGDRGEVGEEDEGEVGEDGEEEDGNEMVEVEDDGNADDKDAQPDGGNAAASDDLGMQGQHMSSSLNSISTYWYLSKDEVCETGANNEGKKSQTDACELANKLQENAQHIHMLLKEEAGENENHNLAISSDSMETTRQKGDELIKSLGGLVSYLNQFTDLIKENGFENVVGLWQTLLPALASPFLSAIVYFSVWQSAVLGRFSALYSRIPGF*

>Os02g31160

MSTAGDPAAAAAAAAAATPSGRAPRLPRWTRQEILVLIEGKRMVEGRGGGRGGRGRAAAAAAAAAAAAAGGSGGEAAVAALEPKWAAVAEYCRRHGVERGAVQCRKRWSNLAGDYKKIKEWERAAAAAAPPREPSFWAMRNDARRERRLPGFFDREVYDILDGRGRGTLVSPSGGGNAAAGEEEPARAAAEVEVEVEEEEETGKTRARAEETVFDSGRPAAEESLFSDDEEEEDDDEAPPATAAAVAATAQAPPRAVIALPISGTSKDKQPEQQAASRGTPPPPPPTTQQQQGGQKRRRADDDEEEEDDGRRGGELQSKLVEILDRSSRMVAAQLEAQNANSRLDREQRRDQAASLAVVLGRLADALGRIADKL*

>Os02g33610

MVALASLSSLSPCGLARRRSASSAASISCCAAPPPPSAKGSQESRTPRRRVRKTEGATKSEDSVKRKMEQFYEGLDGPPLRVLPIGGLGEIGMNCMLVGNYDRYILIDAGVMFPDFDEFGVQKIIPDTTFIKKWSHKIEAVIITHGHEDHIGALPWVIPALDSSTPIFASSFTMELIKRRLKEFGIFLSSRLKVFRVRKRFQAGPFEVEPLRVTHSIPDCCGLVLRCADGIIFHTGDWKIDESPVDGKIFDRQALEELSKEGVTLMMSDSTNVLSPGRSISESVVAGSLLRHISEAKGRVITTQFASNIHRIGSIKAAADLTGRKLVFVGMSLRTYLDAAFRDGKSPIDPSTLVHLIESRFLLKVKVEDMDAYAPNDLLVVTTGSQAEPRAALNLASFGGSHALKLSKEDVLLYSAKVIPGNESRVMKMLNRLTELGPKIVMGKDAGLHTSGHAYHDELEEVLQIVKPQHFLPVHGELLFLKEHELLGRSTGIRHTTVIKNGEMLGVSHLRNRRVLSNGFVALGKEDLRLMYSDGDKAFGTSTDLCIDERLRIASDGVIFVSMEIFRPQKELASSQSGLKGKFKITTRCLWLDNGRLLDALYKAAYAALSSCPVNCPLSHMERMVSEILRKMVRKYSGKRPDVIAVATENTTVSFVEDSETKSSGKFGSFSAPRHSSRSSGRSLEESDKSCPENTEGEAKENLPDVLRTTRDDATTSSNGEAFFSSDLHKPKTLEHFWDSFKSPTAVKIARIVNGSAQGSKSKIGKISIVGKDSSDPSSAPVKSSKKNKWKPEEIKSLIQMRGEMNEQFQTVKGRMVLWEEISSSMLSQGISRTPAQCKSLWTSLVQKYEESKKDEESVKTWPYFLDMDRVLSSQGEMATQ*

>Os02g33770

MEGNLPPRGALVHGHGGGVGAFDLEATMQPPPPFHFAQDPHLHHHQGMVPVRGNPMLDLGNVVKTSPSDEEDVDDGHHHGGGGGSGKEASQWHRVKWISGMVKLLVSAVAYIDEDVDMDYGTGSAARRKHAMLKRKGKWRLVSAAMTERGFPVSPQQCEDKFNDLNKRYKRMTEILGRGTACQVVEHPELLEGMRLSGKLKEEARKHLNSKHLHYEEMCSYHNRNKMCLFDDPALQKSLRLALRSGEEHAKKNPFGYDDEDFSDDDDEDEEFDDLEVSAEDHHHGIHGAKRLKHDQEETHFGSNLSEVAVIDMNKMLSEGSGGPTAEKSPSTPGMRDIRLEKRRLKIKAQMLKIEQKHFKWLRFSKEKDRELEKMRLENEKMKLENERLELELKLKEIEMGIKPKKIFSD*

>Os02g35690

MDDDGGASPSSSLSPSRSPSPLPVADPVTVAAAPPGHLALAIPIPKPGSSSGGGGGGGGREDAWSDGATSTLIDAWGERFVALGRGSLRHPQWQEVAEVVSSRDGYSKQPKSDVQCKNRIDTLKKKYKVEKAKPDSSWPYFHRLDTLLAPVHKPAGAYPAAAAAGAAGAGNSGSNSAAAATAARSTAPMAPRVNFPQRTRTQFLPSSGVKRRMPSPPQVSASSESSDGFPPEPPMAAANGKRRREVEEEVNGADSGHRTQGLRELAQAIRRFGEVYERVELAKREQELRMERDRLEAARELEDQRVQFFLKMQMELSKANNAGASAAAAAVGAVATAIAAADGNGTRRTAMATDVGTSSNHHVRYRFQDSRHCHAAPQQPQHQYNENNAAEAARGTGNGSDTDNKEDEDEAEDEEDESQ*

>Os02g43300

MQHPQGAGDPPYGVVPAPPTMAAAFDLPPVTTPAPAAPSDVLLPTQPQVSGPEEFPAAAVNSNDDDMMMVDDVVVAGGVGGSGSTGNRWPREETLALIRIRSEMDAAFRNATLKAPVWEELSRRLAELGYQRSGKKCKEKFENVDKYYKRTKEGRTGRQDGKSYRFFSQLEALHAAAPPPPPQQRQGMPVEDPQPLAMAWMMLPGAADLGFLSMSSESESDDESDEEEEEEEAVAPGGGGREGLGDDGDGDGEGGSSTRKLMAMFEGMMRQVTEKQDAMQRVFLETLEKWEAERTEREEAWRRKEVARINREREQLSKERAAAASRDAALIAFLQRVGGAGGEPVRLSPSSAGATRHDAAAAGLQLVPVPAPRAKAEDAWAAAGGDGSGTTAPSRWPKEEVQALIDLRMEKEEQYNDMGPKGPLWEEIAAGMQRIGYNRSAKRCKEKWENINKYFKKVKESNKRRPEDSKTCPYFHQLDA

IYRKKHFAGRGGGGGGVTIAASHSSLAIVTVSEQDNPSQRELEGKSSNDVGNVQLAVPLLVHNAPDKKVEGSEGEPNVTAAAEETDSDEMCGEYTDDGDDDDKMQYKIEFQKPTAGGGGDGNDAPVPATTAAATSSAPTSNTSFLAVQ*

>Os03g02240

MPPFSAAGGEGAPSPISSRPPPPEQAAAAAAEEQLNGSSLEHDGVLGGEEGDRGGSSAGNRWPRQETLALLKIRSEMDAAFREAALKGPLWEEVSRKLAEMGYKRSAKKCREKFENVDKYYKRTKDGRAGRGDGKTYRFFTELEALHGAAAATARPPPVSLAPAPVAVAPPATPAGLSALRVHASPPPPPVKQHAAPPPPVMDVAACVMTMDDVSFSSGSDTEETAEEGGKRKRRGGGGIGGGGGGGKAMRMFEGLMRQVMERQEAMQQRLLEAIERRDQERMIREEAWRRQEVARLAREQDALAQERAIAASRDAAVISFIQRVTGQSIAAVPPPPLQPTPVASAAPPPPPQHHHQQTPPPIQVQPHHIMPMTPQPQLQPPQPQSKEANTVVRAAPPPQEQHDTAASGGGGGASSSRWPKAEVHALIQLRTELETRYQDSGPKGPLWEDISAGMRRLGYSRSSKRCKEKWENINKYFKKVKESNKKRPEDSKTCPYYHQLDALYRTKAANAAAAASASPAPATTTVLAPVPLSQTPPHVDHGGSNGNGNGWASANNGGGGSSSGGMQTKASNNGTATAGGLPVVSVAGGNGNGNGVAATTDNKGSKQVPVAKETAGQRQPQPLAMNHNYGNDRMADDMDSDSMDDDDDDDEFDDDENDDDIGGGKMQVQYETSSHFQRPQLQNQNVVVGRPNASGGGGGGAPTTPAGPPPPAATSGTSFLACVQ*

>Os03g18330

MSSSKPSPQQHQQQRANEWWSDGETAALIDAWGPPHVARSRGPLPAKDWRAAASAVNARRAAAGRRHNRTRAQCRARVQTLKERYKRELAKPPPSGWRHFSRLQEFLLAGPPPGFPPKTMPPASVKKEEEEECQDEAVGGGGGSGGLLGRWVVPTRPRNGAAAWCPVGVVVTKLAEVYERVELARLEVEKEKVAMEMEKAMQEAVKLKEEKLDT*

>Os03g18340

MATRRRAAAPPQPPAWTPEPWSDGETSALLDAWGPRHIRAAGGPLRTADWRACAAAVTARRAAAGRAPRTVDQCKNRLDYLKKRLKAERSRSKGAPAPPPPPPSVDRLRALLRLAPSVPPGFTSRGGAMPKVGEEEQEEEEEKAESFAAPLPRSWPSVPKRPRTAVALLPLSSSSGHQHGDGGGTPCTEVAAALDRLAGTYERVEVAKQKEATRLEERRLEAMRDLEIERMRILVDVAISASAVADTATAASSSW*

>Os03g46350

MDADVLLGQEEDEETMNAAMAASTAGSAMAAAAREYRRGNWTLPETMLLVEAKKRVSDGRRPAADQGLARWRWVEDYCWRRGCRRSQNQCNDRWDNLMRDYKKVRAHELAAAGGGGGGGPAESYWVMGRTERKEKGLPANLLREIYDAMGEVVERRPMSSGGGGGGAVFLAGASSSGSGGLADVPAMAMQASPLAQLLPRPLEATANCSSGSPERKRRRPSLDNEPPGGSTPPATTGRQGHQEHDDDDDEYAHHGADESSDDDGGLGGAIGRCAAILSVALENREASEERRHREVVAAEERRGRARQARREAGEQCMAGLATAVSQLAGSMLALAAKRRGPAAPK*

>Os04g32590

MSGGGEVSGRAPRLPRWTRQEILVLIEGKRVVEGRGRGRGRGGGGGAAAEPTKWAAVAEYCRRHGLERGPVQCRKRWSNLAGDYKKIREWERSLSSPSSSSAAAGMGKEVSFWAMRNDARRERRLPGFFDREVYDILEGRGGGNAAAAAAAGKEGEEEKAAVFDSGRAAAGGGGGGGDDGLFSSSEEEEDDDEATPPATTPAAAPAPPPAPAPAVPVLTSEKKSDPPRQDASEQAGTSRAKQPEQIVEDSPAQGAHKRQRSDDASGEAPDLQGQLIEILDRSSRMVAAQLEAQNINCQLDREQRKDQVSSLLGVLGKVADALYRIADKM*

>Os04g36790

MDDDDAAASASPSPSPSPVASALPVADPVTVAAGPPSGLLALALPIQKQQHAASPNPGGGGGGREDAWSEGATAALIDAWGERFVALGRGSLRHPQWQEVADAVSSREGYAKAPKSDVQCKNRIDTLKKKYKIERAKPASSWQFFGRLDDLLAPTFNQKPGGNGGGGVGASVNGRNPVPAALRVGFPQRSRTPLMPAPVSAVKRRAPSPEPSASSESSDGFPPERQPAFPPLPLPPPPNGKRSRADEGRGGGAGGGGDRAQGLRELAQAIRRFGEAYERVETAKLEQSAEMERRRLDFASELESQRVQFFLNTQMELSQVKDHSSSPANAAAPPGATGGAGGTSRRMASVNDASASGNYHRRYRVSDGGRHRHHPQPPPSRPHYQYHENNIAVAAAAAASDGEQSSDEEDDEEEESQ*

>Os04g40930

MLLSGPSPQQPTPPLLLPESSGEDGGHDSSSRAAASGGGGGPKKRAETWVQDETLCLIALRREMDSHFNTSKSNKHLWEAISARMREQGFDRSPTMCTDKWRNLLKEFKKARSHARGGGGGGVGGGGAGTGGGNCPAKMACYKEIDDLLKRRGKPTGGGGAAVGSGAVKSPTVTSKIDSYLQFDKGFEDASIPFGPVEASGRSLLSVEDRLEPDSHPLALTADAVATNGVNPWNWRDTSTNGGDNQVTFGGRVILVKWGDYTKRIGIDGTADAIKEAIKSAFGLRTRRAFWLEDEDEVVRSLDRDMPVGTYTLHLDTGMTIKLYMFENDEVRTEDKTFYTEEDFRDFLSRRGWTLLREYSGYRIADTLDDLRPGVIYEGMRSLGD*

>Os04g45750

MHAFAPPVPAPPPMSAMPPPPGPIQPAPISSAAPAVPLELPPQPPINLQGLSFSSMSGSESDDESEDDEMTAETGGSQDRLGKRKRGAGGKRLATFFEGLIKQVVDRQEEMQRRFLETMEKREAERTAREEAWRRQEVARLNREQEQLAQERAAAASRDAAIISFLQRIGGQSVQVPPAATVIQMPTPVQLQTPPPVKQPARQHQPQPTPPPPQAAPIPAAPLQQQPPQPQHKETIHHEAVTPRRAPPTSGSSLELVPAAEQHVESGLGGGEGGSASSSRWPKTEVQALIQLRMELDMRYQETGPKGPLWEEISSGMRRLGYNRSSKRCKEKWENINKYFKKVKESNKKRPEDSKTCPYFHQLDVIYRRKHLTGGGGGGASAANVAATAIEHQNPNRHEIEGKNINDNDKRKNGGGGGAQVPTSNGDTAPTTATFDVDSGMKKPEDIVRELSEQPPREFTTDETDSDDMGDDYTDDGEEGEDDGKMQYRIQFQRPNPGGANTAPPPATTPASAVPTSTPTSTFLAMVQ*

>Os04g45940

MASSSPTNAAAAARRVPPPCWTPDETLALARAYTARRLAVGRAHLTSADWAAVADAATPTKTARQCRHKVEKLRRRLRSKRRRPCPLLDAIDLLDGPSPSASPSQSPSPPPPASPPPLPPAPSSPPPKKRRLPDGDADADAEDDGESDVVKALRAIGEGFLRAEQRRMEAARKTQQMRMEMALRHLDSQRRLMEALVDRIIDSLE*

>Os04g51320

MEPGCGEGGGGGGGRDERVPQWGAQETRELIAARGEMERESAAAAAARRSAKTLWEAVSARLRERGYRRTAEQCKCKWKNLVNRYKGKETSDPENGRQCPFFDELHAVFTERARTMQQQLLESESGPSVKKKLKRPSGDLSSEDSDDEEDGSGDSGDEKPIRSRKRKIADKRQQSQRMAEKSRTSISSIHELLQDFLVQQQRMDIQWHEMMERRSQERIVFEQEWRQSMQKLEQERLMLEHTWMEREEQRRMREEARAEKRDALLTTLLNKVLQEDL*

>Os04g57530

MSGSSADPSPSASTAGAAVSPLALLRAHGHGHGHLTATPPSGATGPAPPPPSPASGSAPRDYRKGNWTLHETLILITANRLDDDRRAGVGGAAAGGGGAGSPPTPRSAEQRWKWVENYCWKNGCLRSQNQCNDKWDNLLRDYKKVRDYESRVAAAAATGGAAAANSAPLPSYWTMERHERKDCNLPTNLAPEVYDALSEVLSRRAARRGGATIAPTPPPPPLALPLPPPPPPSPPKPLVAQQQHHHHGHHHHPPPPQPPPSSLQLPPAVVAPPPASVSAEEEMSGSSESGEEEEGSGGEPEAKRRRLSRLGSSVVRSATVVARTLVACEEKRERRHRELLQLEERRLRLEEERTEVRRQGFAGLIAAVNSLSSAIHALVSDHRSGDSSGR*

>Os05g03740

MDPFHHLNSSFSNPYHPLLASPPHHFAPDPLPPPPPPPPPPLLPADPPSLERERLPQWSHAETAAFLAIRADLDHSFLSTKRNKALWEAASARLHALGFARTPDQCKSKWKNLVTRFKGTEGAAAVAAAGTDQMQGGQGGGAAARGSSSSFPFHDELRRIFDARVERARALERKKVKGKDPDDDHDGGGDGDEDDEEEADQQVDEEDDGASGDTTRAGSKKRRRKAAAAARRTGSTGGVEGEVEAMLREFMRRQIEMEERWMEAAEAREAERRAREEEWRTAMVALGEERLALVRRWREREDAWRARAEEREERRHQLVAALLAKLGAGDASA*

>Os05g48690

MEVREMAVAAAAAAAASSGGGGGGLRMPPPNPNLPYREDCWSEGETEALVRAWGSRYVELNRGNLRQKQWQEVADAVNSRRGAAARRRPPRTDVQCKNRVDTLKKKYKAERARVMPSTWSFFPELDRLVGPTLSASASKRPSPSPSPVPPPPYFAMPIHPSAVRKPPSPSPSPSPPPPMALPLPSYRRGSPLPAAALIQQEAAAAAAAAVSDSEDSEGPGDNNNHNAQRSPSQSVSSRSGNSNKRSRQEVDGGFRELARAIEAFAEMYERVESAKQKQALEIERQRIDFLKQLEVKRMENFVDAHVKLARAKRIKKHAGTAPDGIGAAELVSSVAALPFLSTSTYI*

>Os08g17100

MPLTLLCLAFSPFAWPPSLPPPPQTPDAIAGCKNKAEAGAGRWLRQDTLELLTIRPEMDTVLQEATLNGAIWEEISRICDVFFVGDFVM*

>Os08g37810

MAQEAASAAAGEEGALPQRRRKSAPGQPWAHMETMHLLDAYEERWTRLRRGQLKAQQWEEVAADVAARCAASGAALRKTGTQCRHKLEKLRKRYRIEGARPVTSLWPYFRRMERLERGPLPVSSAFPPPPPAASPPAAASDDDDDDEEDDEEEEEVEEPIPRNNTRSINGILRDSGGGGGGFSGFAPRPPPQQPPPSFAMLSTAPPRKRVPYEAFQAKVAMADKVKEEEPPPVATRPGGGTNEQLSAVLRDFGQGIMRLERRRMEMQWEIDRGWKETEARHNRMLLDAQRHLHEALAATPPPLKKARREHGGDGS*

>Os09g38570

MDALPDAADAAQLAAAAAPPQKRDEWSESGIVRLLEAYEAKWLLRNRAKLKWSDWVDIAHEVSAHCAMENAAATGKPGSSTAKTPNQCKNKIESMKKRYRAESAAAARAGPAAAGAGPSWRFFARMDGLLKGPAGSGQPQAELSNSIDLRAPPPAKVEVDVDADFVSQLADAGPGALSELVSAYANGSIQEKLDKVENSGHVEGRAAESDVNVSSPRIKEANEDAEEVDKVWDMSKKRKNTEFDIAKSIELLASSFLKIERARMDLYRETERMRVEAEIKKGEMELKRTEIMAKTHLQIAKLFAKRLKECSSKTGGSSSVTAEVDNHAKKGENGSG*

>Os10g37240

MAIYYLEREGLTHNMPMHGHFYTPLPLNHWHADTTNAADMTRGPMRMWAPHVSGMNFIFFISKEKVLMRVEMVSSPDVLIIPSPPFPSGNTRGGGGGGGGSSMQQQQPGGGGGGVQQFGAVAPEMSPFSPAGGGGGGRISMAEAASPISSRPPPAQQQFDELGVGGGGGGGGGFDAEALAAAAVGEEGASGGAGGNRWPRQETLALLKIRSDMDAAFRDATLKGPLWEEVSRKLAEEGYRRSAKKCKEKFENVHKYYKRTKESRAGRNDGKTYRFFTQLEALHGTAAGVVAAPSPVTSLAPPPATAVGVSGGVRAPAEPPPAVVMGNVMSFSTSNTEEYSDEEDSDDEGTEDMGGGGGDERGKRKRLSEGGAAAGVGDGAAGGDAAAVSGGHREAGAGPDDPRGGVAAAGDARLAREQEIPAGARHVGDAAVVSFIQKITGQTIPMPPIIAAPAITVMPPPAPSQQPQPPPPPSHPTPITSVAPAPPPPPPAAAAAAASQPSPQATKSPLPATPQTQSSMDIVMTAAEAHDAGYDGSGGGGGQPSSSRWPKAEVHALIQLRSNLDNRYQEAGPKGPLWEEISAGMRRLGYSRSSKRCKEKWENINKYFKKVKESNKKRPEDSKTCPYFHQLDALYRNKAALNSSSSSAAAAAPALPPPEHAEPAVTVAAPISQTPPPPPPQPVTTTKNGNGTSSTNGANGEGGGGGSGGMQMQASNGSVVAGNKFFTGAAAKKPEDIMKEMMEQRPQQPAAANNAFNRTDGGGGGGGVDSDNMDEDDEDDYDDDDDDDDDDVDGNKMQYEIQFQHQHHHQQPPQHRHQQSVVRPNAAASAAAGGNPPGTAAPATAAAATTTTGSFLAMVQ*

>Os10g41460

MSSSSSPPPNPDALSSPDLPPLAAPAAAAAAAAAAVSSGGAGGSGRRLPPPCWTHEETLALIEAYRDRWEGLRKGNLRASDWDDVAGAVTARCGRFPTATHKSGVQCRHKIEKLRKRYRAERARAAGRSKGPKWPFFPLLHDLAGGGAPDPSPNPIIKIKSKGPAAAAASPSPASPSPVSSPSSEEDEEEEAAADAGRSRSLHGLISNGGSGSGLRFTIPKASRSKPVAQREQPTAIKVEKSEEDAEAEAMAEVASALRAVGDKFLRMEERRLEISLQIEKERMESEMKRTQTLLDAQQLFVEAFLGKQQHHHHHHKKAKVISAAAAAATAAMDED*

>Os11g06410

MSILLWLSHVLLKLHYLRLYSCASSCTPVSILYGTNKKLKCLGDRFGEMEGSNPPGNMTQGPSYGSLDLHGISKQMHPPNSGNQGFNQPQIPGNFTIPMDRVTEPDNISDGVQLGQHGKIAHHHHHHRHHSKNHGSDEEEHDMNEDAADGKDKKGSPWHRMKWTDSMVKLLITAVSYTGEDPGADLGGGRRNYSMMQKKGKWKAISKVMGERGCHVSPQQCEDKFNDLNKRYKRLTDILGRGTACNVVENHSLLDHMDISEKMKEDARKILNSKHLFYEEMCSYHNNNRISLPEDPALQQSLQLALRCKEDNDFMRHASGDAELDDDQSEDSDYEENEEEHRAVDTNIRGPSMHKRMWHVVDHGDVGFVTSCSNDGSGRSDPYDVLDINKPFPDGCDLALVQKDLALKAAEIQKHRLQIETKAVQLAKQRLKWEMFRKNKDLELEKLALENEQMMLQNKRFELDLRHKELELEIKIKGNANHP*

>Os12g06640

MEGNNLPSGSLMRSNSGQMHAPNPGKQGFDHTQMPGNLSMHVNQSTDSDHLSEFQFGELGKVDHHHHHHHRQHAKNGMSDDEEHGVNEDATDSQSGKGKKGAAWQRMKWTDSMVKLLITAVSYTGEDPGADSGAGKRNSAIMQKKGKWKAISKVMGERGCSVSPQQCEDKFNDLNKRYKRLTDILGRGTACKIVENHALLDCMSNLSDKMKDDARKILSSKHLFYEEMCSYHNNNRVSLPEDPALQRSLQLALRCKDEHDLRRGTSGDADEDDQSVDSDSEEENDEENYTLQGDKSALPMHKRLRLMTDQEDVGFGNSSSSHGCSRRSDSHGISLDINKAFPDGTNLALAQKDLATQSADLEEQRLQIEVQAVYLAKQRLKWERFSKNKDRELEQMRLENEKMRLENKRLELEVRHKELELELKQKGSGNHA*
