# Supplementary material for: Insights into the trihelix transcription factor responses to salt and other stresses in Osmanthus fragrans
Source: BMC Genomics. 2022 Apr 30;23:334. doi: 10.1186/s12864-022-08569-7 (PMC9055724; doi:10.1186/s12864-022-08569-7)
Supplement: Supplementary file 3 — Additional file 3. [file 12864_2022_8569_MOESM3_ESM.doc]

**Additional file 3: Table S3.** WoLF PSORT program predicted the subcellular localization of OfGT proteins in *Osmanthus fragrans*.

| Gene ID | *Of*GT number | Prediction results |  |
| --- | --- | --- | --- |
| evm.model.Contig109.151 | *Of*GT1 | nucl |  |
| evm.model.Contig109.150 | *Of*GT2 | chlo |  |
| evm.model.Contig66.152 | *Of*GT3 | nucl |  |
| evm.model.Contig11.362 | *Of*GT4 | nucl |  |
| evm.model.Contig135.55 | *Of*GT5 | nucl |  |
| evm.model.Contig229.88 | *Of*GT6 | nucl |  |
| evm.model.Contig342.79 | *Of*GT7 | nucl |  |
| evm.model.Contig342.15 | *Of*GT8 | nucl |  |
| evm.model.Contig186.41 | *Of*GT9 | nucl |  |
| evm.model.Contig300.9 | *Of*GT10 | nucl |  |
| evm.model.Contig350.35 | *Of*GT11 | nucl |  |
| evm.model.Contig350.48 | *Of*GT12 | nucl |  |
| evm.model.Contig53.326 | *Of*GT13 | nucl |  |
| evm.model.Contig53.102 | *Of*GT14 | nucl |  |
| evm.model.Contig388.40 | *Of*GT15 | nucl |  |
| evm.model.Contig381.1 | *Of*GT16 | nucl |  |
| evm.model.Contig474.21 | *Of*GT17 | nucl |  |
| evm.model.Contig459.7 | *Of*GT18 | nucl |  |
| evm.model.Contig334.8 | *Of*GT19 | nucl |  |
| evm.model.Contig275.28 | *Of*GT20 | chlo |  |
| evm.model.Contig446.37 | *Of*GT21 | chlo |  |
| evm.model.Contig446.28 | *Of*GT22 | nucl |  |
| evm.model.Contig204.66 | *Of*GT23 | nucl |  |
| evm.model.Contig204.78 | *Of*GT24 | nucl |  |
| evm.model.Contig401.5 | *Of*GT25 | nucl |  |
| evm.model.Contig285.8 | *Of*GT26 | cyto |  |
| evm.model.Contig467.25 | *Of*GT27 | cyto |  |
| evm.model.Contig59.200 | *Of*GT28 | nucl |  |
| evm.model.Contig145.77 | *Of*GT29 | nucl |  |
| evm.model.Contig136.99 | *Of*GT30 | chlo |  |
| evm.model.Contig125.92 | *Of*GT31 | nucl |  |
| evm.model.Contig450.25 | *Of*GT32 | nucl |  |
| evm.model.Contig38.195 | *Of*GT33 | nucl |  |
| evm.model.Contig38.194 | *Of*GT34 | nucl |  |
| evm.model.Contig10.407 | *Of*GT35 | nucl |  |
| evm.model.Contig10.397 | *Of*GT36 | nucl |  |
| evm.model.Contig10.319 | *Of*GT37 | nucl |  |
| evm.model.Contig110.29 | *Of*GT38 | chlo |  |
| evm.model.Contig108.22 | *Of*GT39 | nucl |  |
| evm.model.Contig19.261 | *Of*GT40 | nucl |  |
| evm.model.Contig19.266 | *Of*GT41 | nucl |  |
| evm.model.Contig176.19 | *Of*GT42 | nucl |  |
| evm.model.Contig23.45 | *Of*GT43 | chlo |  |
| evm.model.Contig97.59 | *Of*GT44 | chlo |  |
| evm.model.Contig200.86 | *Of*GT45 | chlo |  |
| evm.model.Contig254.65 | *Of*GT46 | nucl |  |
| evm.model.Contig465.23 | *Of*GT47 | nucl |  |
| evm.model.Contig457.20 | *Of*GT48 | mito |  |
| evm.model.Contig77.121 | *Of*GT49 | nucl |  |
| evm.model.Contig77.118 | *Of*GT50 | nucl |  |
| evm.model.Contig126.84 | *Of*GT51 | nucl |  |
| evm.model.Contig197.60 | *Of*GT52 | nucl |  |
| evm.model.Contig610.12 | *Of*GT53 | nucl |  |
| evm.model.Contig476.43 | *Of*GT54 | nucl |  |
| evm.model.Contig159.114 | *Of*GT55 | nucl |  |
| evm.model.Contig159.113 | *Of*GT56 | nucl |  |
