# Supplementary material for: Insights into the trihelix transcription factor responses to salt and other stresses in Osmanthus fragrans
Source: BMC Genomics. 2022 Apr 30;23:334. doi: 10.1186/s12864-022-08569-7 (PMC9055724; doi:10.1186/s12864-022-08569-7)
Supplement: Supplementary file 4 — Additional file 4. [file 12864_2022_8569_MOESM4_ESM.docx]

| Motif | Width | Best possible match |
| --- | --- | --- |
| 1 | 29 | KGYNRSPKOCKEKWENLNKYYKKEKEKLA |
| 2 | 79 | DFIERLMKDVMDKQEEWQEKFLETJEKKEQDRIAREEAWRVQEMDRIEREHKFLAQERAIIAARDAAVMEFLQKITGKZ |
| 3 | 15 | GRWPEZETLALJEAR |
| 4 | 57 | RGTSCEVVENPSLLDMMDHISEKAKEEVKKJLSSKHLHYEEMCSYHNGNRLHLPPDP |
| 5 | 80 | GMKANTLQKQWJNHRSLQLEEQRLHIQAQMLELEKZRFKWQRFCQKKDRELEMEKLEIERMKLENERMALELRQKEIGID |
| 6 | 15 | LKKPLWEEVSRKMAE |
| 7 | 40 | GGEGVRELAEAIRRFGEIYEKVEKAKMEQMRELEKMRMEF |
| 8 | 41 | DPSGGKKASPWORVKWTDNMVRLLITAVSYISEEAASEYGD |
| 9 | 21 | GKTWPFFDQLDALYGERPQTA |
| 10 | 85 | TNIKEILEEFMKQQIQMEMQNJKAYEEREEERRIKEMEWRQTMEBLENERIMMDRRWREREEQRRIREETRAEKRDTLITAJLNK |
| 11 | 57 | QEAYQAPKJSDTSLSLSNSSDFDTTSSDGGDVNGGIBDDSSGNRQKKRGKRRWKAKI |
| 12 | 68 | AISAAGAVIAEAISPWEEREERGHGEVLSLHDGRLITEELGDYTKRIGIBGTVDAIKELIKSILRLRS |
| 13 | 200 | CRYGVZVAIVVGGRNFFCGDTWVTSTGLDRCTAYQIGMMATVMNSILLIQSALEKLGVQTRMQSAFSMPEVAEPYSRQRATRHLEKGRVIFGGIGAGTGNPLFSTDTAAALRASETHADAVLKGANMDGVFVCDPRNNNIAAEHISFRDLASRGALPMDMMAMTFCEENGIPVIIFNLHEPGNISRALCGEOVGTLIDOT |
| 14 | 40 | GLSCGVDSGEYTFMNPKVYLBSSNGMDEMRDSPGNSESTK |
| 15 | 41 | IGREASPSNSNTGNAMNDSCFRYFMGDDHNTWENYGLKLNK |

**Additional file 4: Table S4.** MEME motif sequences and lengths of *OfGT* genes family proteins in *Osmanthus fragrans*
