# Supplementary material for: Insights into the trihelix transcription factor responses to salt and other stresses in Osmanthus fragrans
Source: BMC Genomics. 2022 Apr 30;23:334. doi: 10.1186/s12864-022-08569-7 (PMC9055724; doi:10.1186/s12864-022-08569-7)
Supplement: Supplementary file 6 — Additional file 6. [file 12864_2022_8569_MOESM6_ESM.doc]

**Additional file 6: Table S6.** Ka/Ks ratios and divergence times (Mya) of the tandemly and segmentally duplicated *OfGT* genes.

| Duplicated Gene Pairs | Ka | Ks | Ka/Ks | Type of duplication | Type of Selection | Divergence time (Mya) |
| --- | --- | --- | --- | --- | --- | --- |
| OfGT3vs.OfGT9 | 0.93 | 2.42 | 0.34 | Segmental | Purifying | 80.70 |
| OfGT2vs.OfGT11 | 0.64 | 3.01 | 0.21 | Segmental | Purifying | 100.17 |
| OfGT1vs.OfGT34 | 0.14 | 0.78 | 0.18 | Segmental | Purifying | 26.12 |
| OfGT2vs.OfGT33 | 0.24 | 1.19 | 0.20 | Segmental | Purifying | 39.78 |
| OfGT3vs.OfGT42 | 0.05 | 0.20 | 0.26 | Segmental | Purifying | 6.76 |
| OfGT4vs.OfGT40 | 0.26 | 0.53 | 0.50 | Segmental | Purifying | 17.59 |
| OfGT3vs.OfGT49 | 0.40 | 3.39 | 0.12 | Segmental | Purifying | 112.84 |
| OfGT1vs.OfGT50 | 0.08 | 0.21 | 0.40 | Segmental | Purifying | 6.90 |
| OfGT2vs.OfGT49 | 0.07 | 0.30 | 0.22 | Segmental | Purifying | 10.10 |
| OfGT1vs.OfGT47 | 0.13 | 0.61 | 0.21 | Segmental | Purifying | 20.18 |
| OfGT1vs.OfGT55 | 0.17 | 0.74 | 0.23 | Segmental | Purifying | 24.72 |
| OfGT2vs.OfGT56 | 0.25 | 1.22 | 0.20 | Segmental | Purifying | 40.67 |
| OfGT5vs.OfGT16 | 0.56 | 2.04 | 0.27 | Segmental | Purifying | 67.85 |
| OfGT5vs.OfGT15 | 0.49 | 1.58 | 0.31 | Segmental | Purifying | 52.65 |
| OfGT5vs.OfGT37 | 0.92 | 2.25 | 0.41 | Segmental | Purifying | 75.09 |
| OfGT5vs.OfGT52 | 0.42 | 1.83 | 0.23 | Segmental | Purifying | 60.84 |
| OfGT5vs.OfGT51 | 0.08 | 0.34 | 0.23 | Segmental | Purifying | 11.28 |
| OfGT8vs.OfGT28 | 0.16 | 0.79 | 0.20 | Segmental | Purifying | 26.40 |
| OfGT11vs.OfGT28 | 0.21 | 0.78 | 0.26 | Segmental | Purifying | 26.02 |
| OfGT10vs.OfGT21 | 0.38 | 1.55 | 0.24 | Segmental | Purifying | 51.64 |
| OfGT11vs.OfGT19 | 0.21 | 0.78 | 0.26 | Segmental | Purifying | 26.02 |
| OfGT12vs.OfGT20 | 1.06 | 2.41 | 0.44 | Segmental | Purifying | 80.27 |
| OfGT9vs.OfGT27 | 0.17 | 1.43 | 0.12 | Segmental | Purifying | 47.59 |
| OfGT9vs.OfGT26 | 0.16 | 1.44 | 0.11 | Segmental | Purifying | 47.84 |
| OfGT8vs.OfGT24 | 0.99 | 3.46 | 0.29 | Segmental | Purifying | 115.23 |
| OfGT11vs.OfGT24 | 0.06 | 0.23 | 0.25 | Segmental | Purifying | 7.63 |
| OfGT12vs.OfGT23 | 0.05 | 0.25 | 0.22 | Segmental | Purifying | 8.27 |
| OfGT10vs.OfGT36 | 0.20 | 1.19 | 0.17 | Segmental | Purifying | 39.74 |
| OfGT9vs.OfGT35 | 0.67 | 2.29 | 0.29 | Segmental | Purifying | 76.22 |
| OfGT11vs.OfGT35 | 0.23 | 0.74 | 0.31 | Segmental | Purifying | 24.53 |
| OfGT12vs.OfGT37 | 1.17 | 2.48 | 0.47 | Segmental | Purifying | 82.75 |
| OfGT8vs.OfGT54 | 0.04 | 0.26 | 0.16 | Segmental | Purifying | 8.60 |
| OfGT8vs.OfGT42 | 1.01 | 4.04 | 0.25 | Segmental | Purifying | 134.64 |
| OfGT15vs.OfGT51 | 0.40 | 2.21 | 0.18 | Segmental | Purifying | 73.67 |
| OfGT18vs.OfGT53 | 0.10 | 0.30 | 0.35 | Segmental | Purifying | 9.95 |
| OfGT15vs.OfGT52 | 0.04 | 0.29 | 0.15 | Segmental | Purifying | 9.69 |
| OfGT16vs.OfGT52 | 0.23 | 0.69 | 0.33 | Segmental | Purifying | 23.14 |
| OfGT16vs.OfGT51 | 0.56 | 2.11 | 0.26 | Segmental | Purifying | 70.27 |
| OfGT16vs.OfGT37 | 0.81 | 2.66 | 0.31 | Segmental | Purifying | 88.63 |
| OfGT16vs.OfGT20 | 0.87 | 3.22 | 0.27 | Segmental | Purifying | 107.25 |
| OfGT20vs.OfGT38 | 0.73 | 2.98 | 0.25 | Segmental | Purifying | 99.23 |
| OfGT20vs.OfGT51 | 0.90 | 1.92 | 0.47 | Segmental | Purifying | 63.93 |
| OfGT19vs.OfGT24 | 0.18 | 0.70 | 0.26 | Segmental | Purifying | 23.17 |
| OfGT21vs.OfGT36 | 0.24 | 0.49 | 0.49 | Segmental | Purifying | 16.26 |
| OfGT22vs.OfGT35 | 0.05 | 0.24 | 0.21 | Segmental | Purifying | 7.98 |
| OfGT19vs.OfGT35 | 0.05 | 0.24 | 0.21 | Segmental | Purifying | 7.98 |
| OfGT20vs.OfGT37 | 0.07 | 0.38 | 0.18 | Segmental | Purifying | 12.53 |
| OfGT25vs.OfGT29 | 0.06 | 0.23 | 0.26 | Segmental | Purifying | 7.81 |
| OfGT24vs.OfGT35 | 0.21 | 0.79 | 0.27 | Segmental | Purifying | 26.19 |
| OfGT28vs.OfGT54 | 0.15 | 0.67 | 0.23 | Segmental | Purifying | 22.19 |
| OfGT33vs.OfGT42 | 0.42 | 2.33 | 0.18 | Segmental | Purifying | 77.69 |
| OfGT33vs.OfGT56 | 0.06 | 0.25 | 0.24 | Segmental | Purifying | 8.28 |
| OfGT34vs.OfGT55 | 0.09 | 0.29 | 0.32 | Segmental | Purifying | 9.50 |
| OfGT33vs.OfGT49 | 0.20 | 1.32 | 0.15 | Segmental | Purifying | 44.09 |
| OfGT34vs.OfGT50 | 0.18 | 0.75 | 0.24 | Segmental | Purifying | 25.15 |
| OfGT34vs.OfGT47 | 0.12 | 0.61 | 0.20 | Segmental | Purifying | 20.36 |
| OfGT30vs.OfGT44 | 0.42 | 1.63 | 0.26 | Segmental | Purifying | 54.48 |
| OfGT32vs.OfGT43 | 0.07 | 0.27 | 0.27 | Segmental | Purifying | 8.90 |
| OfGT42vs.OfGT49 | 0.42 | 2.68 | 0.16 | Segmental | Purifying | 89.20 |
| OfGT38vs.OfGT52 | 1.01 | 1.92 | 0.53 | Segmental | Purifying | 63.97 |
| OfGT38vs.OfGT51 | 0.87 | 2.12 | 0.41 | Segmental | Purifying | 70.82 |
| OfGT47vs.OfGT55 | 0.17 | 0.61 | 0.28 | Segmental | Purifying | 20.42 |
| OfGT49vs.OfGT56 | 0.19 | 1.26 | 0.15 | Segmental | Purifying | 41.91 |
| OfGT50vs.OfGT55 | 0.21 | 0.91 | 0.24 | Segmental | Purifying | 30.25 |
| OfGT47vs.OfGT50 | 0.17 | 0.56 | 0.30 | Segmental | Purifying | 18.71 |
| OfGT42vs.OfGT56 | 0.41 | 1.91 | 0.21 | Segmental | Purifying | 63.72 |
| OfGT1vs.OfGT2 | 0.27 | 0.48 | 0.56 | Tandem | Purifying | 16 |
| OfGT33vs.OfGT34 | 0.41 | 1.96 | 0.21 | Tandem | Purifying | 65.33 |
| OfGT55vs.OfGT56 | 0.34 | 0.79 | 0.43 | Tandem | Purifying | 26.33 |

Ka, non-synonymous; Ks, synonymous; Mya, Million years ago.
