# Supplementary material for: Insights into the trihelix transcription factor responses to salt and other stresses in Osmanthus fragrans
Source: BMC Genomics. 2022 Apr 30;23:334. doi: 10.1186/s12864-022-08569-7 (PMC9055724; doi:10.1186/s12864-022-08569-7)

**Additional file 8: Figure S1.** Correlation analysis of 12 *OfGT* genes under multiple stresses (salt/drought/waterlogging MeJA/ABA/GA_3_) conditions.


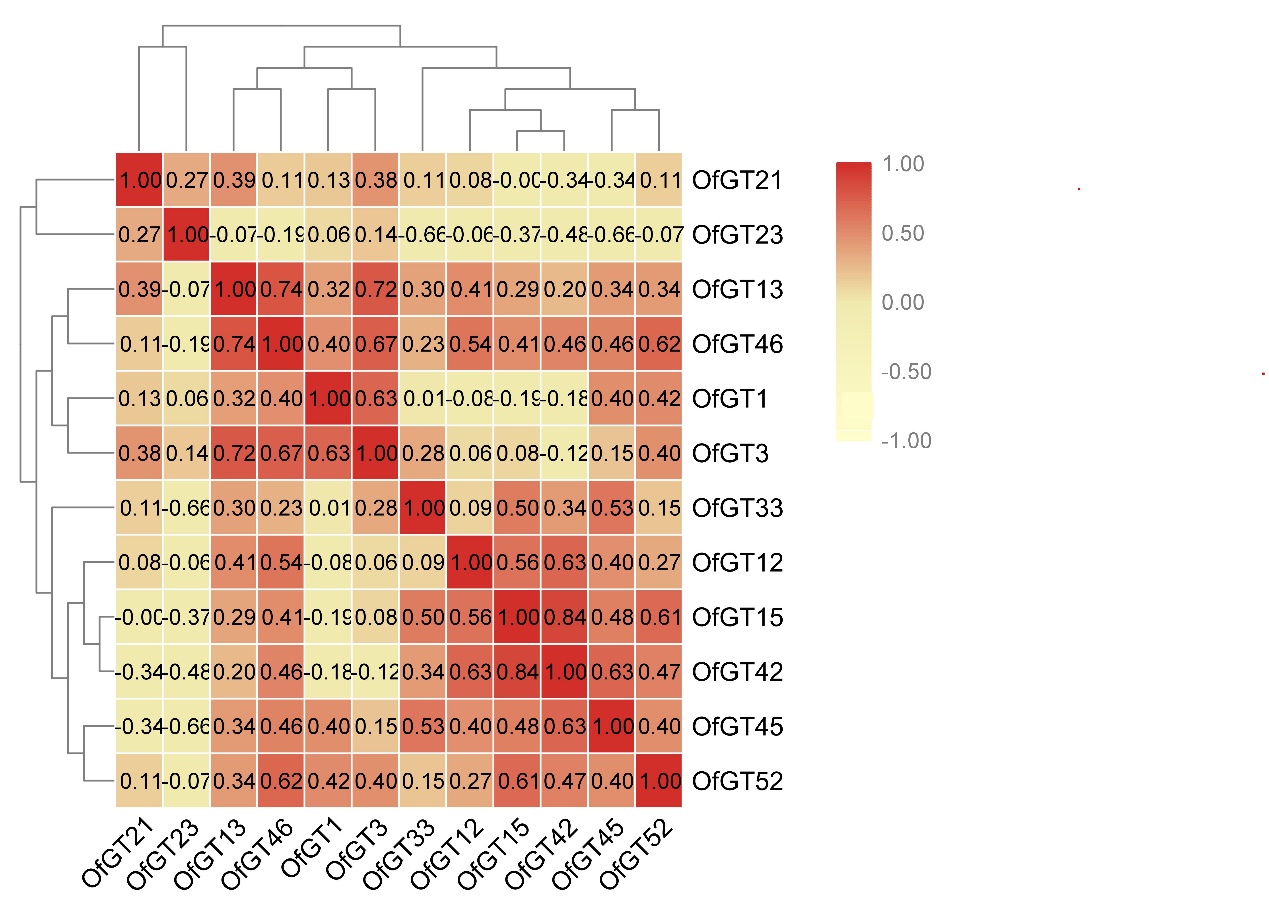

Supplement: Supplementary file 8 — Additional file 8. [file 12864_2022_8569_MOESM8_ESM.docx]
