# Supplementary material for: Insights into the trihelix transcription factor responses to salt and other stresses in Osmanthus fragrans
Source: BMC Genomics. 2022 Apr 30;23:334. doi: 10.1186/s12864-022-08569-7 (PMC9055724; doi:10.1186/s12864-022-08569-7)
Supplement: Supplementary file 9 — Additional file 9. [file 12864_2022_8569_MOESM9_ESM.docx]

**Additional file 9: Figure S2.** The transcriptional activation levels of *OfGT3/42/46.*


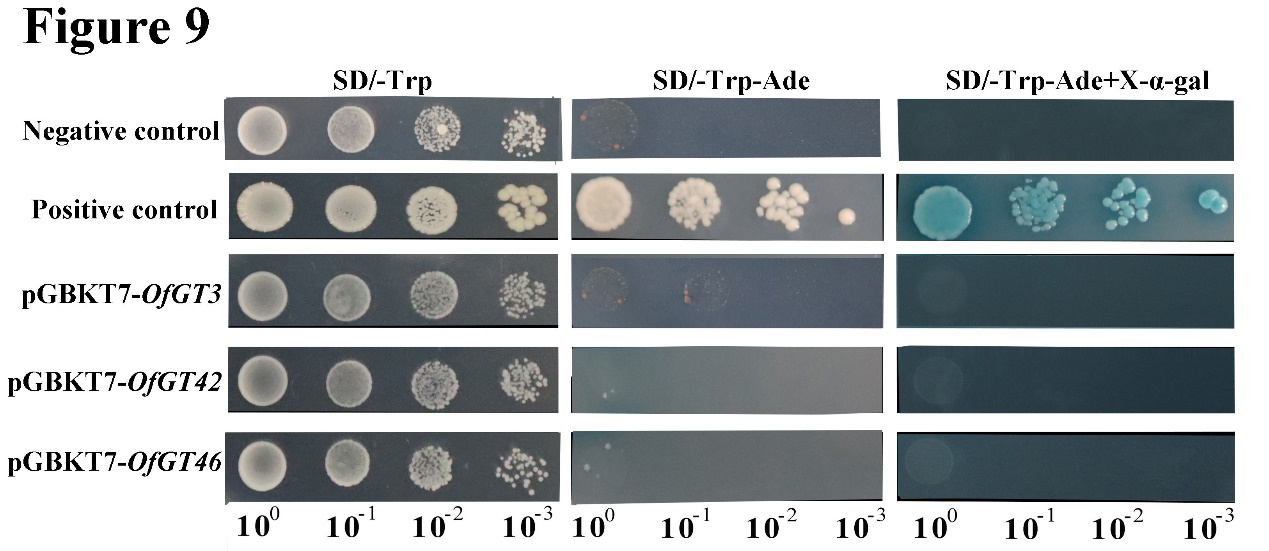
The growth conditions of yeast AH109 containing the negative control (pGBKT7), positive control, and the pGBKT7-OfGT3, pGBKT7-OfGT42, and pGBKT7-OfGT46 vectors SD/-Trp, SD/-Trp-Ade, and SD/-Trp-Ade+X-α-gal media.
