# Supplementary figures and images for: Insights into the trihelix transcription factor responses to salt and other stresses in Osmanthus fragrans
Source: BMC Genomics. 2022 Apr 30;23:334. doi: 10.1186/s12864-022-08569-7 (PMC9055724; doi:10.1186/s12864-022-08569-7)

**Additional file 10: Figure S3.** The original uncropped gel of RT-PCR analysis


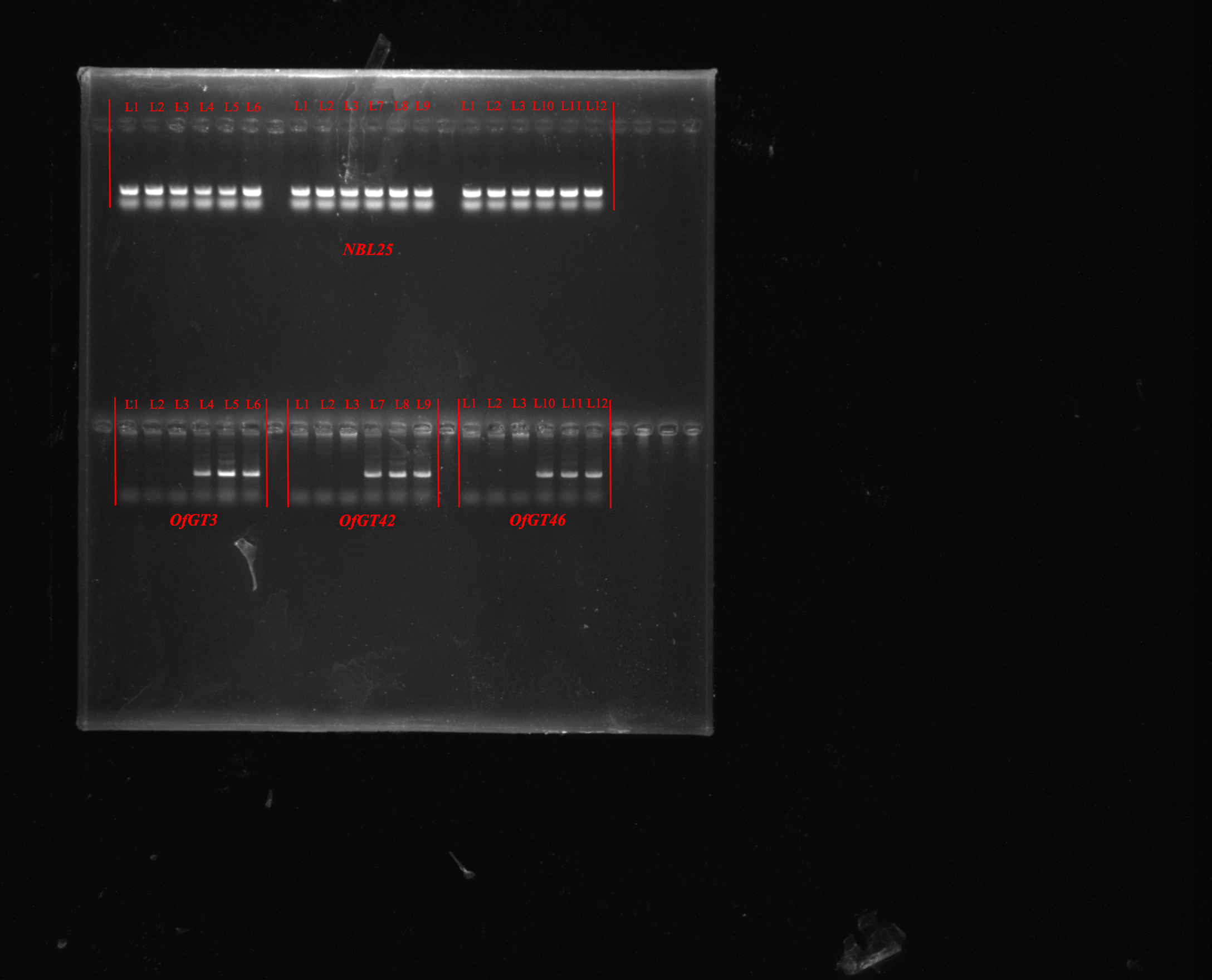

Supplement: Supplementary file 10 — Additional file 10. [file 12864_2022_8569_MOESM10_ESM.docx]
