# Supplementary material for: Insights into the trihelix transcription factor responses to salt and other stresses in Osmanthus fragrans
Source: BMC Genomics. 2022 Apr 30;23:334. doi: 10.1186/s12864-022-08569-7 (PMC9055724; doi:10.1186/s12864-022-08569-7)

**Additional file 11:** **Figure S4.** The homology levels *OfGT3/42/46* with *NP_001236630.1*/*NP_001236643.1.* DNAMAN software was use for multiple sequence alignments of *OfGT3/42/46* and *NP_001236630.1*/*NP_001236643.1*. **a** The multiple sequence alignments result of *OfGT3/42* and *NP_001236630.1*. **b** The multiple sequence alignments result of *OfGT46* and *NP_001236643.1*.


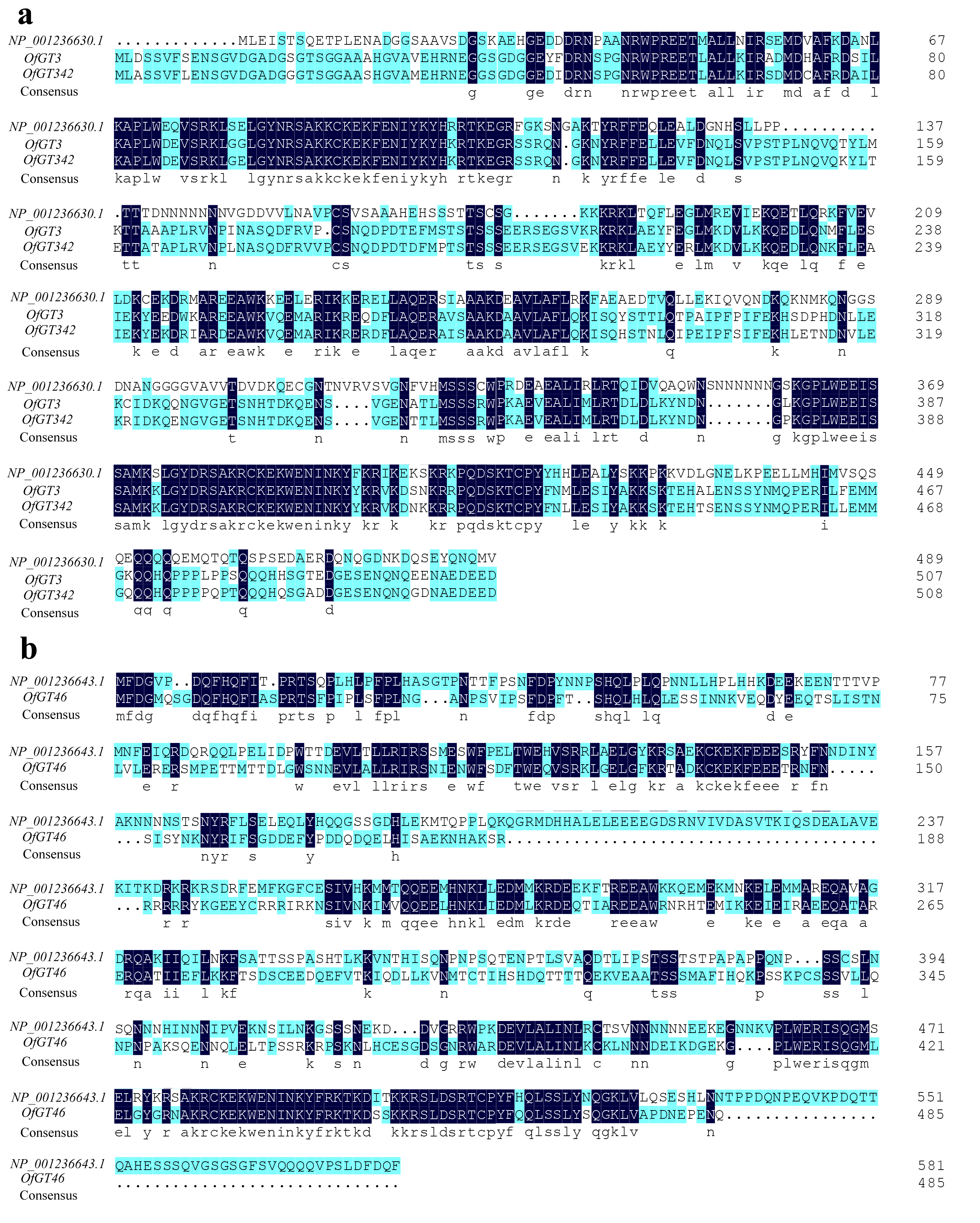

Supplement: Supplementary file 11 — Additional file 11. [file 12864_2022_8569_MOESM11_ESM.docx]
