# Supplementary material for: Insights into the trihelix transcription factor responses to salt and other stresses in Osmanthus fragrans
Source: BMC Genomics. 2022 Apr 30;23:334. doi: 10.1186/s12864-022-08569-7 (PMC9055724; doi:10.1186/s12864-022-08569-7)
Supplement: Supplementary file 12 — Additional file 12. [file 12864_2022_8569_MOESM12_ESM.doc]

**Additional file 12: Table S8.** Primers of *OfGT* genes used in the experiments.

|  |  | Primer sequence (5'→3') | Primer sequence (3→'5') | |
| --- | --- | --- | --- | --- |
| Gene number | *OfGT* number | Forward | | Reverse |
| evm.model.Contig109.151 | *OfGT1* | CTTGATGTTGAGGACTTGGTTGA | | AAAGAAGAATTATAGATATGCTCAACCA |
| evm.model.Contig66.152 | *OfGT3* | CAAGAAGAAAACGCTGAGGATG | | TGACCTTATGTAAATGTCTGCTGC |
| evm.model.Contig350.48 | *OfGT12* | GATGGCTATGACATTCTGTGAGGA | | GATAACGAAGTCCCCCCAAA |
| evm.model.Contig53.326 | *OfGT13* | CAGAATACGCTGACGCTTGC | | GCCACATCTTTCCAGTGCTTT |
| evm.model.Contig388.40 | *OfGT15* | TCTTCTCTGTCAAATCTTCACACTCA | | CAGGCGTCAACGATTCCG |
| evm.model.Contig446.37 | *OfGT21* | ATGCTTTTCCAACATCACCTCTT | | TGTCTGAAAACAACCGCACC |
| evm.model.Contig204.66 | *OfGT23* | ATGGGATTCCAGTTATCATTTTCA | | TGCTAGACCAGTGCCGTGTTT |
| evm.model.Contig38.195 | *OfGT33* | TGGAAAGCAATACCCCACAAC | | CATTTACTGGTTTTGGACTTGACA |
| evm.model.Contig176.19 | *OfGT42* | AGTCAGGGGCAGACGATGG | | TCTGCTTCTCATCAGTCATCACCT |
| evm.model.Contig200.86 | *OfGT45* | ATCTCCTCATCCTGGGTACACTTC | | CCTCTTCTCCTTGCGGCTC |
| evm.model.Contig254.65 | *OfGT46* | TTTCCTATTCCTCTTTCTTTTCCA | | TCGGTAGTCATCGTCGTTTCTG |
| evm.model.Contig197.60 | *OfGT52* | TTGCCAAGGATTTAGAGGTTCA | | GCTACTTGTGCTCCAACTTCCA |
|  | *OfRAN* | AGAACCGACAGGTGAAGGCAA | | TGGCAAGGTACAGAAAGGGCT |
| evm.model.Contig66.152 | *35s::GFP-* *OfGT3* | aagcttctgcaggggcccgggATGTTGGATAGTTCAGTTTTCTCGG | | cactagtatttaaatgtcgacCCCCATCGTTGGTAATGAAAA |
| evm.model.Contig176.19 | *35s::GFP-* *OfGT42* | aagcttctgcaggggcccgggATGTTGGCTAGTTCAGTTTTCTTGG | | cactagtatttaaatgtcgacCCCCATGGTTGACAATGGAA |
| evm.model.Contig254.65 | *35s::GFP-* *OfGT46* | aagcttctgcaggggcccgggATGTTTGATGGTATGCAGTCTGGT | | cactagtatttaaatgtcgacTTGGTTTTCCGGTTCATTATCTG |
| evm.model.Contig66.152 | pGBKT7- *OfGT3* | catggaggccgaattcccgggATGTTGGATAGTTCAGTTTTCTCGG | | atgcggccgctgcaggtcgacCCCCATCGTTGGTAATGAAAA |
| evm.model.Contig176.19 | pGBKT7- OfGT42 | catggaggccgaattcccgggATGTTGGCTAGTTCAGTTTTCTTGG | | atgcggccgctgcaggtcgacCCCCATGGTTGACAATGGAA |
| evm.model.Contig254.65 | pGBKT7- *OfGT46* | catggaggccgaattcccgggATGTTTGATGGTATGCAGTCTGGT | | atgcggccgctgcaggtcgacTTGGTTTTCCGGTTCATTATCTG |
|  | *NBL25* | ATCCTCACAGAGCGTGGTTAC | | CACTGAGCACTATGTTTCCGT |
|  | *NbSOD* | AAAGGGCACCACAGAATTAAAGTA | | GCTCCAGTGCTCCATAGTCGTA |
|  | *NbAPX* | CTCCTCCATATCCACAACAGAACTA | | GCAGAGTGCCATGCTAAACG |
|  | *NbCAT* | GTATCGTCCGTCAAGTGCCT | | CGGGAGCTCGAAGGAAATCA |
